# Calcium Supplementation Increases Blood Creatinine Concentration in a Randomized Controlled Trial

**DOI:** 10.1371/journal.pone.0108094

**Published:** 2014-10-15

**Authors:** Elizabeth L. Barry, Leila A. Mott, Michal L. Melamed, Judith R. Rees, Anastasia Ivanova, Robert S. Sandler, Dennis J. Ahnen, Robert S. Bresalier, Robert W. Summers, Roberd M. Bostick, John A. Baron

**Affiliations:** 1 Department of Community and Family Medicine, Geisel School of Medicine at Dartmouth, Lebanon, New Hampshire, United States of America; 2 Department of Medicine, Geisel School of Medicine at Dartmouth, Lebanon, New Hampshire, United States of America; 3 Departments of Medicine and of Epidemiology and Population Health, Albert Einstein College of Medicine, Bronx, New York, United States of America; 4 Department of Biostatistics, University of North Carolina, Chapel Hill, North Carolina, United States of America; 5 Department of Medicine, University of North Carolina, Chapel Hill, North Carolina, United States of America; 6 Department of Veterans Affairs Eastern Colorado Health Care System and University of Colorado School of Medicine, Denver, Colorado, United States of America; 7 Department of Gastroenterology, Hepatology and Nutrition, University of Texas MD Anderson Cancer Center, Houston, Texas, United States of America; 8 Department of Medicine, University of Iowa Carver College of Medicine, Iowa City, Iowa, United States of America; 9 Department of Epidemiology, Rollins School of Public Health, Emory University, Atlanta, Georgia, United States of America; Weill Cornell Medical College Qatar, Qatar

## Abstract

**Background:**

Calcium supplements are widely used among older adults for osteoporosis prevention and treatment. However, their effect on creatinine levels and kidney function has not been well studied.

**Methods:**

We investigated the effect of calcium supplementation on blood creatinine concentration in a randomized controlled trial of colorectal adenoma chemoprevention conducted between 2004–2013 at 11 clinical centers in the United States. Healthy participants (N = 1,675) aged 45–75 with a history of colorectal adenoma were assigned to daily supplementation with calcium (1200 mg, as carbonate), vitamin D_3_ (1000 IU), both, or placebo for three or five years. Changes in blood creatinine and total calcium concentration were measured after one year of treatment and multiple linear regression was used to estimate effects on creatinine concentrations.

**Results:**

After one year of treatment, blood creatinine was 0.013±0.006 mg/dL higher on average among participants randomized to calcium compared to placebo after adjustment for other determinants of creatinine (P = 0.03). However, the effect of calcium treatment appeared to be larger among participants who consumed the most alcohol (2–6 drinks/day) or whose estimated glomerular filtration rate (eGFR) was less than 60 ml/min/1.73 m^2^ at baseline. The effect of calcium treatment on creatinine was only partially mediated by a concomitant increase in blood total calcium concentration and was independent of randomized vitamin D treatment. There did not appear to be further increases in creatinine after the first year of calcium treatment.

**Conclusions:**

Among healthy adults participating in a randomized clinical trial, daily supplementation with 1200 mg of elemental calcium caused a small increase in blood creatinine. If confirmed, this finding may have implications for clinical and public health recommendations for calcium supplementation.

**Trial Registration:**

ClinicalTrials.gov NCT00153816

## Introduction

Calcium supplements are widely recommended and commonly used in the United States, especially by postmenopausal women for preventing and treating osteoporosis [1]–[4]. Extraskeletal health benefits of calcium have also been proposed, including a reduction in risk of colorectal neoplasms [5]–[8]. Although observational studies originally suggested inverse associations with cardiovascular disease, recent analyses of clinical trials [9]–[11] as well as a large prospective cohort study [12] suggest increased cardiovascular risk from calcium supplementation. However, another recent analysis did not find increased cardiovascular risk [13]. Additionally, two recent studies provide evidence of a reduction in all cause mortality from calcium supplementation alone [14] or with vitamin D [15]. Potential adverse renal effects of calcium supplementation include an increased risk of kidney stones [16] and renal insufficiency associated with hypercalcemia and metabolic alkalosis in the calcium-alkali syndrome [17]. Further research is needed to investigate the extraskeletal effects of supplemental calcium use.

In a randomized controlled trial of the effects of calcium and/or vitamin D supplementation on colorectal adenoma recurrence, changes in blood creatinine concentration were measured as a pre-specified interim outcome for safety to assess potential renal effects. We observed that calcium treatment for one year was associated with a small increase in blood creatinine concentration, suggesting the possibility of an effect on renal function. In the current report, we analyze the association of calcium treatment with changes in blood creatinine among the predominantly male, 45–75 year old participants in the full factorial component of our trial.

## Methods

### Ethics Statement

This study was registered at ClinicalTrials.gov as NCT00153816 (www.clinicaltrials.gov) on September 7, 2005, when the investigators first became aware of the opportunity for trial registration. The authors confirm that all ongoing and related trials for this drug/intervention are registered. The following institutional review boards (IRBs) approved the study prior to its initiation at that site (date of initial approvals are in parentheses): Dartmouth College Committee for the Protection of Human Subjects (8/25/03), University of Southern California IRB (3/17/04), Kaiser Permanente IRB (5/27/04), Colorado Multiple Institutional Review Board (7/23/04), Emory University IRB (4/12/04), Atlanta Veterans Affairs Medical Center (VAMC) IRB (4/12/04), University of Iowa IRB (6/9/04), University of Minnesota IRB (6/3/04), Minneapolis VAMC IRB (6/29/04), Portsmouth Regional Hospital IRB (3/24/04), Concord Hospital IRB (4/1/04), Maine Medical Center IRB (6/7/04), White River Junction VAMC IRB (3/4/04), University of North Carolina IRB (5/24/04), Cleveland Clinic IRB (5/21/04), University of Puerto Rico IRB (9/25/06), San Juan VAMC IRB (11/7/06), Palmetto Health IRB (4/13/04), University of Texas/MD Anderson Cancer Center IRB (4/7/04). All participants provided written informed consent at enrollment. An independent Safety and Data Monitoring Committee reviewed the study semiannually.

### Study Design

The protocol for this trial and supporting CONSORT checklist are available as supporting information; see Checklist S1 and Protocol S1. The Vitamin D/Calcium Polyp Prevention Study was a multicenter, placebo-controlled, double-blind, randomized clinical trial investigating the efficacy of vitamin D, calcium, or both for preventing sporadic colorectal adenoma recurrence. Participants were independently randomized to calcium or vitamin D in a modified two-by-two factorial design. In the “Full Factorial” component of the study, participants were randomized with equal probability to four treatment arms: 1000 IU/day vitamin D_3_, 1200 mg/day elemental calcium (as carbonate), both vitamin D_3_ and calcium, or placebo. In this report, which analyzes the effect of calcium treatment, we compare the two groups randomized to calcium (calcium alone or calcium+vitamin D) to the two “control” groups randomized to no calcium (placebo or vitamin D alone). In the “Two Arm” component of the study, women who chose to receive calcium supplementation were randomized with equal probability to either vitamin D_3_ plus calcium or to calcium alone, and are excluded from this report since they were not randomized to calcium treatment. The expected duration of the intervention was either 3 or 5 years, according to the colonoscopy follow-up interval recommended by the participants' physicians. Sample size was determined by the hypothesized treatment effect on the primary outcome of adenoma recurrence.

Participants were recruited from gastroenterology departments and practices associated with eleven clinical centers in the United States; the Project Coordination Center and trial database are located at The Geisel School of Medicine at Dartmouth.

### Eligibility, Enrollment and Randomization

Eligible patients were 45–75 years of age, in good general health, and had at least one large bowel adenoma removed at a colonoscopy within four months before enrollment with a recommended follow-up interval of either 3 or 5 years. Other inclusion criteria included blood total calcium within the normal range, creatinine ≤20% above the upper limit of normal, and 25-hydroxyvitamin D between 12–90 ng/ml. Exclusion criteria included a history of a familial colorectal cancer syndromes, invasive large bowel cancer, malabsorption syndromes, and indications or contraindications to the study agents (e.g., kidney stones, granulomatous diseases, hyperparathyroidism, hypercalcemia, osteoporosis, osteomalacia, or use of interacting drugs).

Participant recruitment began on 5/15/04 and enrollment occurred between July 16, 2004 and July 31, 2008. At enrollment, a detailed questionnaire was administered to collect demographic, medical and lifestyle data. Race and Hispanic ethnicity were self-reported. Current height and weight were documented, blood was collected, and participants completed a Block Brief 2000 Food Frequency Questionnaire (NutritionQuest, Berkeley, CA). Participants were counseled to avoid additional supplementation with calcium or vitamin D during the study and to reduce their dietary consumption of calcium if it exceeded 1200 mg/day. Study multivitamins, formulated without any calcium or vitamin D, were provided to participants if desired. At enrollment, participants began a blinded placebo run-in period (56–84 days) to assess adherence to study procedures before randomization; participants who took <80% of their study pills were not randomized. A web-based program was used to randomize participants in blocks with computer-generated random numbers; stratification factors included full factorial participation, clinical center, sex, and colonoscopic follow-up interval (3 or 5 years). Identical appearing study pills contained calcium (600 mg elemental calcium as carbonate), vitamin D_3_ (500 IU), both agents, or placebo with instructions to take one tablet twice daily with food. Participants, health care providers and investigators were blinded to treatment assignments.

### Follow-up

Participants were interviewed every six months by telephone regarding adherence to study treatment; use of medications and nutritional supplements; symptoms, illnesses, medical procedures and hospitalizations; and dietary intake of calcium and vitamin D. As pre-specified in the protocol, interim visits were scheduled to measure blood total calcium, creatinine, and 25-hydroxyvitamin D one year after randomization, and three years after randomization for participants with a 5-year follow-up. Participants were removed from treatment if their calcium was above the normal range, if their creatinine was >20% above the upper limit of normal, or if their 25-hydroxyvitamin D was <10 or>100 ng/ml. In addition, 25-hydroxyvitamin D was measured at “end-of-treatment visits” scheduled shortly before the participants' follow-up colonoscopies. Creatinine was measured at this time as well for participants who elected to take part in a “kidney sub-study” of the renal effects of vitamin D, which was active between September 2010 and April 2012. Additionally, in October 2011 the Safety and Data Monitoring Committee unblinded the study investigators to the observed association of calcium treatment with elevated blood creatinine levels. As a result, the protocol was modified to include creatinine and calcium measurements annually, to remove participants from randomized calcium treatment if their creatinine was above the upper limit of normal or if there was an increase of ≥0.3 mg/dL from baseline, and to retrospectively measure creatinine and calcium on stored specimens from “end-of-treatment visits” that were completed previously without creatinine measurements (at Quest Diagnostics, Denver, CO). Fasting prior to study blood draws was recommended but not required. Participant follow-up in the treatment phase of the study ended on August 31, 2013 and the main results are currently being analyzed.

### Laboratory Measurements

Measurements of creatinine and calcium were performed on fresh serum or plasma samples according to standard procedures at laboratories associated with the eleven participating clinical centers. Local laboratory reference ranges were used to identify out-of-range values. Three clinical centers changed laboratories during the study. Additionally, measurements of creatinine and calcium on frozen serum samples collected at “end-of-treatment visits” completed prior to October 2011 were performed at Quest Diagnostics (Denver, CO): creatinine was measured using a kinetic modified Jaffe method (Beckman Coulter Inc., Brea, CA) and total calcium was measured using an Arsenazo assay (Beckman Coulter Inc.). All 25-hydroxyvitamin D measurements were performed on frozen serum samples at the University of California (Los Angeles) at the Nutrition Research Center Laboratory using a radioimmunoassay kit from Immunodiagnostic Systems (IDS, Fountain Hills, AZ).

### Statistical Analyses

Study sample size was based on power calculations for the primary outcome of adenoma recurrence. T-tests were used to compare blood creatinine and total calcium concentrations between treatment groups. A chi-square test was used to compare the number of participants by treatment group with elevated (out-of-range) creatinine concentrations or with increases of ≥0.3 mg/dL between baseline and year one. Estimated glomerular filtration rate (eGFR) was calculated using the CKD-EPI equation [18]. Multiple linear regression was used to estimate the effect of calcium treatment on untransformed year one creatinine concentration or eGFR, adjusting for the baseline level of the respective outcomes. To generate parsimonious models, race, Hispanic ethnicity and randomized vitamin D treatment assignment were forced into the model and other participant characteristics were chosen for inclusion by backward elimination using the Akaike Information criterion. Sensitively analyses were conducted to test the robustness of the results among non-Hispanic whites only, optimally compliant participants, or participants with a year one creatinine measurement performed in the same laboratory as their baseline measurement. In exploratory analyses, effect modification was examined using Wald tests for interactions between calcium treatment and other covariates. Due to the large number of participants missing data on baseline dietary calcium intake (8%), this variable was excluded from sub-group analyses on the small number of participants with baseline eGFR<60 ml/min/1.73 m^2^. Analyses were conducted according to the intent-to-treat principle, except as indicated. All statistical tests were two-sided and considered significant at a value of P<0.05. Statistical analyses were performed using SAS (version 9.3, Cary, NC).

## Results

**Figure 1:**
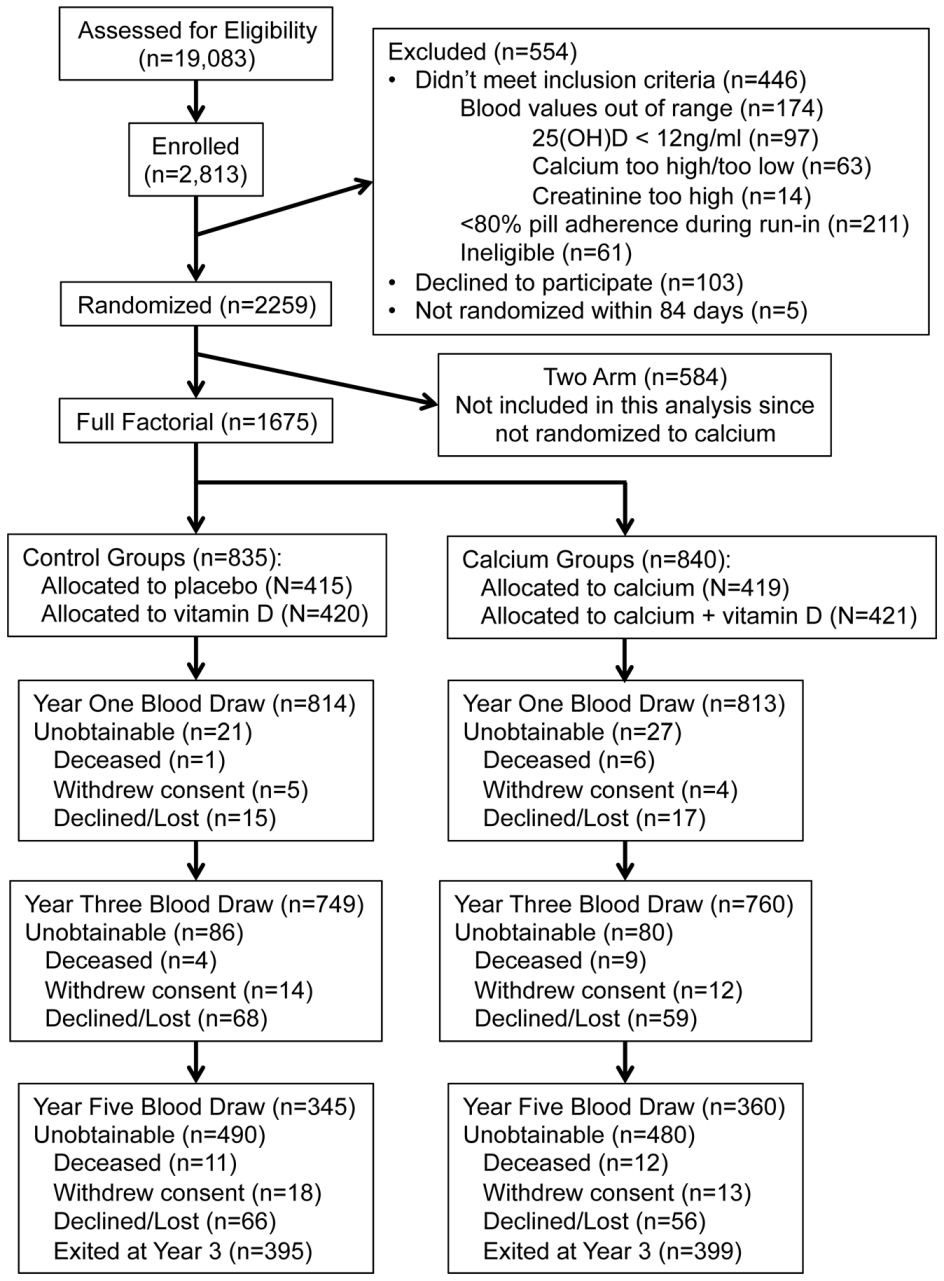
Vitamin D/Calcium Polyp Prevention Study Participant Flow Diagram.

A total of 1,675 participants were randomized in the Full Factorial component of the study (Figure 1). Baseline demographic, lifestyle and other relevant characteristics were similar between participants in the control or calcium treatment groups (Table 1). The mean age at enrollment was approximately 58 years, 85% of participants were male and 85% were Caucasian. Baseline blood creatinine, total calcium, and 25-hydroxyvitamin D concentrations were also similar among participants in the control or calcium treatment groups. A total of 28 participants (1.7%) had an elevated creatinine within 20% above the lab normal range and 66 (3.9%) had an eGFR less than 60 ml/min/1.73 m^2^, a level that, if persistent, commonly defines chronic kidney disease [18].

**Table 1:** Baseline Characteristics of Participants in the Full Factorial Component of the Vitamin D/Calcium Polyp Prevention Study, by Calcium Treatment Group (N = 1,675).

Characteristic	Control^1^ (N = 835)	Calcium^2^ (N = 840)
Sex (male)	713 (85.4)	710 (84.5)
Age (years)	58.3±7.0	58.7±6.9
Race:		
Caucasian	721 (86.4)	704 (83.8)
African American	52 (6.2)	79 (9.4)
Asian/Pacific Islander	22 (2.6)	16 (1.9)
Other/Unknown	40 (4.8)	41 (4.9)
Hispanic Ethnicity	43 (5.2)	49 (5.9)
Body Mass Index [weight (kg)/height(m^2^)]	29.1 (4.7)	29.2 (5.1)
Alcohol Intake (drinks/day):		
None	258 (31.1)	255 (30.6)
0.1–1.9	441 (53.1)	462 (55.4)
2–6	131 (15.8)	117 (14.0)
Smoking status:		
Never	404 (48.4)	416 (49.5)
Former	357 (42.8)	343 (40.8)
Current	74 (8.9)	81 (9.6)
Physical Activity: Total MET minutes per week	3197±3030	3196±2996
Taking multivitamin	432 (51.7)	442 (52.7)
Taking calcium supplement^3^	65 (7.8)	65 (7.8)
Dietary calcium intake (mg/day)	658±300	685±316
Dietary vitamin D intake (IU/day)	135±99	138±94
Blood creatinine (mg/dL)	0.97±0.16	0.97±0.17
Blood creatinine above lab normal range	13 (1.6)	15 (1.8)
eGFR^4^ <60 ml/min/1.73 m^2^	30 (3.6)	36 (4.3)
Blood total calcium (mg/dL)	9.35±0.38	9.34±0.39
Serum 25-hydroxyvitamin D (ng/ml)	24.6±8.1	24.6±8.6
Diuretic use^5^	144 (17.3)	141 (16.8)
Aspirin use daily	308 (36.9)	300 (35.7)
Non-aspirin NSAID use daily	60 (7.2)	59 (7.0)
Diabetes^6^	66 (7.9)	85 (10.1)

Data are means ± SD or number of participants (% of total).

1 Includes placebo and vitamin D treatment arms.

2 Includes calcium and calcium plus vitamin D treatment arms.

3 Taking a calcium supplement separate from a multivitamin.

4 Estimated glomerular filtration rate (eGFR) calculated using the CKD-EPI equation [18].

5 Includes use of loop, thiazide, and K+-sparing diuretics.

6 Participant self-report of diabetes diagnosis or use of medication(s) for diabetes.

Number of participants with missing data: Hispanic ethnicity 3 (1 placebo, 2 calcium); BMI 1 (placebo); alcohol 11 (5 placebo, 6 calcium); taking multivitamin 2 (calcium); taking calcium supplement 3 (1 placebo, 2 calcium); dietary calcium and vitamin D 129 (64 placebo, 65 calcium); diabetes 1 (placebo).

Numbers of and reasons for unobtainable blood draws at years one, three and five were similar between the treatment arms (Figure 1). Overall, 97.1% of participants (N = 1,627) had a year one blood draw (Figure 1), which was collected 14.7±1.4 months (mean ± SD) from the baseline blood draw. In the period between the baseline and year one blood draw, 81.6% of participants (N = 1,367) were “optimally compliant”, defined as taking at least 80% of their study pills, having no gaps in pill taking greater than 7 consecutive days, and taking no personal calcium supplements.

At year one, randomized calcium treatment was associated with a slightly larger increase in blood creatinine concentration: 0.038±0.004 versus 0.021±0.004 mg/dL (mean ± SE) (P = 0.01) (Table 2). Among participants with a normal creatinine at baseline, more in the calcium treatment group developed creatinine concentrations above the normal laboratory reference range at year one (27 participants in the calcium group and 13 in the control group, P = 0.02). In addition, more subjects in the calcium treatment group experienced creatinine increases of at least 0.3 mg/dL at year one (41 in the calcium group and 26 in the control group, P = 0.06). As expected, calcium treatment was also associated with modestly increased blood total calcium concentration (Table 2). In exploratory analyses, in the intervals between years one and three and between years three and five, there did not appear to be further increases in blood creatinine or calcium concentrations due to calcium treatment in analyses including all participants (Table 2) or in analyses restricted to participants with blood measurements performed in the same lab as at the prior time point (Table S1).

**Table 2:** Blood Creatinine and Total Calcium Measurements for Participants in the Full Factorial Component of the Vitamin D/Calcium Polyp Prevention Study, by Calcium Treatment Group.

	Control Treatment Groups^1^			Calcium Treatment Groups^2^			P^5^
Analyte	N	Analyte mg/dL^3^	Change in Analyte, mg/dL^4^	N	Analyte mg/dL^3^	Change in Analyte mg/dL^4^	
Creatinine							
Year 1, and change from baseline	814	1.00±0.17	0.021±0.004	813	1.01±0.19	0.038±0.004	0.01
Year 3, and change from year 1	749	0.96±0.23	−0.042±0.007	760	0.97±0.19	−0.045±0.005	0.68
Year 5, and change from year 3	345	0.95±0.19	−0.042±0.008	360	0.96±0.18	−0.046±0.006	0.74
Total Calcium							
Year 1, and change from baseline	814	9.30±0.37	−0.047±0.014	813	9.39±0.41	0.046±0.016	<0.0001
Year 3, and change from year 1	726	9.24±0.40	−0.062±0.016	735	9.36±0.43	−0.023±0.017	0.09
Year 5, and change from year 3	289	9.21±0.47	−0.067±0.031	301	9.38±0.41	−0.036±0.028	0.44

1 Includes placebo and vitamin D treatment arms.

2 Includes calcium and calcium plus vitamin D treatment arms.

3 Data are means ± SD.

4 Data are means ± SE.

5 T-test for comparison of change in creatinine or total calcium concentrations in control vs. calcium treatment groups.

Next, we examined the effect of calcium supplementation on year one creatinine concentration adjusted for baseline creatinine and other potential determinants of renal function (Table 3). Overall, the regression model explained 60% of the variance in year one creatinine (*r^2^* = 0.596). After controlling for characteristics listed in Table 3, year one creatinine values were on average 0.013±0.006 mg/dL (mean ± SE) higher in the calcium treatment group than the control group (P = 0.03). In sensitivity analyses, almost identical results were seen when the analysis was restricted to optimally compliant participants (0.013±0.007 mg/dL, P = 0.05), to non-Hispanic whites only (0.013±0.007 mg/dL, P = 0.06), or to participants with year one creatinine measurements performed in the same laboratory as their baseline measurement (0.015±0.006 mg/dL, P = 0.02).

**Table 3:** Adjusted Effect Measures for the Association of Participant Characteristics with Year One Blood Creatinine Concentration Using Multiple Linear Regression (N = 1,500).

Characteristics	Change^3^ (SE)	P
Baseline creatinine^1^ (per mg/dL)	0.747 (0.020)	<0.001
Age^1^ (per year)	0.0011 (0.0005)	0.01
Female sex	−0.072 (0.010)	<0.001
Race:		0.85
Caucasian	reference	
African American	0.001 (0.013)	
Asian/Pacific Islander	−0.016 (0.022)	
Other/Unknown	−0.010 (0.019)	
Hispanic ethnicity	−0.020 (0.022)	0.36
Smoking status:		0.01
Never	reference	
Former	−0.014 (0.006)	
Current	−0.031 (0.011)	
Alcohol Intake (drinks/day)		0.02
0	reference	
0.1–1.9	0.0004 (0.0071)	
2–6	−0.024 (0.010)	
Dietary Calcium Intake^1^ (per 100 mg)	−0.002 (0.001)	0.02
Diuretic Use^2^	0.015 (0.008)	0.05
Vitamin D Treatment Group	0.005 (0.006)	0.42
Calcium Treatment Group	0.013 (0.006)	0.03

Of N = 1,627 participants with year one creatinine available for analysis, 127 are not included due to missing data (see Table 1). CI, confidence interval.

Characteristics chosen for inclusion by backward elimination using the Akaike Information criterion for model selection, with race, Hispanic ethnicity and randomized vitamin D treatment group forced into the model; characteristics are from baseline, except diuretic use (see ^2^); results for study centers not shown.

1 Characteristics are modeled as continuous variables with units as shown.

2 Diuretic use between baseline and year one blood draws.

3 Change in creatinine (mg/dL) associated with the exposure (mean, coefficient).

To determine whether the concomitant increase in blood calcium mediated the effect of calcium supplementation on creatinine concentration (an increase of 0.013±0.006 mg/dL, mean ± SE), year one blood calcium concentration was added to the regression model. For every 1 mg/dL increase in year one calcium concentration there was an increase of 0.039±0.008 mg/dL (mean ± SE) in year one creatinine (P<0.0001) and the coefficient for calcium treatment decreased by about 23% to 0.010±0.006 mg/dL (mean ± SE) (P = 0.11).

Notably, randomized vitamin D treatment did not effect year one creatinine concentration (Table 3) or modify the effect of calcium treatment on year one creatinine concentration (P_interaction_ = 0.54). It also did not statistically significantly effect blood calcium concentration at year one, although it was associated with a net increase in 25-hydroxyvitamin D concentration of 6.7 ng/ml (P<0.001).

In exploratory analyses, there was suggestive evidence from subgroup analyses that two factors modified the effect of calcium supplementation. First, the effect of calcium appeared to be more pronounced among participants (N = 62) with baseline eGFR <60 ml/min/1.73 m^2^: at year one creatinine was on average 0.10±0.05 mg/dL higher (mean ± SE) (P = 0.06, P_interaction_ = 0.01) and the adjusted year one eGFR was on average 4.9±2.7 ml/min/1.73 m^2^ lower (mean ± SE) (P = 0.07, P_interaction_ = 0.10). Due to the high rate of missing data on baseline dietary calcium intake, it was excluded from this sub-group analysis; however, its inclusion did not change the results. Second, greater baseline alcohol consumption appeared to be associated with a larger effect of calcium treatment, although the interaction was not statistically significant. Among participants in the highest category of baseline alcohol consumption (2 to 6 drinks per day, N = 239), year one creatinine was on average 0.034±0.016 mg/dL (mean ± SE) higher among participants randomized to calcium treatment (P = 0.04, P_interatcion_ = 0.07 for alcohol consumption modeled as continuous variable).

## Discussion

Calcium supplementation (1200 mg/day of elemental calcium, as calcium carbonate) led to a very modest increase in blood creatinine concentration in a population of generally healthy 45–75 year old adults, predominantly white (85%) and male (85%), who were participating in a randomized clinical trial. After one year, creatinine concentration was 0.013 mg/dL higher on average among participants in the calcium treatment group compared to the control group after adjusting for other determinants of creatinine concentration. However, in exploratory analyses, the effect of calcium supplementation appeared to be more pronounced among participants with baseline eGFR <60 ml/min/1.73 m^2^ or among those consuming the most alcohol (2–6 drinks/day). There was no evidence that randomized vitamin D treatment independently affected creatinine or modified the effect of calcium supplementation. In addition, in exploratory analyses, the effect appeared to be sustained but not progressive beyond the first year of calcium supplementation.

Although numerous studies have investigated randomized calcium supplementation for osteoporosis prevention or treatment [19]–[29], and/or cancer prevention [5], [28], [30]–[32], we could only find two that reported the effect of calcium supplementation on blood creatinine. In one [33], 438 participants aged 60 years or older were randomized to placebo or 600 IU 25-hydroxyvitamin D or 750 mg elemental calcium for 4 years. The change in serum creatinine was not significantly different between the three treatment groups. However, in a study of 295 women aged 46–55 years randomized to either 1000 or 2000 mg of elemental calcium daily or no supplementation for 2 years [34], there was a small but statistically significant increase in serum creatinine in the calcium supplementation groups, in agreement with our findings.

The mechanism by which calcium supplementation increases blood creatinine is unknown, but could be due to an effect on renal function. Some of the effect could be due to the very modest increase in blood calcium due to calcium supplementation, possibly through a hemodynamic mechanism. Our statistical analyses suggest that the increase in blood calcium concentration explained only about 23% of the increase in creatinine concentration due to calcium supplementation at year one. Other potential mechanisms include mild vasoconstriction due to increased calciuria or induction of natriuresis by calcium [35], which could cause mild dehydration, or possibly increased calcification of the glomeruli. Notably, it has been suggested that glomerulosclerosis and atherosclerosis share common mechanisms [36] and increased vascular calcification has been suggested as a possible mechanism for an increase in cardiovascular events with calcium supplementation [11]. Alternatively, calcium supplementation may increase the creatinine load or change renal handling of creatinine without a direct effect on kidney function (*i.e.*, without reducing glomerular filtration). For example, treatment with an active vitamin D analogue increased creatinine production and serum creatinine without modifying the glomerular filtration rate [37]. Further research investigating other biomarkers of renal function (e.g, cystatin C) could help to distinguish between these possibilities.

The clinical relevance of the increase in creatinine associated with one year of calcium supplementation in our study is uncertain, but our exploratory analyses suggest that it may be larger and therefore more important in individuals with pre-existing renal impairment or alcohol consumption. Notably, rapid declines in kidney function of the magnitude reported here for the participants with pre-existing renal impairment (5 ml/min/1.73 m^2^) have been associated with increased cardiovascular risk and mortality [38], [39]. In addition, even among participants with normal creatinine concentration at baseline in our study, significantly more developed creatinine concentrations above the normal reference range in the calcium group compared to the control group. However, some common medications that increase creatinine are nonetheless associated with renal protection [40]. In general, more attention to potential adverse effects of dietary supplements is warranted [41], [42]. Although some evidence supports the use of calcium supplements for the prevention and treatment of osteoporosis [43], [44], the evidence for calcium alone (without vitamin D) is not very strong. Potential adverse effects include kidney stones [16], [45] and the emerging evidence on cardiovascular risk [11], [46]. Another consideration is whether supplemental calcium has different effects than calcium that is obtained from dietary sources and/or whether the effects may differ according to the timing of intake relative to ingestion of food [12], [47]–[50]. Finally, calcium-based phosphate binders are routinely used to lower phosphorus levels in patients with chronic kidney disease prior to end stage renal disease. While the effect of these binders on kidney progression has not been evaluated systematically, in an observational study in Australia, phosphate binders (type not specified) were associated with faster CDK progression [51] and in a small randomized clinical trial comparing 3 different clinically available phosphate binders, only a participant in the calcium-based binder group experienced an episode of acute kidney injury [52]. Based on our data and these other suggestive analyses, studies are needed to evaluate the effects of calcium-based phosphate binders on kidney disease progression.

The strengths of this study include the well-characterized population, with uniform and detailed collection of participant characteristics at the time of enrollment. The randomized exposure to calcium supplementation minimizes concerns about confounding suggesting that the differences we observed could be due to a causal effect. Participant compliance with study procedures was excellent, including scheduled blood draws, pill taking and avoidance of outside use of the study agents. Results are generalizable to a putatively healthy outpatient population receiving preventative health care, although the participants analyzed were predominantly male, as 70% of women female participants chose to receive calcium supplementation.

There are also several limitations. Blood creatinine concentration was used as a surrogate for renal function and we do not have data on other markers of renal function nor did we directly measure the glomerular filtration rate. In addition, creatinine measurements were performed in many different laboratories and some laboratories may have changed assays over the course of the trial. Nonetheless, the additional variability introduced should not differentially impact the randomized treatment groups and would likely have biased our results towards the null. Also, given the small effect size, we had limited power to detect effect modification in the sub-group analyses. Finally, we did not measure blood ionized calcium concentrations, and had to rely on total calcium.

In summary, results from a randomized placebo-controlled trial indicate that supplementation with 1200 mg of elemental calcium daily is associated with a small increase in blood creatinine in healthy adults. This finding is important to investigate further, considering that a large proportion of older individuals take calcium supplements [2] and chronic kidney disease is a common and growing public health threat in that population [53], [54].

## Supporting Information

Table S1 **Blood Creatinine and Total Calcium Measurements Among Participants in the Full Factorial Component of the Vitamin D/Calcium Polyp Prevention Study** ***with Blood Measurements Performed in the Same Lab at the Prior Time Point*** **, by Calcium Treatment Group.**
(DOCX)

Protocol S1 **Trial Protocol.**
(DOC)

Checklist S1 **CONSORT Checklist.**
(DOC)
