# Supplementary material for: Calcium Supplementation Increases Blood Creatinine Concentration in a Randomized Controlled Trial
Source: PLoS One. 2014 Oct 15;9(10):e108094. doi: 10.1371/journal.pone.0108094 (PMC4198086; doi:10.1371/journal.pone.0108094)
Supplement: Table S1 — Blood Creatinine and Total Calcium Measurements Among Participants in the Full Factorial Component of the Vitamin D/Calcium Polyp Prevention Study with Blood Measurements Performed in the Same Lab at the Prior Time Point , by Calcium Treatment Group. (DOCX) [file pone.0108094.s001.docx]

| **Table S1**. Blood Creatinine and Total Calcium Measurements Among Participants in the Full Factorial Component of the Vitamin D/Calcium Polyp Prevention Study ***with Blood Measurements Performed in the Same Lab at the Prior Time Point***, by Calcium Treatment Group | | | | | | | | | |
| --- | --- | --- | --- | --- | --- | --- | --- | --- | --- |
|  |  | | | |  |  | | |  |
|  | | Placebo Treatment Group^1^ | | |  | Calcium Treatment Group^2^ | | | P^5^ |
| Analyte | N | | Analyte  mg/dL^3^ | Change in  Analyte, mg/dL^4^ |  | N | Analyte  mg/dL^3^ | Change in  Analyte mg/dL^4^ |  |
| Creatinine |  | |  |  |  |  |  |  |  |
| Year 1, and change from baseline | 760 | | 1.00 ± 0.17 | 0.024 ± 0.004 |  | 770 | 1.01 ± 0.19 | 0.039 ± 0.004 | 0.02 |
| Year 3, and change from year 1 | 365 | | 1.00 ± 0.18 | 0.001 ± 0.007 |  | 362 | 1.01 ± 0.19 | 0.004 ± 0.007 | 0.76 |
| Year 5, and change from year 3 | 159 | | 0.97 ± 0.17 | -0.020 ± 0.010 |  | 161 | 0.98 ± 0.19 | -0.024 ± 0.010 | 0.75 |
| Total Calcium |  | |  |  |  |  |  |  |  |
| Year 1, and change from baseline | 760 | | 9.30 ± 0.37 | -0.046 ± 0.014 |  | 770 | 9.38 ± 0.41 | 0.047 ± 0.016 | <0.0001 |
| Year 3, and change from year 1 | 342 | | 9.26 ± 0.41 | -0.020 ± 0.021 |  | 338 | 9.40 ± 0.44 | 0.007 ± 0.024 | 0.41 |
| Year 5, and change from year 3 | 107 | | 9.28 ± 0.44 | 0.007 ± 0.035 |  | 108 | 9.44 ± 0.45 | -0.015 ± 0.043 | 0.69 |
| ^1^Includes placebo and vitamin D treatment arms.  ^2^Includes calcium and calcium plus vitamin D treatment arms.  ^3^Data are means ± SD.  ^4^Data are means ± SE.  ^5^T-test for comparison of change in creatinine or total calcium concentrations in placebo vs. calcium treatment groups. | | | | | | | | | |
