# Supplementary material for: Calcium Supplementation Increases Blood Creatinine Concentration in a Randomized Controlled Trial
Source: PLoS One. 2014 Oct 15;9(10):e108094. doi: 10.1371/journal.pone.0108094 (PMC4198086; doi:10.1371/journal.pone.0108094)
Supplement: Protocol S1 — Trial Protocol. (DOC) [file pone.0108094.s002.doc]

## Protocol for:

# Vitamin D/Calcium Polyp Prevention Study

**Principal Investigator:**

John A. Baron. M.D.

# Geisel School of Medicine at Dartmouth

Biostatistics & Epidemiology

Evergreen Center, Suite 300

46 Centerra Parkway

Lebanon, NH 03766

# 603-650-3456 (Phone)

603-650-3473 (Fax)

[John.A.Baron@dartmouth.edu](mailto:John.A.Baron@dartmouth.edu)

**NCI Grant Number: R01 CA098286-01A1**

**Project Coordination Center:**

Geisel School of Medicine at Dartmouth, Lebanon, New Hampshire

**Study Centers:**

Dartmouth Hitchcock Medical Center, Lebanon, New Hampshire

Cleveland Clinic Foundation, Cleveland, Ohio

University of Colorado, Denver, Colorado

Emory University, Atlanta, Georgia

University of Iowa, Iowa City, Iowa

University of Minnesota, Minneapolis, Minnesota

University of North Carolina, Chapel Hill, North Carolina

University of South Carolina, Columbia, South Carolina

University of Southern California, Los Angeles, California

University of Texas, Houston, Texas

University of Puerto Rico/ Puerto Rico Medical Center, San Juan

**Current Date and Protocol version number: 1-9-14 version 18.0**

**Previous Date and version number:** **9-30-13 version 17.0**

**Study agents:** Calcium carbonate, Vitamin D_3_

**IND number:** 68,361

# TABLE OF CONTENTS

Page

1. Objectives …………………………………………………………………………………. 4
2. Background and Rationale ………………………………………………………………… 4
3. Summary of Study Plan …………………………………………………………………… 12
   1. Overview ………………………………………………………………………… 12
   2. Schema ………………………………………………………………………….. 13
4. Subject Selection and Enrollment ………………………………………………………… 14
   1. Study Population ………………………………………………………………... 14
   2. Participating Centers ……………………………………………………………. 14
   3. Sources and Methods of Recruitment …………………………………………... 16
   4. Enrollment Procedures …………………………………………………………. 17
   5. Run-in procedures and Randomization Assessment …………………….…….. 18
   6. Method of Subject Identification ………………………………………………. 19
   7. Inclusion Criteria ……………………………………………………………….. 19
   8. Exclusion Criteria ………………………………………………………………. 20
5. Agent Information and Administration …………………………………………………… 21
   1. Name of Agents and Study Arms ………………… ……………………………. 21
   2. Assignment and Duration of Study Treatment …………………..……………… 21
   3. Dose Selection – Safety and Efficacy ……………………………………..…….. 22
   4. Formulation ……………………………………………………………………… 22
   5. Administration …………………………………………………………………... 22
   6. Side Effects ……………………………………………………………………... 22
   7. Contraindications ……………………………………………………………….. 23
   8. Manufacturer and Supplier ……………………………………………………… 23
   9. Packaging, Labeling and Distribution ………………………………………… 23
   10. Storage ………………………………………………………………………….. 24
   11. Blinding and Unblinding Methods …………………………………………… 24
   12. Adherence/Compliance …………………………………………………………. 24
   13. Agent Accountability ………………………………………………………….. 25
   14. Agent Disposal …………………………………………………………………. 25
   15. Assessment of Blinding Effectiveness ……………………………………….. 25
6. Post-randomization Follow-up ……………………………………………………………. 26
   1. Interval questionnaires ……………………………………………………………. 26
   2. Colds and Flu questionnaires……………………………………………………… 26
   3. Schedule of Lab Measurements……………………………………………………. 28
   4. Colonoscopy ………………………………………………………………………. 30
   5. SF-36 Health Survey ……………………………………………………………. 30
   6. End of Treatment Visit ……………………………………………………………. 30
   7. End of Treatment Questionnaire ………………………………………………….. 31

6.8 End of Treatment Phase…………………………………………………………… 31 6.9 Observational Follow Up ………………………………………………………………….. 31

1. Clinical Procedures – Endoscopy …………………………………………………………. 32
   1. Overview of Procedure …………………………………………………………. 32
   2. Quality Assurance ………………………………………………………………… 33
2. End-point Determination and Pathology …………………………………………………. 33
3. Laboratory Measurements – Blood Samples ……………………………………………… 34
   1. Laboratories ……………………………………………………………………… 34
   2. Collection and Handling Procedures …………………………………………….. 34
   3. Methods for Laboratory Procedures …………………………………………….. 35

10.0 Ancillary Studies……………………………………………………………………… 36

10.1 Dartmouth Polyp Prevention Biospecimen Repository…………………….. 36

10.2 Biospecimen Analyses……………………………………………………… 36

10.2.1 Genetic variant influencing response to vitamin D and calcium ………………….. 36

10.2.2 Early detection of colorectal neoplasia with ctDNA………………….................... 37

10.2.3 Metabolomic Study of the Effects of Vitamin D and Calcium Supplementation… 37

10.2.4 Vitamin D_3_, Calcium and Biomarkers of Inflammation and Gut Barrier Function 38

10.3 Data Analyses……………………………………………………….……… 37

10.4 Calcium/Vitamin D, Biomarkers, and Colon Polyp Prevention……………… 37

10.5 Vitamin D/Calcium Mammography Study…………………………………… 42

10.6 Kidney Function with Vitamin D/Calcium…………………………… ..……. 47

10.7 Participant Beliefs and Bias Study……………………………………………. 53

10.8 Colds and Flu Study…………………………………………………………… 55

11.0 Safety Monitoring and Reporting Adverse Events ……………………………………… 57

11.1 Definition, Monitoring and Follow-up ………………………………………. 57

11.2 Adverse Event Information and Severity …………………………………….. 57

11.3 Serious Adverse Events ……………………………………………………… 58

11.4 Reporting …………………………………………………………………….. 58

12.0 Data Management ……………………………………………………………………… 58

12.1 Central Database ……………………………………………………………… 58

12.2 Website ………………………………………………………………………. 58

12.3 Data Entry …………………………………………………………………… 58

12.4 Computer Security and Safety……………………………………………….. 59

12.5 Archiving ……………………………………………………………………. 59

12.6 Reports ………………………………………………………………………. 59

13.0 Statistical Methods …………………………………………………………………….. 60

13.1 Hypotheses ………………………………………………………………….. 60

13.2 Sample Size Justification …………………………………………………… 60

13.3 Methods for Randomization and Stratification ………………………………. 62

13.4 Outcome Measures ………………………………………………………….. 62

13.5 Statistical Analysis ………………………………………………………….. 62

13.6 Assumptions …………………………………………………………………. 64

13.7 Compliance and Missing Data …………………………………………….…. 64

13.8 Interim Analyses ……………………………………………………………… 64

13.9 Ancillary Studies …………………………………………………………….. 64

14.0 Ethical and Regulatory Considerations ………………………………………………… 65

14.1 Form FDA 1572 ……………………………………………………………… 65

14.2 IRB Approval ………………………………………………………………… 65

14.3 Informed Consent ………………………………………………………….…. 68

14.4 Safety and Data Monitoring Plan ………………………………………….…. 68

14.5 Project Coordination Center, Sponsor or FDA Monitoring ……………….. 69

14.6 Record Retention ……………………………………………………………. 69

14.7 Certificate of Confidentiality …………………………………………………. 69

15.0 References ……………………………………………………………………..……. 70

1. ***Objectives***

There is compelling evidence that most colorectal cancers arise from adenomatous tissue, suggesting that prevention of adenomas would prevent colorectal cancer. Our research group recently documented that calcium supplementation inhibits the development of adenomas in the large bowel, and there is good evidence that vitamin D may also protect against colorectal neoplasia. We will now extend our work by studying the chemopreventive effects of vitamin D on colorectal neoplasia, and by investigating whether calcium in combination with vitamin D is more effective than calcium alone in suppressing colorectal neoplasia.

This is a randomized, double-blind, placebo-controlled chemoprevention trial, investigating whether supplementation with calcium carbonate and/or vitamin D will reduce the risk of adenomas of the large bowel. Our main hypotheses are:

1. Supplementation with vitamin D_3_ (1000 IU/day) reduces the risk of new adenomas in patients with a recent history of these tumors.

2. Supplementation with calcium carbonate (1200 mg elemental calcium/day) reduces the risk of new adenomas

3. Supplementation with both vitamin D_3_ (1000 IU/day) and calcium carbonate (1200 mg elemental calcium/day) reduces the risk of new adenomas more than supplementation with calcium carbonate alone.

Our secondary hypotheses are:

4. Supplementation with vitamin D_3_, or with vitamin D_3_ plus calcium carbonate, reduces the risk of new advanced colorectal lesions (colorectal cancer and adenomas with an estimated diameter of one centimeter or greater, those with tubulovillous or villous histology, or those with advanced dysplasia).

5. Supplementation with vitamin D_3_ will have a greater effect among individuals whose initial serum 25-OH vitamin D levels are less than the overall study median, compared with subjects whose levels are greater than the median.

6. The effect of supplementation with vitamin D_3_ will be modified by polymorphisms at the 3' end of the vitamin D receptor gene.

**In addition to these primary and secondary hypotheses, we will also investigate benefits and risks associated with calcium and vitamin D supplementation.**

***2.0 Background and Rationale***

There is clear evidence that colorectal cancer, the most common visceral cancer in the United States,[^1^](#_ENREF_1) is potentially preventable. Neoplastic polyps (adenomas) of the bowel are recognized pre-malignant lesions and effective endpoints for chemoprevention studies. Randomized intervention studies among patients with adenomas can greatly clarify the effects of intake of specific nutrients on the risk of colorectal neoplasia. Epidemiological and experimental data indicating that both vitamin D and calcium exert anti-neoplastic effects in the large bowel are described below. However, evidence from clinical trials is required to clarify the synergistic and independent chemopreventive effects of these two agents. This study is a multi-centered randomized, double-blind, partial factorial trial of the efficacy of calcium carbonate and vitamin D in preventing the occurrence of neoplastic polyps of the large bowel.

**Vitamin D and Colorectal Carcinogenesis**

Vitamin D and its “Classical” Effects

Vitamin D refers to a family of related steroid hormones best known for its important role in maintaining calcium and phosphorus homeostasis, and in regulating bone metabolism. In its classical actions, vitamin D increases intestinal calcium absorption, decreases serum parathyroid hormone (PTH) levels (thus inhibiting bone resorption) and decreases urinary calcium excretion. Markedly depressed vitamin D status causes osteomalacia, a condition characterized by a deficiency of bone matrix that has severe consequences for bone structure. Mildly depressed vitamin D status seems to predispose to osteoporosis, a condition in which total bone volume is reduced but the bone matrix remains relatively normal.[^4^](#_ENREF_4)

There are two precursors to active vitamin D hormones. Vitamin D_3_ (cholecalciferol) is synthesized in the skin after exposure to ultraviolet light, and may also be obtained from some dietary sources. A related steroid, vitamin D_2_ (ergocalciferol), is derived solely from the diet. Since the biological activities of these two compounds are thought to be equivalent, they are often referred to together as “vitamin D,” a convention we follow here. Both compounds are metabolized to the most active steroid, 1,25-(OH)_2_ vitamin D, through successive hydroxylation reactions: first (25-hydroxylation) in the liver, and second (1α-hydroxylation) in the kidney. Since 25-hydroxylation is not closely regulated, 25-OH vitamin D levels reflect overall vitamin D status from combined dietary and sunlight sources. In contrast, 1α-hydroxylation is tightly regulated to maintain homeostasis of calcium, magnesium and phosphorus. Consequently, serum levels of 1,25-(OH)_2_ vitamin D do not reflect vitamin D status except during clear conditions of deficiency or excess.[^5^](#_ENREF_5) Both hydroxylation reactions can occur outside the liver and kidney -- for example by activated macrophages or by lymphoma cells -- but these do not affect circulating vitamin D levels in the absence of diseases such as sarcoidosis, other granulomatous diseases, or lymphoma.[^6-8^](#_ENREF_6)

Although 1,25-(OH)_2_ vitamin D is much more potent than 25-OH vitamin D on a molar basis (by a factor of 100 to 1000), the higher circulating levels of 25-OH vitamin D (nanogram concentrations, rather than picogram) may compensate for its lower potency. Thus 25-OH vitamin D seems to have a direct role in calcium metabolism, for example increasing calcium absorption.

There is relatively little vitamin D in natural foods, and for some humans, sunlight is the predominant source. There is some vitamin D in fatty fish and eggs; the amounts vary depending on the time of year the food was harvested and/or the supplementation status of the animals. In the U.S., milk and breakfast cereals are fortified, but many other developed countries do not fortify any foods. In practice, even in the U.S., fortification is quite variable. Although toxicity from over-fortification has been reported, the added vitamin D is often less than the stated amount and some “fortified” foods have been found to contain no measurable vitamin D.

The Vitamin D Receptor

Like all steroid hormones, vitamin D acts through a nuclear ligand-activated receptor, the vitamin-D receptor (VDR), which operates as a heterodimer with the retinoid X receptor (RXR). Levels of expression of the VDR may have clinical implications: it has been suggested that reduced expression of the VDR in the bowel may explain the decrease in calcium absorption seen with aging.[^4^](#_ENREF_4) Despite the importance of the VDR, rapid actions through a cytoplasmic receptor have also been identified.[^13^](#_ENREF_13)

The VDR contains several polymorphisms. Four of these, near the 3' end of the gene, are in strong linkage disequilibrium with each other: a poly-A microsatellite (3'UTR) and variants identified with the enzymes BsmI (intron 8), Apa I (intron 8), and TaqI (exon 9).[^14-17^](#_ENREF_14) Because of the linkage disequilibrium, in most ethnic groups there are essentially only two VDR 3' haplotypes. These appear to have functional significance, since the polymorphisms have been associated with physiological measurements such as bone density and circulating 1,25-(OH)_2_ vitamin D levels. However, these polymorphisms are apparently just markers of another functionally active site that has not yet been identified. There is also a polymorphic site at the 5' end of the VDR gene, recognized by FokI, in the initiation codon. This polymorphism, in contrast to the other VDR variants, results in an alteration in the expressed amino acid sequence.[^14^](#_ENREF_14)

Vitamin D and Carcinogenesis

The expression of the nuclear vitamin D receptor in virtually all tissues of the body (including the bowel mucosa) suggests a role for vitamin D beyond mineral regulation. In particular, recent in-vitro studies show that vitamin D and vitamin D analogues can modulate cellular growth and proliferation. In human cell lines from the colon and other organs, these compounds inhibit proliferation, induce differentiation and promote apoptosis.[^23-27^](#_ENREF_23) The antiproliferative effects of vitamin D are apparent in psoriasis: topical and oral vitamin D has been used to treat this hyperproliferative disorder,[^28-30^](#_ENREF_28) and the Apa I polymorphism in the VDR has been associated with risk of the disease.[^31^](#_ENREF_31)

Clearly, vitamin D signaling can have anti-proliferative effects on the large bowel mucosa, causing, for example, inhibition of proliferation in human rectal mucosal explants[^32^](#_ENREF_32) and in biopsies from patients with ulcerative proctocolitis.[^33^](#_ENREF_33) Vitamin D supplementation normalizes the colonic crypt hyperproliferation of vitamin-D deficient animals,[^34^](#_ENREF_34) and inhibits induction of mucosal ornithine decarboxylase by bile acids[^35^](#_ENREF_35) or the bowel carcinogen DMH.[^36^](#_ENREF_36) The VDR is expressed in neoplastic colorectal tissue, and expression of the VDR has been correlated with the degree of differentiation of colon carcinoma cell lines.[^39^](#_ENREF_39) High receptor expression has been associated with a good prognosis in colorectal cancer.[^40^](#_ENREF_40) In an intervention trial in humans, rectal mucosal proliferation was found to be inversely related to 25-OH vitamin D levels.[^41^](#_ENREF_41)

Furthermore, various vitamin D hormones can inhibit experimental carcinogenesis. 1,25-(OH)_2_ vitamin D_3_ suppresses the growth of solid tumors derived from human cancer cells,[^42^](#_ENREF_42) and correction of vitamin D deficiency (together with calcium supplementation) decreased experimental bowel carcinogenesis in one rodent model.[^43^](#_ENREF_43) 1,25-(OH)_2_ vitamin D and synthetic analogues reduce polyp size and number in Min mice.[^44^](#_ENREF_44) Even in animals that are vitamin-D replete, experimental bowel carcinogenesis has been suppressed by supplementation with 1-(OH) vitamin D,[^45^](#_ENREF_45) vitamin D_3_[^46^](#_ENREF_46) and 1,25-(OH)_2_ vitamin D. There are some negative data, however.[^48^](#_ENREF_48)

Several molecular mechanisms seem to underlie these effects. Ligand-bound VDR can arrest cells in the G1 stage of the cell cycle, probably through modulation of cell cycle proteins such as cyclin D1.[^49-51^](#_ENREF_49) Vitamin D signaling has important connections to several growth factor pathways. It interferes with epidermal growth factor (EGF) signaling (perhaps by reducing expression of the EGF receptor), reduces expression of the insulin-like growth factor-1 receptor,[^49^](#_ENREF_49) and inhibits IGF-1 signaling generally.[^54^](#_ENREF_54) Vitamin D also can reduce expression of c-myc, c-fos and c-jun oncogenes, and suppress telomerase and angiogenesis. The apoptotic effects of these hormones may occur through induction of bak and transforming growth factor β or through inhibition of expression of bcl-2. Vitamin D signaling also seems to play a role in the metabolism (and detoxification) of bile acids in the bowel.[^56^](#_ENREF_56) Finally, vitamin D appears to have important immunological effects, including modulation of T-cell-mediated immunity.

Vitamin D and Human Colorectal Cancer

The earliest indications that vitamin D might protect against colorectal carcinogenesis were ecological analyses showing that in the U.S., the risk of colorectal cancer is inversely associated with average sunlight patterns. More compelling evidence is provided by data regarding dietary vitamin D intake, which has repeatedly been inversely associated with colorectal cancer [^61-66^](#_ENREF_61) and colorectal adenomas, although there are also studies that do not support an association.[^68-73^](#_ENREF_68) There is likely to be a conservative bias in these studies: inconsistent vitamin D fortification and variable amounts in natural foods create considerable measurement error in dietary assessment.[^8^](#_ENREF_8) Furthermore, the fact that in the U.S. dietary intake of both calcium and vitamin D is largely derived from dairy products makes it difficult to separate the effects of these two nutrients.

Investigations of supplemental or total vitamin D intake point even more clearly to a protective effect of vitamin D. This is not surprising, since such studies focus on a wider, higher range of intake than dietary studies, and recall of vitamin D supplement use is likely to be more precise than dietary assessment. Investigation of serum vitamin D brings further advantages, since 25-OH vitamin D levels take into account sunlight exposure and are not dependent on dietary recall. 25-OH vitamin D levels have repeatedly been inversely associated with risk of colorectal neoplasia. As discussed above, 1,25-(OH)_2_ vitamin D levels are tightly regulated and do not reflect vitamin D status; associations with this steroid have not been particularly striking. Interactions of vitamin D with calcium are discussed further below. The association of vitamin D receptor polymorphisms with the risk of colorectal neoplasia has been investigated in a few studies. Overall, the FokI polymorphism seems unrelated to risk,[^83-85^](#_ENREF_83) but one investigation reported an association with large adenomas among subjects with low calcium or vitamin D intake.[^85^](#_ENREF_85) The variant alleles of the 3' polymorphisms have more clearly been related to colon cancer, particularly among subjects with low calcium or vitamin D intake.[^86^](#_ENREF_86) In our Calcium Polyp Prevention Study, however, the VDR variants did not appear to modify the association of vitamin D with adenoma risk. In this study, we will assay TaqI polymorphisms (at the 3’ end of the gene) as well as the (5’) FokI polymorphisms. We hypothesize that the former – but not the latter – will modify the effect of vitamin D treatment.

The epidemiological data described above suggest that circulating 25-OH vitamin D levels are more closely associated with colorectal cancer risk than are the highly regulated 1,25-(OH)_2_ vitamin D levels. This is a plausible pattern for two reasons. As noted above, the circulating concentrations of 25-OH vitamin D, approximately 3 orders of magnitude higher than those for 1,25-(OH)_2_ vitamin D, seem to be high enough to overcome their relatively low affinity for the VDR. Also, there is emerging evidence that 1α-hydroxylation is constitutively expressed in normal, adenomatous, and malignant colon tissue[^87-90^](#_ENREF_87) (H. Cross, personal communication). Thus high levels of circulating 25-OH vitamin D have the potential to increase intracellular levels of the more active 1,25-(OH)_2_ form. A similar situation holds for prostate cancer, since human prostate cancer cell lines have been shown to 1α-hydroxylate vitamin D,[^91^](#_ENREF_91) and in these cell lines, 25-OH vitamin D inhibits proliferation with potency similar to 1,25-(OH)_2_ vitamin D. Thus vitamin D may be effective for prostate cancer chemoprevention even in vitamin D-replete men. These patterns indicate that doses of vitamin D that are *not* associated with hypercalcemia may nonetheless have important chemopreventive effects.

Vitamin D Dose

The Institute of Medicine recently increased the recommended daily vitamin D intake for adults to 600 IU per day among those over 50 years old and 800 IU/day for those over 70.[^94^](#_ENREF_94)The NOAEL (no observed adverse effect level) was increased to 4000 IU/day for adults. [^94^](#_ENREF_94)

No one has investigated the optimal vitamin D dose or serum level for anti-neoplastic effects in humans. From the point of view of bone health, vitamin D levels should be high enough to suppress PTH levels and thus minimize bone resorption. Frank vitamin D deficiency with hyperparathyroidism tends to occur with 25-OH vitamin D levels less than about 30 nmol/L.[^95^](#_ENREF_95) However, suppression of PTH does not occur until 25-OH vitamin D levels are above 75-100 nmol/L.[^96-98^](#_ENREF_96) These levels become more difficult to sustain among older persons. With age, there is a decrease in the ability of the skin to produce vitamin D_3_, a factor that compounds the diminishing periods of time spent outdoors with aging. Moreover, intestinal absorption of vitamin D is impaired in the elderly, and renal 1-hydroxylation of vitamin D declines with age.

Multivitamins and calcium/vitamin D supplements typically provide 400 IU per day, but numerous intervention studies (reviewed in) show that this dose will not suppress PTH in the overwhelming majority of North American adults. A more effective dose is 1000 IU per day, which will raise 25-OH vitamin D levels toward the desired range, and leave a substantial margin of safety, even after taking into consideration dietary intake. The rate of increase in 25-OH vitamin D levels after daily vitamin D is admittedly variable, but most studies indicate that 80-90% of the steady state level is achieved within a few months.[^101-106^](#_ENREF_101) One investigation found that both older and younger subjects responded similarly to a dose of 800 IU of vitamin D_3_ daily, slightly below the dose we propose.[^101^](#_ENREF_101)

Safety of Vitamin D Supplementation

In sufficiently high doses, vitamin D is toxic, largely because of the induction of hypercalcemia, which can lead to renal toxicity (decreased GFR and diminished concentrating capability), metastatic calcification, nausea, vomiting, and CNS effects.[^8^](#_ENREF_8) In doses not associated with hypercalcemia, vitamin D probably has no toxicity.

We will use vitamin D_3_ as a supplement, thereby avoiding the toxicity risks associated with 1,25-(OH)_2_ vitamin D or 25-OH vitamin D. On theoretical grounds, this approach is appealing, since the supplementation would be with a pro-hormone, a choice that takes advantage of natural metabolism to generate the most active moiety. Supplementation with even large doses of vitamin D does not increase total 1,25-(OH)_2_ vitamin D levels in individuals who are not vitamin D deficient,[^4^](#_ENREF_4) although toxicity may still ensue.

Vitamin D in doses of 800-1800 IU/day has been found to be safe in several studies (largely investigations of treatment or prevention of osteoporosis), even when given with 1000 mg or more of calcium per day. Several of these studies had treatment periods between 2 and 3 years. Vitamin D doses more than twice as large as that proposed are considered safe[^8^](#_ENREF_8) and carry a large margin of safety. A 1-year pilot study of the regimen which we will use -- 1000 IU of vitamin D administered with 1200 mg of calcium – indicated no toxicity (H. Newmark, personal communication).

Exposure to sunlight or UV radiation does not in itself lead to vitamin D toxicity, since after a minimal erythema dose, further exposure does not lead to increased 25-OH vitamin D levels; increased degradation counterbalances increased production. Therefore, even with cutaneous production of vitamin D, the dose we propose will not lead to toxicity among individuals who do not otherwise have disturbances of calcium metabolism. (Individuals with such disturbances will be excluded from the study.)

Even for the general population, vitamin D supplementation is likely to be beneficial. One study of older Europeans showed that 36% of men and 47% of women had vitamin D deficiency (25-OH vitamin D levels < 30 nmol/L).[^95^](#_ENREF_95) In a younger French population (median age 50 years), 14% were found to be vitamin D deficient and 75% had non-suppressed parathyroid hormone (25-OH vitamin D levels below 78 nmol/l).[^97^](#_ENREF_97) Even in sunny countries, low vitamin D levels may be surprisingly common (almost 3/4 of individuals in one study) because of avoidance of direct sun exposure.

Despite supplementation of milk and breakfast cereals, suboptimal vitamin D status is also common in the U.S. The median intake in young U.S. women has been estimated at 114 IU/day, and only 90 IU/day in older women, both well below recommended levels.[^8^](#_ENREF_8) Among medical inpatients in Boston, 57% were found to have hypovitaminosis D (defined as 25-OH vitamin D levels < 37 nmol/L).[^117^](#_ENREF_117) Even among “healthy” patients under 65 years old (not homebound or chronically ill), 42% were vitamin D deficient, as were 37% of those with estimated vitamin D intakes exceeding the recommended 600 IU/day. Over 90% had 25-OH vitamin D levels below 75 nmol/L. Among participants in the Women's Health and Aging Studies (based on a random sample of Medicare beneficiaries), 45% of women with no or minimal disability had 25-OH vitamin D levels at or below 50 nmol/L.[^118^](#_ENREF_118) In our Calcium Polyp Prevention Study, 4.9% of subjects were frankly vitamin D deficient at baseline, and 54% likely had non-suppressed parathyroid hormone (25-OH vitamin D levels < 75 nmol/L).

**Calcium and Colorectal Neoplasia**

Bile Acids, Calcium and Colorectal Carcinogenesis

A consistent finding in animal studies is that bile acids and soluble fatty acids in the stool promote colorectal carcinogenesis, probably through irritative/proliferative effects on the bowel mucosa.[^119-123^](#_ENREF_119) Almost twenty years ago it was proposed that calcium in the water phase of the stool could bind and precipitate these carcinogenic factors, thus rendering them relatively inactive and sparing the mucosa from their adverse effects. Although direct effects of calcium have been proposed, animal studies have generally supported such an intraluminal, indirect mechanism. Precipitated fat "soaps" have been observed in stool after calcium supplementation, and dietary calcium can ameliorate the hyperproliferative effect of carcinogens, bile acids or small bowel resection on the bowel mucosa. Moreover, in a variety of animal models, calcium intake has protected against experimental bowel carcinogenesis, particularly among rats fed a high-fat diet. Some studies suggest a greater effect of calcium on carcinomas than on adenomas, and on the number of tumors per tumor-bearing animal, rather than on the proportion bearing any tumor.[^43^](#_ENREF_43)

Calcium Intake and Human Colorectal Neoplasia: Observational Data

In contrast to the animal data, human epidemiological studies regarding dietary calcium intake and colorectal cancer have yielded mixed findings. Some cohort studies found suggestions of an inverse association of dietary or total calcium intake with colorectal cancer risk, but several have been negative.[^139-142^](#_ENREF_139) Case-control studies have reported similarly inconsistent results, with findings ranging from a marked inverse association (e.g.,[^143^](#_ENREF_143)) to suggestions of an increased risk with high dietary intake.[^63^](#_ENREF_63) Epidemiological investigation of colorectal adenomas has also been conflicting, as have studies regarding the effects of calcium supplements on colorectal neoplasia.

Clinical Trials of Calcium and Colorectal Carcinogenesis

The strongest support for a protective effect of calcium intake on colorectal neoplasia has come from randomized chemoprevention trials, which avoid many of the difficulties of observational investigation. In a clinical trial conducted by our research group, we studied the effect of 4 years of supplementation with 1200 mg daily of elemental calcium among patients with a recent history of colorectal adenomas. Calcium treatment reduced the risk of any recurrent adenoma by 17% (95% CI 2-33%), and reduced the numbers of recurrent adenomas by 25% (95% CI 4-40%). There was a more pronounced effect on lesions with advanced features (54% risk reduction, 95% CI 17-74%). Very similar findings were reported from a smaller European trial[^153^](#_ENREF_153) in which calcium supplementation (2 gm/day) conferred a 25% (95% CI -29% to 57%) reduction in risk.

Two other, smaller, clinical trials also used calcium, though in quite different contexts. In a study among patients with FAP, calcium supplementation (600 mg/day) provided no clear benefit with regard to polyp counts, although the treated group did have a smaller increase in polyp number than the placebo group.[^154^](#_ENREF_154) Another trial of 116 patients who had small (<10 mm) polyps left in place, tested a mixture of antioxidants (selenium, beta carotene, alpha tocopherol and ascorbic acid) and calcium (1.6 gm/day). The mixture had no effect on the growth of existing polyps, but significantly reduced the risk of new adenomas (risk ratio = 0.70).[^155^](#_ENREF_155)

Investigations of the effect of calcium supplementation on proliferative indices have been inconsistent; some studies reported no beneficial effect,[^156-162^](#_ENREF_156) but others found a reduction in proliferation.[^163-168^](#_ENREF_163) Trials of other biological markers have also been inconsistent. In some investigations, anti-carcinogenic effects were observed: a reduction in the cytotoxicity of fecal water, a reduced proportion of secondary bile acids in the bile acid pool, and lowered bile acid concentration in fecal water.[^171^](#_ENREF_171) However, other investigations reported contrary findings: no change, or an increase in the concentration of bile acids in the water phase of stool.

Interactions of Vitamin D and Calcium

Vitamin D and calcium are intertwined physiologically, and an important research goal is understanding their independent and synergistic effects. One experimental study in rodents found that calcium and vitamin D_3_ supplementation together had a smaller protective effect than either supplement alone.[^134^](#_ENREF_134) However, most studies have reported that vitamin D supplementation has a stronger anti-neoplastic effect in animals given relatively high-calcium diets. Because of the measurement error associated with these two exposures, it is to be expected that human observational studies may have difficulty clarifying any interactive effects. Indeed several studies seem to suggest that the effects of dietary calcium and vitamin D are independent of each other. However, in two large cohort studies with careful dietary assessment, there was clear evidence of a positive interaction between the two nutrients. Also, in our Calcium Polyp Prevention Study (see Preliminary Studies, below), there were strong indications that vitamin D enhanced the chemopreventive effect of calcium. Among subjects whose baseline 25-OH vitamin D levels were below the median, calcium had no effect on risk of one or more adenomas after randomization (adjusted risk ratio 1.06; 95% CI 0.82-1.22). In contrast, among subjects with 25-OH vitamin D levels above the median, calcium substantially lowered the risk of adenoma occurrence (adjusted risk ratio for calcium 0.71; 95% CI 0.57-0.88; p for interaction = 0.01). As expected, the calcium effect was similar in subjects with higher and lower levels of 1,25-(OH)_2_ vitamin D. These important data suggest that vitamin D and calcium may work together to reduce the risk of colorectal neoplasia.

Calcium Chemoprevention

The positive clinical trial findings regarding calcium chemoprevention of large bowel neoplasia are exciting and important from both scientific and public health points of view. They help clarify contradictory observational studies, provide clues to the etiology of colorectal neoplasia, and advance the identification of effective chemopreventive strategies.

Calcium supplementation is widely used and is safe. The Institute of Medicine’s recommended calcium intake for adults 19 through 50 years old is 1000 mg/day, for adults 51 through 70 years it is1200 mg/day for women and 1000 mg/day for men, and for those over 70 years it is 1200 mg/day.[^94^](#_ENREF_94) Intake up to 2000 mg/day in adults over 50 is considered safe.[^94^](#_ENREF_94) The main potential toxicities of calcium supplementation are urinary stones and hypercalcemia (which can lead to renal insufficiency and alkalosis, the “milk-alkali syndrome”). We will exclude from our study all subjects with renal insufficiency or a history of kidney stones. Milk alkali syndrome is rare in healthy people, and has not been noted in trials of calcium and vitamin D supplementation that used doses similar to those we propose.

**Summary**

Extensive experimental and observational data suggest that intake of both calcium and vitamin D exert protective effects on colorectal neoplasia. Building on our previous work, we will now investigate the chemopreventive effect of vitamin D in the large bowel, to study whether both agents together are more effective than calcium alone, and to confirm our positive finding regarding calcium. Our goal is the development of chemopreventive combinations that will reduce risk of colorectal neoplasia sufficiently to permit the lengthening of surveillance intervals in most patients and to clarify important issues regarding the mechanisms of colorectal carcinogenesis and chemoprevention.

***3.0 Summary of Study Plan***

*3.1 Overview*

This study is a double-blind, placebo-controlled trial of vitamin D and/or calcium supplementation for the prevention of large bowel adenomas. Subjects will be recruited from 11 Study Centers in North America. Eligible subjects will have had at least one large bowel adenoma removed in the 4 months prior to study entry and no remaining polyps in the bowel after complete colonoscopic examination. Participants will be randomized in a modified 2 x 2 factorial design to vitamin D (1000 IU/day), calcium carbonate (1200 mg elemental calcium/day), both agents, or placebo only. Women who decline to forego calcium supplementation will be randomized only to calcium alone or to calcium plus vitamin D. Randomization will be stratified by gender, Study Center of recruitment, and anticipated follow-up interval (see below), and will be conducted separately for female subjects randomized only to vitamin D. Participants will agree to avoid taking study agents outside the trial and will initially be observed in a 3-month placebo run-in period to identify (and exclude before randomization) subjects likely not to adhere to the study regimen. We anticipate enrolling about 3000 participants to reach a total of approximately 2400 randomized subjects. As safety measures, blood levels of calcium, creatinine and 25-(OH)-vitamin D will be obtained at baseline and 1 year after randomization, as well as 3 years after randomization for subjects with a 5-year surveillance cycle. Every six months after randomization subjects will complete a questionnaire regarding compliance with study agents, use of medications and vitamin/mineral supplements, illnesses, hospitalizations, and dietary intake of calcium and vitamin D.

The endpoint of the study will be new adenomas detected on follow-up colonoscopy. These examinations are scheduled to occur either 3 years or 5 years after the qualifying examination, depending on the follow-up interval recommended by each patient's endoscopist. Some patients may, for medical reasons, have a colonoscopy at a time other than 3 or 5 years after the qualifying examination. Information from these exams will be included in analyses where appropriate. In the primary analyses, the occurrence of new neoplastic polyps in the interval between randomization and the follow-up exam will be compared between subjects randomized to vitamin D (with or without calcium) versus those randomized to placebo (with or without calcium), between subjects randomized to calcium (with or without vitamin D) versus those randomized to placebo (with or without vitamin D, excluding women electing to receive calcium and therefore cannot participate in the calcium component of the study), and between those randomized to calcium plus vitamin D versus those randomized to calcium alone. In secondary analyses, we will examine the effect of calcium plus vitamin D versus vitamin D alone, and the impact of baseline vitamin D levels and VDR polymorphisms on the vitamin D effects. Effects on advanced adenomas will also be assessed.

*3.2 Schema*

# Colorectal Chemoprevention with Calcium and Vitamin D

# John A. Baron, Dartmouth Medical School

Men and women with a recent colonoscopy in which an adenoma was removed,

with acceptable baseline measurements of calcium, creatinine and 25-(OH)-vitamin D

Three month RUN-IN period (about 3000 participants)

Randomize about 2400 men and women to:

placebo (360 men and 84 women) or

1200 mg elemental calcium (360 men and 84 women) or

1000 IU vitamin D (360 men and 84 women) or

both agents (360 men and 84 women) daily

-OR-

Randomize women who want to take calcium to:

1200 mg elemental calcium (312 women) or

1200 mg elemental calcium and 1000 IU vitamin D (312 women) daily

Subject questionnaires at 6-month intervals

Measurements of calcium, creatinine and/or 25-(OH)-vitamin D at year 1, year 3 and, if applicable, year 5

Follow-up colonoscopy at 3 or 5 years

Number and type of new colorectal adenomas found

***4.0 Subject Selection and Enrollment***

*4.1 Study Population*

Study subjects will be healthy volunteers who have received preventive care from a gastroenterology clinic. They will be recruited to the study because they are at risk for future adenomas based on the occurrence of at least one previous adenoma removed at a surveillance colonoscopy (qualifying colonoscopy) occurring within the previous four months. Recruited subjects will be between 45 and 75 years of age at enrollment. Current preventive health care guidelines suggest an initial screening colonoscopy at 50 years of age. Individuals younger than 45 are excluded because adenomas occurring in that age group may be due to the presence of a familial colon cancer syndrome. Thus, they are likely to represent a different population with a different mechanism. The upper age limit for inclusion in the trial is used to increase the likelihood of successful compliance and completion of this long-term chemoprevention trial.

Individuals of both genders and all racial and ethnic populations will be encouraged to participate in the trial. Based on previous trials, we anticipate that approximately 60% of enrolled subjects will be males and 40% females. This accords with the demographics of adenoma prevalence, which is somewhat higher among men. We expect the ethnic and racial make-up of the subject population to approximate that of the U.S. population. The diversity of populations in the catchment areas of our 11 Study Centers makes this a realistic goal.

*4.2 Participating Study Centers and Recruitment Sites*

Subjects will be recruited from the gastrointestinal and surgical services of the eleven participating Study Centers. Principal Investigators (PI) for each Study Center are provided below. Recruitment or referral will also occur from ancillary sites, including hospitals, clinics, cancer centers, university medical practices, Veterans Affairs Medical Centers, health maintenance organizations, and private gastroenterology practices associated with these centers, as listed below. Those recruitment sites listed below that have an Institutional Review Board (IRB) that provides human subject’s oversight for this study that is separate from that of the Study Center are indicated with an asterisk.

1. Dartmouth Hitchcock Medical Center, Lebanon, NH

(PI: Douglas Robertson, MD)

- 1. Dartmouth-Hitchcock Manchester, Manchester, NH
  2. Dartmouth-Hitchcock Keene, Keene, NH
  3. Portsmouth Gastroenterology, Portsmouth, NH*
  4. Portland Gastroenterology Association, Portland, ME*
  5. Concord Gastroenterology, PA, Concord, NH*
  6. VA Medical Center, White River Junction, VT

1. Cleveland Clinic Foundation, Cleveland, OH

(PI: Gerald Beck, PhD)

- 1. Cleveland Clinic- Beachwood Family Health Center, Cleveland, OH
  2. Cleveland Clinic-Independence Family Health Center, Independence, OH
  3. Cleveland Clinic-Lorain Family Health Center, Lorain, OH
  4. Cleveland Clinic-Strongsville Family Health Center, Strongsville, OH
  5. Cleveland Clinic-Chagrin Falls Family Health Center, Chagrin Falls, OH
  6. Cleveland Clinic-Elyria Family Health Center, Elyria, OH
  7. Cleveland Clinic-Lakewood Family Health Center, Lakewood, OH
  8. Cleveland Clinic-Solon Family Health Center, Solon, OH
  9. Cleveland Clinic-Westlake Family Health Center, Westlake, OH
  10. Cleveland Clinic-Willoughby Hills Family Health Center, Willoughby Hills, OH
  11. Metrohealth Medical Center, Cleveland, OH*
  12. Cleveland Clinic – Wooster Family Health Center, Wooster, OH

1. University of Colorado, Denver, CO

(PI: Dennis Ahnen, MD)

- 1. Rocky Mountain Gastroenterology, Aurora, CO
  2. Rocky Mountain Gastroenterology, Lakewood, CO
  3. South Denver Gastroenterology, Denver, CO
  4. University Hospital, Aurora, CO
  5. Arapahoe Gastroenterology, Denver, CO
  6. Arapahoe Gastroenterology, Littleton, CO
  7. VA Medical Center, Denver, CO

1. Emory University, Atlanta, GA

(PI: Michael Goodman, MD, MPH)

- 1. VA Medical Center, Decatur, GA*
  2. Consultative Gastroenterology, Atlanta, GA

1. University of Iowa, Iowa City, IA

(PI: Robert W. Summers, MD)

- 1. Gastroenterologists, P.C.I, Cedar Rapids, IA
  2. Internists, Cedar Rapids, IA
  3. Gastroenterology Assoc. of Iowa City, Iowa City, IA
  4. Cedar Valley Medical Specialists, Waterloo, IA
  5. Dubuque Internal Medicine, Dubuque, IA
  6. Iowa Clinic, Gastroenterology, Des Moines, IA
  7. Iowa Digestive Disease Center, PC, Des Moines, IA

1. University of Minnesota, Minneapolis, MN

(PI: Tim Church, PhD)

- 1. Minnesota Gastroenterology, Minneapolis, MN
  2. VA Medical Center, Minneapolis, MN*

1. University of North Carolina, Chapel Hill, NC

(PI: Robert S. Sandler, MD)

- 1. Cary Gastroenterology Association, Cary, NC
  2. Regional Gastroenterology Association, Durham, NC

1. University of South Carolina, Columbia, SC

(PIs: Roberd M. Bostick, MD, March E. Seabrook, MD)

- 1. Consultants in Gastroenterology, West Columbia, SC

1. University of Southern California, Los Angeles, CA

(PI:Jane Figueiredo, PhD)

- 1. Los Angeles County Hospital, Los Angeles, CA
  2. South Bay Gastroenterology Medical Group
  3. Kaiser/Sunset, Los Angeles, CA*

1. University of Texas, Houston, TX

(PI: Robert Bresalier, MD)

- 1. MD Anderson Cancer Center, Houston, TX
  2. Medical Center Endoscopy, Houston, TX

11. University of Puerto Rico, San Juan, PR

(PI: Marcia R. Cruz-Correa, MD, PhD)

- 1. VA Caribbean Healthcare System, San Juan, PR*
  2. Active GI Group, San Juan, PR
  3. Instituto de Gastroenterología, San Juan, PR
  4. Hato Rey Medical Gastroenterology, Hato Rey, PR

e. Grupo Gastroenterologico del Este, Humacao PR

*4.3 Sources and Methods of Recruitment*

Study coordinators or treating medical staff will identify potential subjects through review of colonoscopy schedules and pathology reports at the participating centers and associated practices. All potential subjects must have had at least one adenoma that is at least 2 mm in size removed from the large bowel in the four months before entry into the study. For these diagnoses, we will accept the readings of the local pathologists. For individuals who meet this initial criterion, a preliminary determination of eligibility will then be made through review of available medical records and contact with the participating physicians. Those individuals who continue to be eligible following this review will be assigned a study ID number for tracking and reporting purposes upon submission of preliminary eligibility data to the Dartmouth Project Coordination Center. This eligibility data will include the individual’s date of birth and the date of the qualifying colonoscopy exam (components of a limited data set as defined by HIPAA) as permitted by local regulatory requirements.

Study Centers will implement the following process as permitted/required by local IRBs: Potential subjects will be mailed a packet of information about the study, preferably from their personal physician or clinical staff with whom they are familiar. This packet (packet #1) will include:

Introductory letter with a short description of the study and the name of the study coordinator who will be contacting the patient to determine their interest in participation.

Post card that may be returned to decline further contact regarding the study or to provide information about a good time to reach them by phone

Study brochure describing the study in more detail

Medical release authorization (may be required to verify eligibility)

The study coordinator will phone those individuals who do not decline further contact within 14 days to discuss their interest in the study, to confirm eligibility and to set up an intake appointment for those individuals who are interested in study participation. A second information packet (packet #2) will be mailed to these individuals containing:

- Letter confirming the intake appointment date and time
- Checklist of what to bring to the intake appointment
- Block Food Frequency questionnaire
- RAND SF-36 Health Survey
- Copy of the study’s Informed Consent document

Brochure from the NIH/NCI called “Taking Part in Clinical Trials: Cancer Prevention Studies – What Participants Need to Know “ or brochure from The Office for Human Research Protections called “Becoming a Research Volunteer: It's Your Decision”

*4.4 Enrollment Procedures*

An intake appointment will be scheduled within 120 days of the qualifying colonoscopy for those individuals who continue to be interested in study participation. At the intake appointment, an informed consent discussion will take place between the prospective participant and the study coordinator. After obtaining signed consent (the date of the signed consent is the Enrollment Date for the participant), the study coordinator will confirm eligibility and document the subject’s general demographic information, general health and medical history including previous colorectal procedures and adenomas, use of nutritional supplements and medications, recent sun exposure and exercise, smoking history, and family history of cancer or adenomas (the Intake Questionnaire). The responses to these questions will be entered by the study coordinator into a secure web-based form that transmits data directly to the Dartmouth Project Coordination Center in real-time over the Internet. The participant will also be asked to complete and submit to the study coordinator a Block Food Frequency Questionnaire and an SF-36 Health Survey. The Block Food Frequency Questionnaire is a validated self-administered questionnaire that is intended for epidemiologic and clinical use and captures an individual’s usual dietary food intake. The SF-36 Health Survey is a validated 36-item self-administered questionnaire on health status that assesses eight different health concepts and includes perceptions of both physical and mental health.

In a final component of the intake appointment, the study coordinator will measure and document the participant’s current height and weight, and obtain the baseline blood draw. Whenever possible, this should be a fasting blood draw (the participant should not eat food or drink beverages other than water for 8 hours prior to the blood draw). Regardless, the time of the last food or beverage intake will be collected. Pre-packaged bar-coded blood collection kits will be provided for each participant. Standard and detailed operating procedures will be provided by the Dartmouth Project Coordination Center for adequate and safe collection, processing, storage and shipment of these biospecimens.

Upon completion of the intake appointment, all participants will be given a study packet (packet #3) containing:

- Copy of the signed and dated informed consent form
- Participant Information Booklet
- Phone card -optional (Additional phone cards will be sent or given to subjects from the Study Center on a yearly basis.)
- 7-day pill dispenser
- Bottle of run-in study pills
- Study Identification Card
- Bottle of multivitamins without any vitamin D or calcium -optional per subject request (Additional bottles will be sent or given to subjects from the Study Center as needed.)
- Participant Drug Information Card (optional per local IRB requirement)

The study coordinator will ask the participant to take the first study pill. The coordinator will document the taking of the first pill at the intake appointment or ask the participant to confirm by postcard or telephone if taken later. All prospective participants who complete the intake appointment will be reimbursed $100 to defray the time and expense of the recruitment/enrollment activities.

The study coordinator will ship blood samples for Vitamin D measurements to the UCLA Nutrition Research Center at the next scheduled shipment date. (The UCLA laboratory will provide the results of these measurements directly to the Project Coordination Center.) In addition, within 15 days after the intake appointment, the study coordinator will submit to the Dartmouth Project Coordination Center the qualifying pathology and endoscopy reports and the results of the local lab measurements of calcium and creatinine. These lab results and clinical reports will be required to confirm the eligibility of the participant prior to randomization. If the results for calcium or creatinine are outside of the range for eligibility (see section 4.7 below) then the subject and their health care provider will be notified of the result in writing. If the result for 25-(OH)- vitamin D is outside of the range for eligibility (see section 4.7 below), then the subject and their health care provider will be notified in writing whether the result is “too high” or “too low.” The exact measurement will not be provided because these study measurements are not performed in a CLIA certified lab and so can be used for research purposes only. The health care providers for the affected subjects will be advised to obtain a 25-(OH)- vitamin D level at a CLIA-approved laboratory if further assessment is thought to be indicated.

Other information to be sent to the Project Coordination Center includes the completed Food Frequency Questionnaire, which will be mailed, and the SF-36 Health Survey, which will be mailed until the web form is available for data entry and transmission over the internet. These must be received by the Project Coordination Center within 60 days of the intake appointment. Finally, with the consent of the participant, the coordinator will also ship (within 60 days of the intake appointment) blood samples to the Clinical & Molecular Pharmacology Shared Resource of the Norris Cotton Cancer Center to be stored for future research on colorectal neoplasia and other disorders common in the study population.

Quality Control. The Dartmouth Project Coordination Center will review the pathology and endoscopy reports to confirm the Study Center’s assessment of eligibility before the prospective participant is randomized.

*4.5 Run-in period and randomization assessment*

At the intake appointment, or immediately upon taking the first run-in pill, the participants will enter an approximately three month long run-in period during which adherence to study agents will be assessed. Approximately 10 weeks after the intake appointment, the study coordinator will contact participants by phone to determine and document continued interest in the study, any change in eligibility or exclusionary criteria, and run-in pill taking compliance. Subjects will be asked to count the number of pills left in their pill bottles and report this number to their study coordinator who will enter it into a web form that will be used to calculate compliance. Participants whose pill counts indicate that they have taken less than 80% of their study pills will not be randomized. Participants will also be given their first ‘interval’ questionnaire on this phone call (see section 6.1 below). This phone call must occur between 56 and 84 days of the intake appointment. After this phone call, participants who continue to be eligible for and interested in study participation will be sent a new supply of study pills by mail from the Dartmouth Project Coordination Center. The new supply will be the randomly assigned study agent. Participants will be provided a postcard or telephone number to report the date the first pill was taken from this shipment. It should be taken within 98 days of the intake appointment. Study coordinators will follow-up to collect this date if the patient forgets to report it.

*4.6 Method of Subject Identification*

All potentially eligible individuals identified by a review of colonoscopy and pathology records will be assigned a study identification number prior to initial patient contact. The ID number will be in the form SC - RS - ##### . The “SC” is a number ranging from 01-99 and identifies the Study Center from which the subject was recruited. The “RS” is a number ranging from 01- 99 and corresponds to the specific recruitment site. The “#####” ranges from 00001-99999 and identifies the individual subject. This number is generated using a modulo 11 method that prevents the occurrence of sequential numbers in order to reduce errors from typing mistakes. If an individual is subsequently enrolled in the study then this same ID number will be used throughout the study. In addition, upon enrollment the subject will also be assigned a two letter study identification code consisting of the first initial of the subject’s first name followed by the last letter of the subject’s last name. This code will be used along with the subject’s study ID number in communications between the Dartmouth Project Coordination Center and the Study Center coordinators.

*4.7 Inclusion criteria*

1. One or more histologically verified neoplastic polyp (adenoma) that is at least 2 mm in size removed from the large bowel with the entire large bowel examined by colonoscopy and documented to be free of further polyps or areas suspicious for neoplasia within 120 days of study entry
2. Anticipated colonoscopic follow-up three years or five years after the qualifying colonoscopy
3. Age between 45 and 75 years at enrollment
4. (Women)Agreement to avoid pregnancy (i.e. use of standard contraception)
5. Willingness to forego calcium supplementation (including multivitamins containing calcium) or, for women only, option of taking calcium supplementation of 1200 mg/daily (contained in the study pills)
6. Willingness to forego vitamin D supplementation (including multivitamins containing vitamin D)
7. Agreement to daily dietary intake of the equivalent of not more than 1200 mg calcium
8. Agreement to daily dietary intake of the equivalent of not more than 400 IU vitamin D
9. Blood calcium level within normal range
10. Blood creatinine level not to exceed 20% above upper limit of normal
11. Serum 25-(OH)-vitamin D within lower limit of 12 ng/ml to upper limit of 90 ng/ml

12) Ability and willingness to follow the study protocol, as indicated by provision of informed consent to participate

13) Good general health, with no severely debilitating diseases or active malignancy that might compromise the patient's ability to complete the study

*4.8 Exclusion criteria*

(General exclusionary criteria:)

1. Participation in another colorectal (bowel) study (intervention study) in the past 5 years
2. Current participation in any other clinical trial (intervention study)
3. Pregnancy or lactation
4. A diagnosis of narcotic or alcohol dependence in the past 5 years
5. A diagnosis of dementia (e.g. Alzheimers) in the past 5 years
6. A diagnosis of a significant psychiatric disability (e.g. Schizophrenia, refractory bipolar disorder, current severe depression) in the past 5 years

(Exclusions due to derangement of calcium metabolism or indications/contraindications to study agents)

1. Any diagnosis of kidney stones
2. A diagnosis of granulomatous diseases, e.g. sarcoidosis, active chronic fungal or mycobacterial infections (tuberculosis, histoplasmosis, coccidioidomycosis, blastomycosis), Berylliosis, Wegener’s granulomatosis in the past 5 years
3. A diagnosis of hyperparathyroidism or other serious disturbance of calcium metabolism in the past 5 years
4. A diagnosis of severe kidney disease, e.g. chronic renal failure in the past 5 years
5. A diagnosis of unexplained hypercalcemia in the past 5 years
6. Any diagnosis of osteoporosis with physician recommendation for treatment of low bone mass
7. A diagnosis of two or more low trauma fractures in the past 5 years
8. A diagnosis of a medical condition requiring treatment with vitamin D (e.g. osteomalacia) in the past 5 years

(Exclusions due to intestinal or bowel problems:)

1. Any diagnosis of invasive carcinoma of the large bowel (even if confined to a polyp)
2. Any diagnosis of familial colorectal cancer syndromes, e.g. Familial Adenomatous Polyposis (FAP) (including Gardner syndrome, Turcot’s syndrome), Hereditary Nonpolyposis Colorectal Cancer (HNPCC), Hamartomatous Polyposis syndromes (including Peutz-Jeghers or Familial Juvenile Polyposis)
3. Any diagnosis of inflammatory bowel disease, e.g. Crohn’s Disease, Ulcerative Colitis
4. A diagnosis of chronic intestinal malabsorption syndromes, e.g. celiac sprue, bacterial overgrowth, chronic pancreatitis, pancreatic insufficiency in the past 5 years
5. Any large bowel resection

(Exclusions due to poor health:)

1. A diagnosis of malignancy, other than non-melanoma skin cancer in the past 5 years
2. A diagnosis of severe lung disease – class 3 or 4 (e.g. COPD or emphysema requiring oxygen) in the past 5 years
3. A diagnosis of severe heart disease: Cardiovascular disease functional class 3 or 4 in the past 5 years
4. Any diagnosis of severe liver disease, e.g. Cirrhosis

(Exclusions due to shipping regulations:)

1. Any current/past HIV positive diagnosis
2. Active hepatitis B, defined as : Hep B surface antigen positive

Active hepatitis C, defined as : measurable HCV RNA

(Drug exclusions:)

1. Use of chronic oral cortico-steroid therapy in the past 5 years
2. Use of Lithium in the past 5 years
3. Use of Phenytoins in the past 5 years
4. Use of Quinidine in the past 5 years
5. Use of therapeutic vitamin D in the past 5 years

***5.0 Agent Information and Administration***

- 1. *Name of agents and study arms*

There will be four arms in the study:

1. placebo
2. calcium carbonate (3 gm/daily; 1200 mg elemental calcium/daily)
3. vitamin D_3_ (1000 IU/daily)
4. calcium carbonate and vitamin D_3_

*5.2 Assignment and Duration of Study Treatment*

Men will be randomly assigned to each of the treatment groups at equal frequency. Due to recommendations for high calcium intake among post-menopausal women and the common use of calcium supplements in that population group, women will have the option of being randomized to calcium or to calcium plus vitamin D (arm 2 or 4). In this way, they will receive 3 gm of supplemental calcium carbonate daily as part of the study. Based on our experience in the Aspirin-Folate Polyp Prevention Study, we assume that 35% of women will accept the full factorial randomization and 65% will wish to continue calcium supplementation and accept the restricted randomization (two arm). There will be separate randomization procedures for subjects being randomized to both agents and for women taking calcium who will be randomized only to vitamin D. Although it will not be encouraged, at any time after enrollment, women who are in the Full Factorial component of the study will have the option to change their mind and switch to the 2 Arm component of the study. Study tablets shipped to these subjects will be changed accordingly. For statistical analyses purposes they will be analyzed as per the original treatment group that they were randomized to. All randomization will be stratified by Study Center of recruitment and scheduled colonoscopic follow-up (3 years or 5 years).

The follow-up interval for colonoscopy recommended by a patient's endoscopist, either 3 or 5 years, will determine the duration of exposure to the study agents. For a 3 year follow-up interval, the duration of exposure to the study agents will be approximately two and one half years (30 months) due to the subtraction of the initial time required for subject identification and recruitment (up to four months) and a subsequent three month run-in period during which subjects take placebo pills (full factorial) or calcium alone (two arm). Likewise, the duration of exposure to the study agents will be approximately 4 and one half years (54 months) for a 5 year follow-up interval.

*5.3 Dose selection – safety and efficacy*

Both agents have a considerable margin of safety in the doses to be used. The calcium dose of 1200 mg has been widely used; in our previous trial, this dose did not confer any toxicity. This dose is very likely to be effective. Our previous clinical trial results, together with human dietary epidemiology, indicate that the calcium dose will be associated with a decreased risk.

The vitamin D dose, 1000 IU daily, has been shown to be safe in intervention studies in humans. Together with the vitamin D in diet (typically 200-300 IU), the total vitamin D intake will be well below the 2000 IU dose set as the “no observed adverse effect level,” a standard that is itself conservative. This dose is also likely to be effective. Human epidemiological data suggest that doses of vitamin D in the range of high dietary intake or common supplemental doses may decrease the risk of colorectal neoplasia.

*5.4 Formulation*

We will use calcium carbonate for the calcium supplementation. This compound provides the convenience of supplying 1200 mg of elemental calcium in two tablets; other calcium salts would require a larger number of pills.

We will use vitamin D_3_ for the vitamin D supplementation. This will avoid the greater toxicity risks associated with 1,25-(OH)_2_ vitamin D or 25-OH vitamin D. This form of vitamin D is a pro-hormone that must be metabolized by normal mechanisms in the body to generate the most active moiety. Supplementation with even large doses of vitamin D_3_ does not increase total 1,25-(OH)_2_ vitamin D levels in individuals who are not vitamin D deficient, although toxicity may still ensue.

Each pill will contain either placebo or elemental calcium (600 mg) and either placebo or vitamin D (500 IU).

*5.5 Administration*

The study agents will be taken orally, twice a day, preferably with meals. Food increases the natural acidity of the stomach thereby enhancing calcium carbonate absorption.

*5.6 Side effects*

Calcium supplements are generally well tolerated. Use of calcium carbonate reportedly may cause such gastrointestinal side effects as bloating, gas and flatulence. Prolonged use of large doses of calcium carbonate –greater than 12 grams daily- may lead to the milk-alkali syndrome, nephrocalcinosis and renal insufficiency.

Dosage of vitamin D up to 2,400 IU/day in healthy individuals rarely causes adverse reactions. Chronic use of very high doses (3,800 IU/day or greater) of vitamin D may cause hypercalcemia. Early symptoms of hypercalcemia include nausea and vomiting, weakness, headache, somnolence, dry mouth, constipation, metallic taste, muscle and bone pain. Late symptoms and signs of hypercalcemia include polyuria, polydipsia, anorexia, weight loss, nocturia, conjunctivitis, pancreatitis, photophobia, rhinorrhea, pruritis, hyperthermia, decreased libido, elevated BUN, albuminuria, hypercholesterolemia, elevated ALT and AST, ectopic calcification, nephrocalcinosis, hypertension and cardiac arrhythmias.

*5.7 Contraindications*

Calcium and vitamin D supplementation are contraindicated in those with hypercalcemia. Conditions causing hypercalcemia include sarcoidosis, hyperparathryroidism, hypervitaminosis D and cancer. Calcium and vitamin D supplementation are also contraindicated in those hypersensitive to any component of the supplement.

*5.8 Manufacturer and supplier*

Study agents will be manufactured by Pfizer Pharmaceuticals, previously Wyeth Consumer Healthcare, which will prepare them to our specifications.

*5.9 Packaging, labeling and distribution*

Run-in and randomized treatment pills will be shipped from Pfizer Pharmaceuticals, previously Wyeth Consumer Healthcare, to the packaging facility, which will oversee packaging into white 400 cc bottles. The study agent will be packaged to prevent contamination and unacceptable deterioration during transport and storage. Bottles will be packaged with 250 pills each. The packaging facility will ship the filled bottles by group and lot number to the Dartmouth Project Coordination Center’s pharmacy office, where labels will be generated and applied to the bottles under the supervision of the study pharmacist.

*Run-in Pills*: Bottles containing run-in pills (placebo or calcium alone) will be labeled with the study name and bottle number (date). Upon obtaining all required Study Center documentation (e.g. local IRB approval), the bottled run-in pills will be sent from the Dartmouth Project Coordination Center’s pharmacy office to the Study Center’s pharmacy or study office for storage and distribution to participants upon completion of their intake appointment. A space will be provided on the label for the study coordinator or pharmacist to write the participant’s name after the bottle has been assigned to a participant. Bottles will be shipped as needed to ensure an adequate supply at each Study Center for participant enrollment.

*First Randomized Pill Bottle*: Participants who continue to be eligible for and interested in study participation at the end of the run-in period will be sent a new supply of study pills from the Dartmouth Project Coordination Center immediately after the randomization assessment phone call. The new supply will be the randomly assigned study agent. Subjects will be instructed to start this new bottle immediately upon receipt and to record the date that the bottle is started. The randomized pill bottles will be labeled with the participant’s name and shipped directly to the participant from the Project Coordination Center.

*Subsequent Randomized Pills*: Subjects will be instructed to finish their old bottle of study pills prior to starting their new one and to record the date that the new bottle is started. Participants will be provided a postcard or telephone number to report the date the first pill was taken from each interval shipment bottle. Bottles containing randomized pills will be mailed in approximately four month intervals adjusting for the previous pill bottle start date. Special shipments will also be made upon request, if, for example, a participant loses their bottle or does not receive their new bottle of pills.

*Final Randomized Pills:* For subjects still on treatment 5 months prior to the end of the treatment phase of the study on 12/1/13, the following additional steps will be taken.  Participants will receive a letter with their study pills instructing them to discontinue pill taking as of 12/1/13.  The pill bottle will also be labeled "Discard after 12/1/13".

FDA labeling requirements will also be followed in accordance with Investigational New Drug regulations, as applicable.

Quality Control. After randomization, pill content will be tested periodically (approximately annually) under the supervision of the study pharmacist on about 25 random participants in each of the four treatment groups to monitor errors in agent manufacturing, packaging, labeling and distribution procedures.

*5.10 Storage*

Bottles shipped from the packaging facility to the Dartmouth Project Coordination Center will be stored under the supervision of the study pharmacist in a secure area in the pharmacy office. The study pharmacist will supervise acceptable storage temperatures, conditions (e.g., protection from light), and storage times. Bottles shipped from the Dartmouth Project Coordination Center to the Study Centers (run-in pill bottles only) will be stored under the supervision of the local pharmacist in a secure area at the center’s pharmacy, or they may be stored under the supervision of the study coordinator in a secure area at the local study office. The Study Center pharmacist or coordinator will supervise acceptable storage temperatures, conditions, and storage times*.*

*5.11Blinding and unblinding methods*

Neither the staff of the Study Center or the study participants will know the treatment category to which a participant has been assigned. At the Dartmouth Project Coordination Center, all staff, except for the study pharmacy staff and some statistical and informatics staff, will also be blinded to the treatment categories to which participants are assigned.

Upon distribution of the initial randomized pills from the Dartmouth Project Coordination Center, a sealed envelope for each participant marked “top security”, containing the treatment assignment, will be sent concurrently to the Study Center under the supervision of the pharmacist or study coordinator.

It may be necessary to break the blinding for a study participant if he/she develops hypercalcemia or symptoms of hypercalcemia or if his/her physician requests this information, generally in an emergency situation. In this event, designated Study Center personnel may open a participant’s envelope to determine his/her treatment group. Procedures will be provided to the staff for opening, reporting and returning opened envelopes to the Dartmouth Project Coordination Center. At the end of the study, the unopened envelopes will be returned to the Project Coordination Center for inspection and destruction.

Subjects may be notified of their randomized treatment assignments upon completion of all follow-up colonoscopies at Year 5. At that time all study staff may also be unblinded to treatment assignments.

*5.12 Adherence/compliance*

Adherence to treatment will be assessed in three ways: 1) Participant-reported compliance: every 6 months, during the telephone interview with the study coordinator, the participant will be asked to report how many pills they took per week on average since their last phone interview. A report of 14 pills would represent 100 % compliance, 13 pills would represent 93 % compliance, etc... 2) Computed compliance based on participant-reported pill bottle start dates: participants will be asked to record and report to their study coordinator the date that they start each new bottle of study pills. Given the start date of each pill bottle and the total number of pills in each bottle (which the participant will be instructed to finish prior to starting the next bottle) we will calculate a compliance rate for each bottle. 3) Serum 25-OH vitamin D levels will be measured at baseline and at Years 1 and 3, and, where applicable, at year 5. To maintain blinding, these data will only be used to measure compliance at the level of treatment groups. The Project Coordination Center will regularly summarize the pill-taking status of each center's subjects, and list for each coordinator the relevant study ID numbers of those who have reported changed or inadequate adherence.

*5.13 Agent accountability*

The Dartmouth Project Coordination Center and the Study Centers, through written standard operating procedures, will ensure the adequate and safe receipt, handling, storage, dispensing, and return and/or disposal of unused study agents by study staff and study participants in compliance with applicable regulatory requirements.

*5.14 Agent disposal*

The Dartmouth Project Coordination Center and the Study Centers, through written standard operating procedures, will maintain a system for the disposal of unused or expired study agents and for the documentation of this disposal.

*5.15 Assessment of blinding effectiveness*

In a randomized placebo-controlled trial, the validity of the results depends on the effectiveness of methods to blind participants to the randomization assignment. Unblinding is a potential source of bias because someone who believes they are getting the active treatment may experience or report better outcomes. To assess unblinding, this study includes a longitudinal evaluation of blinding effectiveness as well as an assessment of the effect of participants' beliefs about the intervention on measured and self-reported health outcomes in each intervention arm.

Participants will be asked at Intake which pill they would prefer to receive if they had a choice. They will also be asked to describe their beliefs about the efficacy of calcium and vitamin D in relation to general health, prevention of colorectal polyps, and improvement of mood, depression, constipation, and bone and joint pain. After the run-in period they will be asked to guess which pill they think they have received and the reasons for their guess. In addition, subjects will be asked to complete two more questionnaires, by mail or telephone interview, at the end of year 2 and at the time of the participants' follow-up colonoscopy (Year 3 or 5). These questionnaires will be administered by independent staff from Geisel School of Medicine at Dartmouth and not by the study coordinators, because the coordinators help to collect endpoint data and could be biased by participants' responses.

***6.0 Post-randomization Follow-up***

The study coordinators at each Study Center will maintain regular contact with all subjects. Scheduled contacts with the subject are listed below. Some subjects, however, will be contacted more frequently to encourage adherence to treatment or to manage individual problems.

Follow-up activities once a subject is randomized will include:

- Every six months - interval questionnaire
- At years 1 and (where applicable) year 3 - laboratory measurements of calcium, creatinine, and vitamin D
- At Year 3 or Year 5 – follow-up colonoscopy, SF-36 Health Survey, End of Treatment Questionnaire, and laboratory measurement of vitamin D

All participants who complete Year 3 or Year 5 follow-up activities will be reimbursed $100 to defray the time and expense of these activities.

*6.1 Interval Questionnaires*

Every 6 months, all subjects will have a telephone interview regarding somatic symptoms possibly related to study agents, illnesses, doctor’s visits, hospitalizations, medications, over-the-counter preparations (including nutritional supplements), dietary intake of calcium and vitamin D, and compliance with study tablets. The responses to these questions will be entered by the study coordinator into a secure web-based form that transmits the data directly to the Dartmouth Project Coordination Center in real-time over the Internet. If access to the internet is interrupted or temporarily unavailable, a paper form will be used and the data will subsequently be entered into the web form. The interval questionnaire interview may also be performed in person when in conjunction with a Year 1 or Year 3 blood draw, as appropriate. If necessary (e.g., a subject is unavailable by phone or deaf), a subject may self-complete a paper questionnaire and return it to the study coordinator for data entry into the web form. Finally, subjects who have access to the internet will be able to self-complete the questionnaire on the web once that technology is available in the study. The interval questionnaire should be collected within 30 days on either side of each 6 month anniversary of the randomization date.

- 1. *Colds and Flu Questionnaires*

Subjects will be asked to complete up to eighteen monthly questionnaires distributed from the Dartmouth Project Coordination Center to collect information related to colds, flu and flu-like illnesses.

Dartmouth Project Coordination Center staff will communicate directly with subjects via phone or e-mail for reminders or clarifications about questionnaires. Local study coordinators may be asked to assist in calling or e-mailing to remind subjects who have not returned questionnaires or have been ambiguous about participation, and may discuss the subject’s Colds and Flu Questionnaire participation at their regular study Interval phone calls.

The Introductory mailing will include:

- COLDS AND FLU QUESTIONNAIRES (Part 1: Daily Health Diary; Part 2: Questionnaire) with prepaid return envelopes
- Invitation Letter
- Information Sheet
- Opt-Out Postcard
- Participant Instructions
- Daily Health Diary Example
- Study logo refrigerator magnet
- $5.00 (in cash or gift card) for reviewing materials and considering participation

The subject may choose to complete the questionnaire(s) (implied consent) or may opt-out via postcard. Alternately, per local procedures, the subject may be consented in person, by mail or by phone to participate in the new questionnaires. Data will be collected regarding daily cold and flu symptoms, vaccinations, relevant diagnoses, medications prescribed or supplements taken for symptoms reported, and information about travel. The paper questionnaires will be returned by mail (using the prepaid envelopes provided) to the Dartmouth Project Coordination Center for data entry. Alternately, the subject may choose to complete the questionnaires by direct web entry.

Subsequent mailings.

Upon receipt of questionnaire(s) from the introductory mailing, subsequent mailings of up to three questionnaires will be mailed to the subject (or will be available on-line) until up to eighteen questionnaires have been distributed. The subsequent mailings will include:

- Subsequent mailing cover letter
- COLDS AND FLU QUESTIONNAIRE(S) with prepaid return envelope(s)
- $5.00 (in cash or gift card) to reimburse subject for each completed questionnaire returned at the time of the subsequent mailing
- Instructions for completing web-based questionnaires

Subjects who did not participate when only paper questionnaires were available will be given the opportunity to participate in the on-line version by opting in via postcard, phone call or e-mail to the Dartmouth Project Coordination Center.

Internet option mailing.

A mailing will be sent to announce the availability of the web-based Questionnaire to all subjects who did not participate when only the paper questionnaire was available. This mailing will include:

- Cover letter
- Opt-In Postcard for subjects who did not participate when only the paper questionnaire was available.
- Instructions for completing web-based questionnaires

Reminders.

A reminder letter, e-mail or phone call will be generated from the Dartmouth Project Coordination Center or local study coordinator if an expected paper questionnaire is not returned or an online questionnaire not submitted.

Final mailing

We will continue to collect up to five additional questionnaires (not to exceed a total of eighteen) after the completion of the treatment phase because of the long half-life of vitamin D. Upon receipt of the final questionnaires, a letter will be sent with any remaining gift cards for questionnaires completed and submitted.

- 1. *Schedule of Lab Measurement*

Laboratory measurements at years 1, 3 and 5 will be used for safety and compliance purposes. The schedule of laboratory tests are summarized in the following table for subjects with either 3 or 5 year follow-up colonoscopies.

| Lab Measurement | Entry | Year 1 | Year 3 | Year 5 |
| --- | --- | --- | --- | --- |
| Calcium | XO | XO | O | - |
| Creatinine | XO | XO | O | - |
| 25-(OH)-vitamin D | XO | XO | XO | O |

Key: Subjects scheduled for colonoscopy follow-up exam at 3 years: X

Subjects scheduled for colonoscopy follow-up exam at 5 years: O

The interim blood draw(s) will be performed 1 year after randomization, as well as 3 years after randomization for subjects with a 5-year surveillance cycle. The interim blood draw(s) will be collected within 30 days prior to and 90 days after the randomization anniversary date. If the total calcium measurement is slightly elevated or slightly low, a re-test (of the original blood draw specimen) or a re-draw (draw for a new blood specimen), including an albumin measurement, will be used to estimate the ionized calcium level.

The final blood draw (year 3 or year 5) will be within the 60 days prior to the follow-up colonoscopy, while the subject is still taking the study pills, and, preferably, should not be performed at the colonoscopy exam (e.g. after the colonoscopy preparation has been administered to the subject).

As already covered in the informed consent form, additional blood draw(s) will be performed in the event re-testing is required in follow up to lab results or in circumstances of overdue colonoscopy when the subject wishes to continue study treatment.

If the 25-(OH)-vitamin D value is out-of-range (less than 10 or more than 100 ng/ml) then a sample from the same blood draw will be re-tested at a clinical laboratory that is certified per CLIA (Clinical Laboratory Improvement Amendments). If the re-test value is less than 10 or more than 100 ng/ml then the subject and their health care provider will be notified of the result.

6.3.1 In follow up to an Unanticipated Problem Report of October 10, 2011:

Additional study blood draws and tests will be completed so that results can be utilized in ongoing data analyses and subject notification via participant materials for this purpose.

1. Subjects still on study treatment will have annual blood draws for creatinine measurement. Those whose levels are greater than the upper limit of normal will not continue study calcium treatment. Calcium levels will also be measured at these blood draws, as funding permits. As a result of these new procedures, the window for the “End of Treatment visit” and blood draw will be expanded to within approximately one year of the anticipated follow-up colonoscopy exam. Subjects will also be asked a new question regarding their last use of study pills or calcium supplements at this visit.
2. Subjects who have already completed the treatment phase of the study will have creatinine measured on stored blood specimens drawn at the “End of Treatment Visit.” Calcium, and other analytes related to kidney function, will also be measured on these stored samples, as funding permits.
3. Subjects who have completed the treatment phase of the study, and whose last study creatinine levels are above the normal lab range will be invited to return for a blood draw to re-measure the levels. Calcium levels will also be measured at these blood draws, as funding permits. In addition, these subjects will be asked to complete a health update using the current Observational Follow Up Questionnaire and will also be asked a new question regarding their last use of calcium supplements. A separate new Informed Consent form will be used to obtain subject consent for this additional study visit (the “Post-End of Treatment Additional Study Visit Consent”).
4. Subjects will be sent materials describing this new development. All subjects who were randomized into the study will be sent these materials with the sole exception of deceased subjects. The materials include:

a. Letter to Subject with Normal Creatinine Result

b. Letter to Subject with Above Normal Creatinine Result

c. Above normal creatinine result with specific result and lab range

d. Significant New Information/Findings sheet for Pre-EOT subjects

e. Significant New Information/Findings sheet for Post-EOT subjects

f. Post-EOT Additional Study Visit Consent

1. Subjects who choose to discontinue study calcium treatment will be urged to continue study vitamin D treatment.

6.3.2 April 11, 2012 modification to the creatinine criteria and procedures to be used due to the Unanticipated Problem

1. Because many of the local laboratories used for creatinine measurements in the study do not take sex, age or race into account, the creatinine reference ranges provided by those laboratories may not be appropriate for the individual subject. Therefore, the criteria for stopping study calcium treatment (randomized or two-arm) has been changed to an increase of 0.3 mg/dL or more from baseline (update to #1 in section 6.3.1 above, originally implemented in October 2011).
2. Similarly, subjects who complete or who already have completed the treatment phase of the study and whose last creatinine level in the study was increased by 0.3 mg/dL or more from baseline will be invited to return for a blood draw to re-measure the levels (update to #3 in section 6.3.1 above, originally implemented in October 2011).
3. In place of annual study blood draws during the treatment phase or a study visit after completion of the treatment phase, creatinine (and calcium) levels may be documented from medical records.

*6.4 Colonoscopy*

Colonoscopic visualization of the entire large bowel will be performed in each subject approximately 36 months or 60 months after the qualifying colonoscopy, as set out before randomization by each patient's clinician. This time frame (3 or 5 years) for colonoscopy surveillance following the qualifying examination is consistent with current clinical guidelines. Additional exams will not be encouraged unless required clinically; patients in whom more frequent exams are anticipated will not be randomized. Study coordinators will contact subjects by telephone or mail to remind them of their anticipated examination and to assist in scheduling, as possible. Study coordinators will contact subjects who do not return for examinations and attempt to persuade them to have their follow-up colonoscopy or determine why this is not possible. The specific timing of this follow up colonoscopy, which results in the end of the treatment phase of the study for this subject, will vary according to the circumstances of the individual subject and the scheduling procedures of the clinic and the patient’s clinician. Therefore, the window for this colonoscopy will be from 6 months prior to the 3 or 5 year anniversary of the qualifying colonoscopy exam and up to the End of Treatment Phase as specified in 6.8 below . Colonoscopies performed prior to this window may be considered interim exams, and study treatment may continue.

*6.5 RAND SF-36v2 Health Survey*

In addition to at enrollment, subjects will also be asked to complete an SF-36v2 health survey two additional times during the study--at the end of year 2 and at the time of the three-year or five-year follow-up colonoscopy exam. This 36-item self-administered questionnaire was constructed to survey health status in the Medical Outcomes Study. It was designed for use in clinical practice and research and has been extensively tested and validated. It assesses eight different health concepts: 1) limitations in physical activities because of health problems; 2) limitations in social activities because of physical or emotional problems; 3) limitations in usual role activities because of physical health problems; 4) limitations in usual role activities because of emotional problems; 5) general mental health (psychological distress and well-being); 6) bodily pain; 7) vitality (energy and fatigue); and 8) general health perceptions.

*6.6 End of Treatment Visit*

An End of Treatment Visit will be conducted within the 60 days prior to the follow-up colonoscopy. Study coordinators will contact subjects by phone or mail to schedule this visit. Prior to the scheduled visit, a packet (#4) will be sent to the subject with the following items:

- Letter confirming the appointment date and time
- Checklist for the appointment
- RAND SF-36 Health Survey
- Copy of the Observational Follow Up Informed Consent document

The End of Treatment Visit will include a final blood draw (see section 6.3 above), collection of the SF-36 Health Survey, and measurement of height and weight. In addition, the Observational Follow Up phase of the study will be described (see section 6.7 below). If the subject is interested in participating in the Observational Follow Up, then the informed consent process will be carried out and consent will be documented by the subject’s signature on the informed consent form.

*6.7End of Treatment Questionnaire*

At the end of treatment, all subjects will complete a final questionnaire that captures the same information collected on the Interval Questionnaire (symptoms, illnesses, doctor’s visits, hospitalizations, medications, over-the-counter preparations, dietary intake of calcium and vitamin D, and compliance with study tablets) as well as some additional information (exercise, smoking, bowel movements, and postmenopausal status). In addition, the name of a contact person will be collected to assist, if necessary, in locating a subject who can not be found at a later date. The End of Treatment Questionnaire will be collected within 60 days of the follow up colonoscopy that results in the end of treatment for that subject.

*6.8 End of the Treatment Phase*

The Treatment Phase of the study will end on August 31, 2013, after which subjects who have not already completed the Treatment Phase will be instructed to discontinue pill taking.

After the Treatment Phase ends, a packet will be mailed to subjects, including the following items:

- Treatment Assignment Notification from the Project Coordination Center at Dartmouth
- Participant Survey with self-addressed and stamped envelope for returning the completed survey to the Project Coordination Center at Dartmouth

When the main study Treatment Phase findings are first presented at a national meeting or published, all subjects will be notified of the findings by letter.

*6.9 Observational Follow Up*

Subjects will be invited to participate in an optional Observational Follow Up phase of the study that will begin following the end of treatment. In this phase of the study, subjects will continue to be followed on an observational basis (no study treatment) with annual questionnaires until the time of a subsequent colonoscopy that is at least three years from the follow up colonoscopy at which study treatment was ended.

Annual questionnaires will be used to collect updated information on the subject’s health, medical care, use of medications or supplements, and diet. These questionnaires will be collected within 30 days before or after the annual anniversary date of the end of treatment colonoscopy. The questionnaires may be collected by coordinator interview of the subject (preferred), or by subject self-completion of a paper form or a web-based form (once that technology is available in the study). Medical records will be obtained to confirm any medical events reported by the subject.

For any colonoscopies that are reported by the subject, medical records will be obtained and any pathology specimens will be obtained and reviewed by the Study Pathologist as described below (section 8.0) for pathology collected during the treatment phase of the study. Observational follow up will continue until a colonoscopy is performed that is at least 3 years from the end of treatment for that subject. Within 60 days of that colonoscopy, a final questionnaire will be collected and the subject’s participation in the Observational Follow Up phase of the study will end. However, if a colonoscopy is not performed by December 31, 2017, which is the end of the study, then observational follow up will end at that time.

***7.0 Clinical Procedures – Endoscopy***

The endpoint of the study will be new adenomas detected on follow-up colonoscopy. Colonoscopic visualization of the entire large bowel will be performed in each subject approximately 3 or 5 years after the qualifying colonoscopy, as set out before randomization by each patient's clinician.

*7.1 Overview of Procedure*

The coordinator at each Study Center will provide study colonoscopy guidelines to the endoscopist prior to each study exam. Participants will undergo pre-colonoscopy bowel preparation using a standard oral lavage or phospha-soda bowel cleansing preparation, according to the preference of the local study colonoscopist. On the day of the examination each subject will be asked to sign a routine informed consent document for the procedure as required by each institution. Prior to colonoscopy, each subject may receive intravenous medication to achieve desired conscious sedation following the usual practice of the endoscopist performing the procedure. Continuous monitoring of the patient will be carried out by the endoscopy unit nursing personnel before, during, and after the procedure until adequate recovery, according to the standards and practice protocols of the endoscopy unit.

Colonoscopy to the cecum will be performed using standard techniques. The entire colonic mucosa will be carefully examined during withdrawal of the instrument. Detected polypoid lesions will be sized in comparison to open biopsy forceps and then resected using standard biopsy forceps or snare techniques, avoiding cautery if possible. The location of each lesion will be recorded by segment of the colon and centimeters from the anus, and will be submitted to the local pathology laboratory in a separate specimen bottle. Colonoscopic findings will be recorded on the colonoscopy/surgery reporting form immediately after the procedure. If at colonoscopy the preparation is judged to be inadequate, or if the colonoscopy is incomplete for other reasons, a second colonoscopy should immediately be scheduled.

The endoscopist will inform each subject of findings at the time of the procedure. If polyps or masses are found, the participant will be contacted by an appropriate clinician with the pathology findings as soon as they are available, and any required follow-up treatment will be arranged. Any other follow-up required by a participant, including care of complications, will be handled at each Study Center by the endoscopist and/or other medical staff according to the usual practice of the endoscopy unit.

# *7.2 Quality Assurance*

Endoscopy guidelines in the form of laminated pocket-sized cards for physicians will be provided by the Dartmouth Project Coordination Center. The card will be personalized for each center, giving the study coordinator’s phone number so that physicians can call them with any questions or problems. Current Polyp Prevention Study guidelines for endoscopists include the following:

- Notation of the adequacy of the bowel prep
- Verification of the completeness of colonoscopy by documentation of at least 2 usual landmarks, e.g., cecal strap, appendiceal orifice, ileocecal valve.
- Biopsy of all mucosal excrescences and lesions suspicious for neoplasia. Avoidance of fulguration without biopsy.
- If clusters of diminutive (<5mm) polyps are noted in the distal rectum, removal of all of the polyps or a representative sample (to a maximum of 5) and documentation as accurately as possible of the number of any remaining polyps (a range of estimated numbers if necessary).
- Placement of each lesion specimen in a separate pathology bottle.

***8.0 Endpoint Determinations and Pathology***

Colorectal adenomas are the study’s main endpoint, so they will be ascertained completely and accurately. At all endoscopic examinations after randomization, all raised mucosal lesions, and all flat lesions suspicious for neoplasia, will be biopsied and excised, their location and size noted, and pathology material reviewed by the study pathology center for confirmation of diagnosis. If clinical events lead to interim endoscopy, all the relevant information and histological material will be obtained from that endoscopy as well. For each subject, the number of mucosal lesions will be tabulated, along with their size, histological characteristics, and location in the bowel.

Tissue biopsies of colon and rectal polyps will be collected using standard procedures during endoscopies performed by qualified endoscopist at each of the Study Centers. If at all possible, each polyp specimen will be put into a separate pathology bottle for processing. Slides will be prepared at the local Study Center pathology labs according to standard procedures. Study coordinators will purchase or request loans of these slides from the pathology labs for analysis by our study pathologist. These slides will be shipped to the Dartmouth Project Coordination Center on a quarterly basis. Slides will be stored at room temperature in a secure (locked) office at Dartmouth prior to batch shipments to the study pathologist (Dr. Dale Snover at Fairview Southdale Hospital in Edina, Minnesota). After review by the study pathologist, the slides will be returned to the Dartmouth Project Coordination Center and, if on loan, then returned to the Study Center of origin, unless the subject consents to their storage for future research and permission is obtained from the pathology laboratory of origin. All pathology slides that are collected during this polyp prevention trial will be tracked electronically from the time of shipment to the Project Coordination Center to the time of return to the Study Center of origin. The tracking number used will be the local pathology ID number that is found on the slide. An archive of slides purchased by the study will be maintained at the Project Coordination Center as part of the study records.

Digital microscopy is increasingly being used for histology specimens. Thus, whenever possible, digital images will be made available to the Study Pathologist instead of the slides themselves. Depending on the availability of the requisite scanning equipment, the digital images may originate at the Study Centers, or may be created at the Dartmouth Project Coordination Center (after having obtained the slides from the Centers without this equipment). The images will be made available to the Study Pathologist via the internet, or via mailed DVD's. An archive of the images will be maintained at the Project Coordinating Center as part of the study records.

The study pathologist will review all pathology specimens (slides or digital images) without knowledge of treatment assignment. Each polyp will be classified (using standard criteria) as neoplastic (tubular, tubulovillous, villous, or serrated adenomas or cancer), hyperplastic, hamartomatous, retention, lymphoid, or other. In addition, for the neoplastic polyps, a statement with regard to the degree of atypia present will be made using standard criteria. When there is disagreement between the local and study readings, we will first ascertain that the study pathologist has read the particular specimen (slide or digital image) used to make the local diagnosis and will send further specimens if appropriate. If disagreement remains, we will accept the study pathologist's diagnosis.

***9.0 Laboratory Evaluations – Blood Samples***

*9.1 Laboratories*

Creatinine, total calcium, and albumin measurements will be performed at local clinical laboratories associated with each of the participating Study Centers or their recruitment sites.

Serum vitamin D measurement will be conducted at the University of California - Los Angeles (UCLA) Nutrition Research Center Laboratory by Dr. David Heber. This is a research laboratory that is not certified per CLIA (Clinical Lab Improvement Amendments). Therefore, the results of these measurements may not be used in clinical decision making and will not be disclosed to subjects or to their health care provider. However, if the 25-(OH) vitamin D value at an interim or final study blood draws is out of range (below 10 or more than 100 ng/ml) then the blood sample will be re-tested at a CLIA certified laboratory. Mayo Medical Laboratories (Rochester, MN) is being used for this purpose.

DNA extraction from buffy coat samples and VDR genotyping will be conducted in Dr. Robert Haile’s laboratory at the University of Southern California in Los Angeles or in another qualified collaborating or commercial laboratory.

Blood samples that will be used for DNA isolation and for future research will be stored at the Norris Cotton Cancer Center’s (NCCC) Clinical & Molecular Pharmacology Shared Resource Laboratory in Lebanon, New Hampshire.

*9.2 Collection and Handling Procedures*

Whenever possible, subjects should be fasting prior to all study blood draws (e.g. no food or beverage intake other than water for 8 hours prior to the blood draw). Regardless, the time of last food or beverage consumption will be collected. Customized prepackaged bar-coded kits will be provided containing materials for collecting, processing, and aliquoting the blood samples. Blood samples will be collected according to standard operating procedures at each Study Center and sent to local labs for analysis or promptly frozen at -20°C or below pending transfer to a -70°C freezer for storage until shipment to the UCLA Nutrition Research Center Laboratory or the NCCC Clinical & Molecular Pharmacology Shared Resource Laboratory. Each individual blood specimen will be bar-coded and tracked electronically from the time of collection at the Study Center. Biospecimen transport will adhere to stringent shipping standards. The Study Centers, study laboratories (at UCLA and USC), and the biospecimen storage facility at the NCCC will be equipped with at least one bar code reader for logging and tracking specimens.

As necessary for all study blood draws, a re-draw may be required to collect blood if the original specimen is lost, destroyed, damaged, or if a re-analyses is required (e.g., albumin measurement to estimate ionized calcium at Year 1 or Year 3 safety assessment).

*9.3 Methods for Laboratory Procedures*

*Measurement of Calcium and Creatinine*

Blood levels of creatinine, total calcium, and albumin will be measured according to standard procedures at the local clinical laboratories. The normal reference ranges of the local laboratories will be used for eligibility assessment at enrollment and safety assessment at year 1 and, where applicable, year 3 (for those with a 5 year follow-up colonoscopy interval).

*Measurement of Vitamin D*

25-Hydroxy-Vitamin D Determination:

The Gamma-B 25-hydroxy-vitamin D RIA kit from IDS Inc. (Fountain Hills, AZ) is used to determine serum concentrations of 25-hydroxy-vitamin D. The IDS gamma-B 25-OH-vitamin D kit is a liquid phase radioimmunoassay. The addition of reagent 1 and 2 causes the precipitation of serum proteins and 25-hydroxy-vitamin D remains in solution. Following centrifugation, portions of the supernatant are incubated with ^125^I labeled 25-hydroxy-vitamin D and a highly-specific sheep antibody to 25-hydroxy-vitamin D. Separation of antibody-bound tracer from free is achieved by a short incubation with Sac-Cel (anti-sheep IgG cellulose) followed by centrifugation and decanting. Bound radioactivity in the Sac-Cel pellet is inversely proportional to the concentration of 25-hydroxy-vitamin D. The percent binding is determined for several standard concentrations and a calibration curve is established. Percent binding of the unknown samples is calculated and concentration determined from the calibration curve. For quality control purposes, two kit controls are analyzed with every batch. In addition five unknown samples from the international Vitamin D External Quality Assessment Scheme (DEQAS) program are analyzed with every kit. The intra assay correlation coefficient is 5.3%-6.1% (26.5-151 nmol/L) and inter assay correlation coefficient is 8.2-7.3% (19.6-136 nmol/L).

*Measurement of VDR polymorphisms*

Very briefly, DNA will be extracted and VDR genotyping will be conducted by PCR analysis using gene-specific primers.

***10.0*** ***Ancillary Studies***

*10.1 The Dartmouth Polyp Prevention Biospecimen Repository*

All subjects will be invited to contribute blood samples, and extracted DNA, as well as colorectal tissue specimens in the parent study, and biospecimens collected in added ancillary studies, for future research on colorectal neoplasia or other disorders common in the study population. Their decision will be documented as part of the informed consent.

In the parent study, for subjects who agree to future research on their blood samples, approximately 12 additional 1.0 or 0.5 ml aliquots of plasma or serum will be obtained at each blood draw and shipped to the biospecimen storage facility at the NCCC Clinical & Molecular Pharmacology Shared Resource Laboratory. In addition, DNA that is extracted from these subject’s blood samples, and that is not used up during the VDR genotyping, will be stored for future research.

In the parent study, for subjects who agree to future research on the tissue samples collected during routine clinical care, tissue blocks will be requested from the appropriate pathology laboratories and shipped to the biospecimen storage facility at the NCCC Clinical & Molecular Pharmacology Shared Resource Laboratory. If the pathology laboratory requests return of the tissue blocks, they will be used to obtain sections or cores for slides or Tissue Micro Arrays. Tissue blocks, sections and cores will be stored at the NCCC Clinical & Molecular Pharmacology Shared Resource Laboratory for future research.

In ancillary studies, unless otherwise provided in the protocol and consents, all biospecimens collected will be shipped to the biospecimen storage facility at the NCCC Clinical & Molecular Pharmacy prior to shipment to collaborating labs. Any biospecimen that is not used up in lab analyses will be stored for future research at the NCCC Clinical & Molecular Pharmacology Shared Resource Laboratory.

All future research utilizing these biospecimens will require IRB review and will be consistent with the intent of the informed consent obtained from the subject.

*10.2 Biospecimen Analyses*

*10.2.1Genetic variant influencing response to vitamin D and calcium*

Polymorphisms in the vitamin D receptor (VDR) will be genotyped as part of the primary protocol for all randomized subjects. In this ancillary aim, we will investigate the association of common genetic variation in other genes involved in calcium or vitamin D metabolism or signaling with the response to vitamin D supplementation (increase in serum 25-hyrdoxy-vitamin D) and the occurrence of colorectal adenomas. Genes to be investigated are likely to include, but are not limited to, the vitamin D binding protein (DBP), retinoid X receptor (RXR), vitamin D 25-α-hydroxylase (CYP2R1), vitamin D 1-α-hydroxylase (CYP27B1), vitamin D 24-α-hydroxylase (CYP24A1), 7-dehydrocholesterol reductase (DHCR7), the calcium-sensing receptor (CaSR), the apical sodium-dependent bile acid transporter (ASBT) and the apical calcium channel (TRPV6). There is no separate consent for this ancillary aim. Instead, only subjects that authorized storage of their blood specimens for future research, including genetic analyses, will be included in this analysis. Notably, these metabolic genes are thought to be low penetrance with only modest effects on colorectal carcinogenesis. Confidentiality is protected in the following ways: 1- DNA samples will be de-identified and labeled only with unique barcode numbers, 2- a de-identified “Limited Data Set”, as defined by HIPAA, will be used to examine the results of these genetic analyses. In addition, as described in section 14.7 below, we have a obtained a Certificate of Confidentiality for this study, as issued by the National Institutes of Health, that protects subjects from the release of any potentially sensitive genetic information to persons not associated with this research.

10.2.2 Early detection of colorectal neoplasia with circulating tumor DNA (ctDNA)

The main objective of these analyses is to develop clinical tests that can be used to identify the presence of colorectal neoplasms at a stage when the tumors can be cured by surgery. State-of-the-art technologies will be used to test for the presence of mutant DNA template molecules in plasma specimens that have been coordinated with tissue specimens collected at the targeted colonoscopy. Paired samples of tissue and plasma collected prior to the development of clinically significant colorectal tumors will allow analyses of a critical aspect of the entire approach: that circulating tumor DNA can be detected prior to any symptoms, well before they were discovered through conventional methods. The criteria for choosing paired specimens will be based on the size of the colonoscopy lesion, the pathology diagnosis, and the timing of study blood collected prior to the colonoscopy at which the lesion of interest or cancer was removed, together with subject consent for future research on blood and tissue. Targeted tissue blocks obtained from local pathology labs will be shipped from local study centers to the Dartmouth coordination center for administrative processing before shipping to the lab performing the analyses. Concurrently, targeted plasma specimens stored at the NCCC Clinical & Molecular Pharmacology Shared Resource Laboratory will be processed for shipping to the lab. All tissue specimens will be returned to the Dartmouth coordination center for administrative processing before shipping back to the local study center/pathology lab, if requested. Blood samples will likely be exhausted in the analyses, but any remaining samples will be returned to the NCCC Clinical & Molecular Pharmacology Shared Resource Laboratory.

10.2.3 Metabolomic Study of the Effects of Vitamin D and Calcium Supplementation

The objective of this ancillary study is to use a global metabolomics approach to better understand the independent and synergistic antineoplastic actions and other effects of vitamin D and calcium in humans.  The aims are to elucidate 1) the individual and combined effects of vitamin D and calcium on metabolomic pathways in humans (thus providing a more comprehensive assessment of the benefits and risks of these heavily promoted agents as supplements), 2) whether these metabolomic changes correlate with genetic variation and systemic and tissue-specific biomarkers in humans (and thus their direct relevance to the chemoprevention of colorectal neoplasms), and 3) whether the metabolomic changes predict the occurrence of sporadic colorectal adenomas in humans, thus paving the way for the development of treatable biomarkers of risk for colorectal neoplasia that are analogous to lipid profiles for the prevention of ischemic heart disease. In this pilot study we will investigate the effects of supplementation with vitamin D and/or calcium on individual metabolites and metabolic pathways in a sub-set of participants (n = 120) who were enrolled in the Calcium/Vitamin D, Biomarkers, and Colon Polyp Prevention ancillary study (see section 10.4 below.) There is no separate consent for this ancillary study; only subjects that authorized the storage of their blood specimens for future research will be included in this analysis. The aims will be accomplished by conducting state-of-the-art, high-resolution metabolomic profiling using liquid chromatography-Fourier transform mass spectrometry (LC-FTMS) analysis of blood plasma collected before and after supplementation with vitamin D3 and/or calcium. For this purpose blood samples will be shipped from the Dartmouth Biorepository to the collaborating laboratory at Emory University (Dr. Dean Jones), where the metabolomic analyses will be performed.

10.2.4 Vitamin D_3_, Calcium and Biomarkers of Inflammation and Gut Barrier Function

The objective of this ancillary study is to test a novel hypothesis that calcium and vitamin D supplementation strengthens the gut mucosal barrier as indicated by modulation of cell cycle, cell adhesion, receptors responsive to bacterial components, and markers of intestinal and systemic inflammation and colonic permeability in humans. The aims of the proposed study are 1) in biopsies of normal-appearing rectal mucosa, to obtain data on the separate and joint effects of calcium and vitamin D_3_ supplementation on biomarkers of gut barrier function (Ki-67/MIB-1, caspase-3, TLR4, TLR5, ZO-1, claudin-1, occludin, mucin-17, E-cadherin, and β-catenin) and inflammation (NFκB and TNFα); 2) in blood samples, to obtain data on the separate and joint effects of calcium and vitamin D supplementation on biomarkers of inflammation (GM-CSF, IFNγ, TNFα, IL-6, IL-1β, IL-2, IL-4, IL-8, IL-10, IL-12p40, IL-12p70 and IL-17) and gut barrier function (specific IgG and IgA to lipopolysaccharide [LPS] and flagellin, LPS binding protein [LBP], and intestinal fatty acid binding protein [I-FABP]); 3) to investigate whether the findings for the tissue-specific biomarkers are correlated with circulating biomarkers of gut barrier function and inflammation; and 4) to obtain preliminary data on whether changes in the biomarker levels are associated with decreased sporadic colorectal adenoma recurrence. The proposed scope of work is limited to laboratory and statistical analyses using already collected biological samples and questionnaire data. In this adjunct study, the blood and tissue assays will be performed in a sub-set of participants (approximately 460) who were enrolled in the Calcium/Vitamin D, Biomarkers, and Colon Polyp Prevention ancillary study (see section 10.4 below). There is no separate consent for this ancillary study; only subjects who authorized the storage of their blood and tissue specimens for future research will be included in this analysis. The aims will be accomplished by conducting immunoassays to measure biomarkers in tissue or blood plasma/serum samples collected before and after supplementation with vitamin D_3_ and/or calcium. The blood samples are stored at the biospecimen storage facility at the NCCC Clinical & Molecular Pharmacology Shared Resource Laboratory and tissue samples are stored at Dr. Bostick’s Molecular Epidemiology and Biomarkers Research Laboratory, Emory University.

10.3 *Data Analyses*

Data is collected and stored at Geisel School of Medicine at Dartmouth. Per federal regulations, research using de-identified data does not involve "human subjects" and does not require IRB review. Thus, de-identified data from this data set may be sent outside of Geisel School of Medicine at Dartmouth for new analyses that are not specified in this protocol without additional IRB review.

10.4 *Calcium/Vitamin D, Biomarkers, and Colon Polyp Prevention (PPS4B)*

This “biomarker study” is funded the National Cancer Institute and the Franklin Foundation through Emory University and the Polyp Prevention Study Group consortium as an adjunct study to the Vitamin D/Calcium Polyp Prevention Study (parent study). Subjects in the parent study will be invited to participate in this adjunct study and, if they agree, will be consented separately to this study.

*10.4.1 Objectives*

The primary aims are to investigate the effect of calcium and/or vitamin D on the individual components and aggregate profile of a molecular phenotype panel of biomarkers of risk for colorectal cancer, and to assess whether this modulation is associated with decreased recurrence of sporadic colorectal adenomatous polyps. The following biomarkers will be measured in biopsies of normal-appearing colorectal mucosa:

- inflammation (COX-2 expression)
- early colon carcinogenesis pathway genes (APC pathway: expression of APC, β-catenin, E-cadherin; mismatch repair pathway: expression of MLH1, bax)
- cell cycle events (long-term proliferation: expression of telomerase; apoptosis inhibition and promotion: expression of bcl-2, bax)
- autocrine/paracrine growth promotion and inhibition factors (expression of TGFα, TGFβ_1_)

The secondary aims are as follows:

- To clarify whether the differences in the biomarkers among the treatment groups at the 3- or 5-year colonoscopies precede and may predict adenoma recurrence, “non-prep” rectal biopsies will be taken at baseline and at a 1-year follow-up visit.
- To clarify whether the differences in the biomarkers in rectal mucosa among the treatment groups at the 3- or 5-year colonoscopies also occur more proximally in the colon, at the 3- or 5-year colonoscopy—in addition to the rectal biopsies—biopsies will also be taken from the mid-sigmoid and proximal ascending colon.
- To clarify **a)** whether the differences in the biomarkers in rectal mucosa among the treatment groups at the 3- or 5-year colonoscopies are affected by colonoscopic preparation, and **b)** whether changes in biomarkers from baseline to one year are maintained, accumulative, or attenuated by 3 or 5 years, non-prep rectal biopsies will be taken 7 – 21 days prior to their colonoscopies with biopsies.
- Estimate the normal variability of the biomarker panel in sporadic adenoma patients over time. This will be done by estimating the inter- and intra-subject components of variability over the 3 or 5 years.
- Investigate whether biomarker responses to treatments vary according to nonsteroidal anti-inflammatory drug (NSAID) use or vitamin D receptor (VDR) genotype.

*10.4.2 Recruitment and Enrollment Procedures*

The parent study defines the pool of potential subjects for the biomarker study. Two *additional* exclusion criteria for this adjunct study are: a) unable to be off aspirin for 7 days, and b) a history of bleeding disorder or current use of anticoagulant medication. Subjects will be invited to participate in the Biomarker Study in one of two ways:

1. Rectal biopsies at 3- or 5-year follow-up colonoscopy only: Randomized subjects from all Study Centers participating in the parent study will be invited to participate in rectal biopsies that occur at their 3- or 5-year follow-up colonoscopy. Of the approximately 2,400 randomized participants in the parent study, we expect to recruit approximately 1,328 to participate in this portion of the biomarker study.

Study coordinators will contact subjects by mail or telephone several months prior to their scheduled 3- or 5-year follow-up colonoscopy to describe the biomarker study and to invite the subject to participate. Interested subjects will be consented to the biomarker study at an in-person visit prior to their 3- or 5-year follow-up colonoscopy.

1. Additional Biopsies: Subjects from the South Carolina and Emory Study Centers only who have been enrolled into the parent study, but not yet randomized, will be invited to participate in the following additional biopsies:
   1. Biopsies at outpatient sigmoidoscopy: rectal biopsies will be collected during outpatient sigmoidoscopies occurring at randomization, at the 1-year follow-up visit, and at a visit 7 – 21 days before the 3- or 5-year follow-up colonoscopy
   2. Additional biopsies at follow-up colonoscopy: biopsies will be taken from the mid-sigmoid and proximal ascending colon at the 3- or 5-year follow-up colonoscopy (in addition to the rectal biopsies collected at this follow-up colonoscopy).

We expect to recruit approximately 200 subjects to participate in this portion of the biomarker study. Study coordinators will contact subjects by telephone prior to their deadline for randomization in the parent study to describe the Biomarker Study and to invite the subject to participate. Interested subjects will be consented to the Biomarker study at an in-person visit at the time of their randomization into the parent study.

*10.4.3 Biopsy Collection and Analysis*

All colorectal biopsies will be obtained using currently successful protocols in the procedure rooms used by the participating gastroenterology offices by a physician trained in the procedure, with the assistance of the local Study Coordinator. For randomized participants from all Study Centers who consent to this adjunct study, rectal biopsies only will be obtained only during the patient’s follow-up colonoscopy. For enrolled participants from South Carolina and Emory who consent to the additional biopsies, outpatient rectal biopsies will be obtained via sigmoidoscopy at randomization, one-year follow-up, and 7 – 21 days prior to their clinical colonoscopy; also, in addition to rectal biopsies, biopsies will be obtained from the mid-sigmoid and proximal ascending colon during the patient’s follow-up colonoscopy.

Prior to the colonoscopies, participants will use a PEG (polyethylene glycol based) preparation in order to clear out the bowel. This specific type of preparation is used because it does not interfere with the biomarker measurements that will be performed. The PEG preparation will be provided by the study if it is not available as a standard preparation at the local gastroenterology clinic. No bowel-cleansing preparation is used for the outpatient rectal biopsies.

At least six adequate rectal biopsies must be obtained during the colonoscopy (from all participants) or sigmoidoscopy (from participants consenting to the additional biopsies). In addition, at least three adequate colonic biopsies must be obtained from each of the mid-sigmoid and proximal ascending colon sites during the follow-up colonoscopy for participants consenting to the additional biopsies. If a biopsy pinch is inadequate, another will be obtained. Biopsy specimens are immediately examined and processed for storage in formalin. All biopsy samples will be sent to Dr. Roberd Bostick’s laboratory at Emory University for analysis. The biomarkers will be measured by immunohistochemical methods followed by quantitative image analysis of the total expression and distribution of the expression of the biomarkers in colon crypts.

*10.4.4 Sample Size*

Adenoma recurrence in the parent study is expected to be similar to that found in the Calcium Polyp Prevention Study, in which, among the 832 subjects who completed the study, 52% of the placebo and 45% of the calcium group had a recurrence of ≥ 1 adenoma, for a statistically significant relative reduction in adenoma recurrence of 15% (adjusted RR = 0.85; 95% CI 0.74 – 0.98). We expect to recruit 1,328 to participate in the rectal biopsies at colonoscopy (from all study centers) and we expect 200 of these subjects (from Emory and South Carolina) to participate in the additional biopsies as well.

Primary Aims: For the estimated power for Primary Aim 1a, detection of an effect of treatment on the biomarker level, we assumed testing will be performed using an F-test based on a general linear model for each treatment separately, with 2-sided testing at α = 0.05, and that the biomarkers are log normally distributed. For each of the biomarkers we will have greater than 80% power for testing each of the hypotheses described in the specific aims: ie, calcium and/or vitamin D will change the total expression of each of the individual biomarkers, and these changes will be associated with decreased adenoma recurrence. For most of the biomarkers, we will have over 80% power to detect a treatment effect even if the effect is only half as large as differences observed in a previous case-control study. In addition, the power to detect a calcium-vitamin D interaction for effect sizes (in SDs) of 0.08, 0.12, 0.16, 0.24, and 0.24 is 24%, 47%, 71%, 88%, and 96%, respectively.

For the estimated power for Primary Aim 1b, detection of an association between each biomarker level and recurrence of one or more adenomas, we estimated power by assuming testing will be performed using a logistic model, with 2-sided testing at α = 0.05, 50% polyp recurrence at the mean biomarker level, marker-covariate R^2^s = 0.2, and assuming that the marker is log normally distributed. For each of the biomarkers we will have > 80% power for testing most of the hypotheses described in the specific aims.

Secondary Aims: The sample size for the secondary aims (n = 200; 50/treatment group) is unlikely to provide sufficient power to allow definitive statistical testing of the hypotheses for these ancillary aims. However, the sample size will be sufficient for refining our biomarker profile and sample size estimates for the primary aims, which will mostly be addressed during a subsequent competitive grant renewal period. The sample size will also be sufficient for the other purpose of the first four secondary aims of the biomarker study, which is to provide enough supplemental, descriptive data to support a qualitative assessment of the quality and generalizability of the data and results obtained from the primary aims of the biomarker study. The data generated by addressing the secondary aims accomplishes this purpose by providing assurance that biomarker differences at follow-up colonoscopies in fact are changes that precede adenoma recurrence, areas of the colon proximal to the rectum are affected by treatment, and biomarker findings taken during colonoscopy reflect those that are taken without preceding colon preparation.

For Secondary Aim 1 we calculated the estimated power to detect an association between recurrence at 3 or 5 years and change in the biomarker from baseline to one year, adjusted for the baseline biomarker level. We assume 200 subjects (50/treatment group); that a logistic model will be used with recurrence (yes/no) at 3-5 years as the dependent variable; that the OR per unit increase in ∆M is 2.0 after adjustment for the baseline biomarker level (where ∆M is the change from baseline to one year); that the ∆M is correlated with the other independent variables, baseline biomarker level, treatment group, and time until the follow-up colonoscopy, with R^2^ = 0.2; and that, among those with average values of the independent variables, adenoma recurrence at 3-5 years is 50%. With these assumptions, the power to detect an adjusted OR of 0.5 or 2.0 is about 99% (at R^2^ = 0.5, the power is 0.93). For Secondary Aim 2, we calculated the estimated power to detect an effect of treatment on the biomarker level measured in the proximal colon, just as for the first primary aim, but for effect sizes that were larger. For effect sizes as large as 0.48 SDs, the power to detect such a difference would be about 92%.

*10.4.5 Additional Risks*

The additional physical risks associated with the adjunct study are those associated with the collection of the colorectal biopsies which are outside of the standard of care. The procurement of 1 mm thick colorectal mucosal biopsies is considered a relatively minor procedure with very low risk.

1) Biopsies at colonoscopy - On all participants at all clinical centers, small pinches of tissue (biopsies) 1 mm thick (less than 1/16 of an inch) will be taken from the lower colon, or rectum about 3 – 4 inches in from the anus on normal-appearing colorectal tissue. A total of 6 – 8 pinches of tissue will be taken. It is anticipated that these additional biopsies will add about 3 - 5 minutes to the colonoscopy time.

On all participants who agree to the additional biopsies (only at the South Carolina and Emory centers), 3 – 4 pinches of tissue will also be taken at the mid-sigmoid colon and at the proximal ascending colon, adding another 3 – 5 minutes to the colonoscopy time (ie, there will be a total of 12 – 16 biopsies from all three colon sites combined, adding about 6 – 10 minutes to the colonoscopy time).

The biopsies are a standard clinical diagnostic procedure with very little risk. Patients do not experience any pain from the biopsies themselves; the only discomfort will be that from the colonoscopy itself. Almost all patients experience some cramping during and after a colonoscopy; although unlikely, it is possible that the biopsies might mildly contribute to the cramping. A possible complication of the biopsies is minor bleeding. Other complications could be more serious bleeding, perforation of (that is, producing a hole in) the bowel wall, or infection, which could result in needing surgery. These complications are extremely rare, so rare that none have occurred in studies like this one involving over 12,000 persons so far.

2) Outpatient rectal biopsies at sigmoidoscopy – On all participants who agree to the additional biopsies (only at the South Carolina and Emory clinical centers), outpatient, non-prep rectal biopsies will be taken via sigmoidoscopy. These sigmoidoscopic biopsies involve inserting a tube—about as long and as big around as a doctor’s examining finger—through the anus into the rectum or lower colon to a depth of about 3-4 inches. At this spot, 4 - 6 tiny pinches of tissue one mm thick (less than 1/16 of an inch) will be taken. The procedure takes less than two minutes, is painless (the only discomfort is like that of having a rectal exam), and is very low risk—about like having blood drawn.

Protection against additional risk: All subjects are counseled to refrain from aspirin intake for 7 days prior to biopsy procedures to reduce the risk of bleeding. In addition, potential participants are screened for contraindications to colorectal biopsies (unable to be off aspirin for 7 days OR a history of bleeding disorder or current use of anticoagulant medication) and are not enrolled if these conditions exist. The Data and Safety Monitoring Committee for the parent trial will serve in this capacity for the adjunct study, by monitoring for potential biopsy complications and recommending whether the trial should continue.

*10.5 Vitamin D/Calcium Mammography Study*

The “Vitamin D/Calcium Mammography Study” is an ancillary study to the Vitamin D/Calcium Polyp Prevention Study (parent study) under the direction of Dr. Susan Gapstur at Northwestern University. Female subjects in the parent study will be invited to participate in this ancillary study and, if they agree, will be consented separately for this study.

*10.5.1 Background and rationale*

The radiographic appearance of the breast varies according to differences in the relative

distributions of fat and fibroglandular tissues. The extent of mammographically detected

fibroglandular breast tissue (i.e., percent breast density) is a strong breast cancer risk factor.

Although a large body of evidence shows consistent associations of percent breast density with age, reproductive factors, body mass index (BMI) and use of postmenopausal hormone replacement therapy (HRT), there are few data on associations of modifiable lifestyle factors with breast density. Results of some observational epidemiologic studies suggest that dietary vitamin D and calcium intake might be associated with a lower risk of breast cancer. In addition, other observational studies have shown inverse associations of vitamin D and calcium intake with percent breast density. It is possible that a protective effect of vitamin D on breast cancer etiology is mediated through vitamin D signaling effects on mammary epithelial cell differentiation, proliferation and apoptosis. Similarly, calcium also appears to exert anti-neoplastic effects.

The Vitamin D/Calcium Mammography Study is designed to assess the effects of supplemental vitamin D and calcium carbonate (and their synergistic effects) on mammographic breast density as an ancillary study of the Vitamin D/Calcium Polyp Prevention Trial. This multi-centered, randomized clinical trial provides an ideal opportunity to study the associations of vitamin D/calcium with breast density for several reasons. First, the primary exposures will be known quantities of vitamin D and calcium, thus removing the potential measurement errors introduced by dietary data collection. Second, we will be able to compare changes in density over time across the calcium only, vitamin D only, both or placebo groups. Third, the availability of high-quality data collected on potential confounding factors (e.g, hormone use, BMI, menopausal status, and age) will allow us to control for potential confounding factors that might differ between groups. Fourth, we are able to explore whether variants in genes in the vitamin D receptor might modify associations of supplemental vitamin D with percent breast density. If successful, the results of this study could be used to inform the design of a chemoprevention trial of vitamin D and/or calcium supplement to prevent breast cancer with percent breast density as the intermediate endpoint.

*10.5.2 Objectives*

The primary aim of this study is to assess the effects of supplemental vitamin D_3_ (1000 IU/daily) and calcium carbonate (1200 mg elemental calcium/day) on mammographic breast density.

Our primary hypotheses are:

1. Vitamin D_3_ (1000 IU/day) supplementation reduces mammographic breast density.

2. Calcium carbonate (1200 mg elemental calcium/day) supplementation reduces mammographic breast density.

Our secondary hypothesis is:

1. The combination of both vitamin D_3_ (1000 IU/day) and calcium carbonate (1200 mg elemental calcium/day) supplementation reduces mammographic breast density more than supplementation with calcium carbonate or vitamin D alone.

An exploratory hypothesis is:

1. The effect of supplementation with vitamin D_3_ on mammographic breast density will be modified by polymorphisms at the 3' end of the vitamin D receptor gene.

2. The combination of both vitamin D_3_ (1000 IU/day) and calcium carbonate (1200 mg elemental calcium/day) supplementation reduces mammographic breast density compared to placebo.

*10.5.3 Recruitment and Enrollment Procedures*

The parent study defines the pool of potential subjects for this study. Two *additional* exclusion criteria for this ancillary study are: a) a history of breast cancer, and b) not anticipating having a mammogram during the duration of the parent study. Subjects will be invited to participate in the Mammography Study in one of two ways:

1. Women currently being enrolled in the parent study will be invited to participate at the time of their in-person intake visit. Study coordinators will ask women to complete the mammography eligibility and intake questionnaire. Those who are eligible will be invited to participate in this ancillary study. If they are interested, the informed consent process will be carried out at this visit and documented on the informed consent form. The subject will also be asked to sign a medical release form to allow collection of their mammograms. Upon the subject’s randomization in the parent study, the study coordinator will collect the most recent screening mammogram from prior to enrollment, as well as subsequent mammograms from at least one year after enrollment into the parent study up until the end of the subject’s participation in the parent study. Subjects that consent to this ancillary study but become run-in failures in the parent study will be dropped from this study and their mammograms will not be collected.
2. Women already randomized into the parent study, will also be invited to participate. Study coordinators will ask women who are currently randomized in the study if they are interested in participating in the Mammography Study during an interim study visit (i.e. the year 1 blood draw), or during phone contact for an interval questionnaire. For interested and eligible subjects, local IRB procedures for obtaining informed consent will be followed. Where permissible, the informed consent process will be carried out over the phone and then documented by mailing an informed consent form and a release of medical information to the subject for their signature. Once the signed consent form is returned the study coordinators will collect the most recent screening mammogram from prior to enrollment, as well as subsequent mammograms from at least one year after enrollment in the parent study up until the end of the subject’s participation in the parent study.

*10.5.4 Collecting Mammogram Information*

Once a subject is enrolled in this ancillary study, the study coordinators will ask each woman a few questions regarding their screening mammography history, including information on where and when the most recent mammogram was conducted and whether she was taking medications containing female hormones, such as hormone replacement therapy or birth control, at the time the mammogram was performed. She will be asked to sign a release of medical information form to allow collection of one of the images taken at that mammogram. Similarly, at the time of the interval (semiannual) interview, women enrolled in this ancillary study will be asked if they had a recent screening mammogram and, if so, where and when that mammogram was performed.

At the time of a screening mammogram, four images are generally taken: craniocaudal and medio-lateral oblique images of each the right and left breast. For this study, we will request the left breast craniocaudal image if available. If that image is not available, we will request the right breast craniocaudal image.

At each study center, the study coordinators will request the screening mammogram from the appropriate clinics. It is likely that images from the screening mammogram might be obtained in one of three ways. First, if available, it would be preferable to have the image from a full-field digital mammography system. In this case, the image can be electronically transmitted to investigators at Northwestern University for the assessment of percent breast density. A second possibility is to have the mammogram digitized at the original institution and to transmit the digital image to Northwestern University. Using either of these two methods, it is possible to maintain confidentially by using only the parent study ID number and date of the mammogram to identify the image. The third possibility is that only a film screen mammogram is available from an institution that cannot digitize it. In this case, the study coordinator will place a label with the study ID over any identifying information (e.g., name) on the mammogram. The film mammogram will then be sent to Northwestern University where it will be scanned. The film will then be returned to the institution of origin.

At Northwestern University, a trained technician blinded to treatment status will determine the percent of breast fibroglandular density measured from the craniocaudal image. An automated analysis will be used to measure the quantity of fibroglandular tissue in the breast. This method consists of four steps for each digital image: 1) display of the digital mammogram, 2) determining the area of the entire breast (excluding lesion markers, nipple markers, and pectoralis major muscle along the chest wall), 3) determining a threshold signal value that determines the edges of fibroglandular regions, and 4) calculating the breast area (BA) and the fibroglandular tissue area (FA) within the breast area by summing the number of pixels within each region. Percent breast density, which is simply the ratio FA/BA x 100, will be calculated. Percent density provides a 2-dimensional quantitative estimate of breast density that has been shown to have high inter- and intra-reader reliability in selecting the threshold value and determining breast density in screen film mammography (SFM).

*10.5.5 Statistical Considerations*

**Study Design**

The goal of the parent study is to enroll 3000 total participants and to randomize 2400. Based on current recruitment rates, approximately 40% of the participants are women; therefore it is estimated that approximately 960 participants will be women. Of the women recruited we expect that 624 will be enrolled in the two-arm and 336 will be enrolled in the full-factorial study. Based on preliminary data that were collected from four study centers (NC, SC, GA and TX), it is estimated that at least 80% of women randomized will be eligible (i.e., no history of breast cancer) and will agree to participate in the mammography study.

|  | **Vitamin D** | **Calcium** | **Vit D and Calcium** | **Placebo** | **TOTAL** |
| --- | --- | --- | --- | --- | --- |
| Two-arm |  | 312 | 312 |  | 624 |
| Full-factorial | 84 | 84 | 84 | 84 | 336 |
| **Total randomized** | **84** | **396** | **396** | **84** | **960** |
| **No. of eligible women who agree to participate.** | **67** | **317** | **317** | **67** | **768** |

The design for this ancillary study is a 2 x 2 factorial design as follows:

|  | **Calcium - No** | **Calcium – Yes** | **TOTAL** |
| --- | --- | --- | --- |
| **Vitamin D – No** | 67 | 317 | 384 |
| **Vitamin D - Yes** | 67 | 317 | 384 |
| **TOTAL** | 134 | 634 | 768 |

**Statistical Analysis**

In preliminary analysis, we will ascertain the distribution of percent breast density to determine whether there are outliers or evidence of non-normality. If non-normality is indicated within any of the four study groups, then the density data will be log transformed before statistical analysis. Group differences in potential confounding variables are likely to be small due to the randomized nature of the study. However, we will examine descriptive statistics by treatment group for variables collected at baseline and at each follow-up visit, including demographics, hormone use, menopausal status, and BMI. We will compute means and standard deviations for continuous variables, medians and interquartile ranges for ordinal variables, and percentages by category for categorical variables. Potential group differences will be compared using analysis of variance (ANOVA) for continuous variables and chi-square tests for dichotomous or polychotomous variables. If group differences are observed, they will be included as covariates in analyses.

In this ancillary study, we are collecting the most recent mammogram prior to randomization as well as each mammogram post-randomization after being on study for at least one year and through the end of the study. The time from the pre-randomization mammogram to the first mammogram post-randomization is likely to vary among women. Therefore, to maximize a potential treatment effect, we will base our primary analysis on the mammogram where women have been on treatment for the longest period of time (i.e., a post-test comparison).

The primary method of statistical analysis for this ancillary study will be two-factor ANOVA or analysis of covariance (ANCOVA), to take advantage of the factorial nature of the design. This analysis will include a factor for Vitamin D (No, Yes), a factor for Calcium (No, Yes) and a Vitamin D x Calcium interaction term. The presence of a significant interaction will be assessed first and if the interaction term is statistically significant, and if this is due to the mean density for the combined Vitamin D + Calcium group being lower than the other three groups, then the secondary hypothesis will be supported and will show that both supplements synergistically reduce density more than each supplement alone. If the interaction term is not significant, then the marginal means in the factorial design will be tested using the main effect for vitamin D to test hypothesis 1, and the main effect for calcium to test hypothesis 2. (If the interaction term is significant, then main effects will still be tested, recognizing that there are differential effects). If group differences are observed, we will then conduct exploratory analyses to determine whether supplementation leads to a change in breast density (i.e., between pre-randomization mammogram and post-randomization mammogram). In addition, in exploratory analysis we will also use ANOVA to test the hypothesis that mammographic breast density is significantly lower in women randomized to the combination of vitamin D plus calcium compared to the placebo group.

We will also use ANOVA to test the exploratory hypothesis that the effect of supplementation with vitamin D on mammographic breast density is modified by polymorphisms at the 3' end of the vitamin D receptor gene. For this analysis, we will include a dichotomous variable to represent the presence or absence of the polymorphism, a dichotomous variable for vitamin D supplement (yes vs. no), and a cross-product term of these two variable. We will also include a dichotomous variable for calcium (yes vs. no) is in the above analyses calcium is found to be significantly associated with breast density.

Since all statistical tests are based on a priori hypotheses and are done within the context of the ANOVA or ANVOCA models, there will be no adjustment for multiple testing in either the analysis or sample size calculations.

**Power/Sample Size Considerations**.

Using a two-tailed test with an α=0.05, and 80% power, we computed the minimal detectable difference in mean percent breast density for each of the comparisons as described for each of the primary and secondary aims below.

Primary Aim 1: Our first primary aim is to determine if supplementation with vitamin D reduces mammographic breast density. For this comparison of vitamin D versus no vitamin D we expect 384 (317 + 67) women per comparison group. This sample size will have 80% power to detect an effect size of 0.20, where effect size is defined as the mean difference in mammographic density between the vitamin D and no vitamin D groups, divided by the within group standard deviation (assumed to be the same for both groups). The units for the mean and standard deviation are percent breast density denoted by ‘%’. In a large cross-sectional study of primarily Caucasian women aged 40-93 years, percent breast density was determined by analyzing mammograms (Vachon et al, 2002). In that study, a standard deviation of 15% was observed. Given the similar age range of women in that study and our proposed study, we based our power estimates assuming a standard deviation of 15%. Therefore, the minimal detectable difference between the vitamin D groups is 3% (=.20 x 15).

Primary Aim 2: Our second primary aim is to determine if supplementation with calcium carbonate (calcium) reduces mammographic breast density. For this comparison of calcium versus no calcium, we expect 634 (317 + 317) women in the calcium group and 134 women (67 + 67) in the no calcium group. This sample size will have 80% power to detect an effect size of 0.27. Assuming a standard deviation of 15% for percent breast density, the detectable difference between the calcium groups is 4.0% (=.27 x 15).

Secondary Aim 1: Our secondary aim is to determine if supplementation with both vitamin D and calcium reduces mammographic breast density more than supplementation with calcium alone or with vitamin D alone. This vitamin D by calcium interaction reflects the vitamin D effect in the absence of calcium (67 vs 67 women) compared with the vitamin D effect in the presence of calcium (317 vs 317 women). These sample sizes will have 80% power to detect an effect size of 0.54. Here, effect size is defined as (vitamin D effect in the absence of calcium – vitamin D effect in the presence of calcium)/within group standard deviation (grouping is the 4 treatment combination groups and the standard deviation is assumed to be the same for the 4 groups). Assuming a standard deviation of 15% for percent breast density, there is 80% power to detect a vitamin D vs no vitamin D mean difference in the presence of calcium that is 8% higher than the vitamin D vs no vitamin D mean difference in the absence of calcium. For example, if the mean percent density in the 67 women not on vitamin D and not on calcium is 30%, and the mean percent density in the 67 women on vitamin D and not on calcium is 27% (vitamin D effect in the absence of calcium is 30 – 27 = 3%), and if the mean percent density in the 317 women not on vitamin D but on calcium is 26%, then there is 80% power to detect a mean percent density in the 317 women on vitamin D and on calcium of 15% (vitamin D effect in the presence of calcium is 26 – 3 – 8 = 15%).

10.6 *Kidney Function with Vitamin D/Calcium*

This sub-study will investigate the effect of vitamin D and calcium supplementation on measures of renal function and blood pressure.

Subjects in the parent study who consent to this ancillary study will undergo the following additional procedures at their end of treatment (EOT) visit (either Year 3 or Year 5 EOT):

- Urine collection for measuring microalbuminuria
- Blood pressure measurement
- An additional vial of blood will be collected for measuring markers of kidney function (see below)

In addition, creatinine and calcium measurements previously performed on all randomized subjects in the parent study at baseline, year 1, and year 3 (for subjects with a 5 year follow-up colonoscopy) will be used in these analyses. Creatinine and calcium measurements will also be performed on blood samples collected on all randomized subjects in the parent study at the Year 3 and Year 5 End-of-Treatment visits and used in these analyses.

*10.6.1 Background*

Chronic kidney disease (CKD), assessed by either the presence of microalbuminuria or decreased kidney function, affects over 23 million people in the United States and is associated with a high risk of hypertension, cardiovascular events, mortality and progression to end-stage renal disease. Known risk factors for kidney disease include the presence of high blood pressure, diabetes mellitus, dyslipidemia, non-white race and older age. Novel risk factors for kidney disease progression are continuously being identified, including low 25-hydroxyvitamin D (25(OH)D) levels and elevated serum phosphate and FGF-23 levels.[^175-177^](#_ENREF_175)

Animal studies suggest plausible mechanisms whereby low 25(OH)D levels and high phosphate levels may be harmful. Activated vitamin D, 1,25-dihydroxyvitamin D (1,25(OH)2D), suppresses renin biosynthesis in mice and vitamin D deficiency stimulates renin production.[^178^](#_ENREF_178) In different animal models, 1,25(OH)2D or its analogs reduced proteinuria levels, preserved glomerular podocyte structure, [^179^](#_ENREF_179) decreased levels of transforming growth factor β1, an inducer of renal fibrosis, and inhibited mesangial cell proliferation, a marker of renal injury. [^180^](#_ENREF_180) High phosphate levels in rats lead to worse kidney function.[^181^](#_ENREF_181) In the setting of kidney disease, vitamin D supplementation may lead to higher phosphate levels through increased gut absorption but calcium therapy can act as a phosphate binder in the gut and may prevent a rise in phosphate levels in those treated with vitamin D.

High blood pressure can be both a consequence of kidney disease and can independently cause kidney disease. Low vitamin D levels have been associated with the development of incident high blood pressure. [^182^](#_ENREF_182) Some trials of vitamin D therapy have noted lower blood pressures in the vitamin D treated group. [^183-185^](#_ENREF_183) However, these trials had small sample sizes and were not powered to evaluate changes in blood pressure.

Thus far, vitamin D and phosphate binding therapies have not yet been tested in a large randomized clinical trial powered to evaluate kidney disease outcomes. The Vitamin D/ Calcium Polyp Prevention Study is the ideal setting in which to test the effects of vitamin D and/or calcium on kidney function in a double blind, placebo controlled setting.

*10.6.2 Objectives*

The primary aim is to investigate the effect of calcium and/or vitamin D on the prevalence of microalbuminuria, a marker of kidney damage, at the end of 3 or 5 years of treatment with calcium and vitamin D, calcium alone or vitamin D alone versus placebo.

*Hypothesis: Vitamin D treatment will be associated with a lower prevalence of microalbuminuria.*

The secondary aims are as follows:

1. To evaluate the effects of calcium and/or vitamin D on blood pressure at the end of 3 or 5 years of treatment with calcium and vitamin D, calcium carbonate alone or vitamin D alone versus placebo.

*Hypothesis: Vitamin D therapy will be associated with significantly lower blood pressure.*

1. To evaluate the effects of calcium and/or vitamin D on kidney function as measured by serum creatinine measurements at baseline, year 1, year 3 and year 5 in all participants of the parent study with available creatinine measurements. Additional urine and serum markers of kidney function to be measured at the end of 3 to 5 years of treatment will include:
   1. Urine: markers of tubular damage including KIM-1 and NGAL
   2. Serum: creatinine (to be assayed at the lab used for baseline measurement),calcium and cystatin C, an additional marker of kidney function
   3. Serum: bone markers and other markers of kidney disease progression including serum phosphate, alkaline phosphatase, intact parathyroid hormone (PTH) and fibroblast growth factor-23 (FGF-23) levels.

*Hypothesis: Treatment with vitamin D and calcium will result in better kidney function compared to placebo*.

*10.6.3 Recruitment and Enrollment Procedures*

The parent study defines the pool of potential subjects and those subjects who are willing to participate will be consented at the in-person End-of-Treatment visit (which is part of the parent study procedures) prior to their 3-year or 5-year follow-up colonoscopy. Participants will be reimbursed $15 for their time and effort after completion of the specimen collection.

*10.6.4 Specimen/data Collection and Analysis*

10.6.4.a Urine collection

Subjects will be instructed on clean catch urine collection techniques and will be provided with a pre-labeled sterile container for urine collection. After they have completed the urine collection they will give the urine to the research coordinator for processing.

10.6.4.bBlood pressure measurement

An automatic Sphygmomanometer will be used to provide a uniform measurement of blood pressures. All staff will be trained in the proper procedure for blood pressure measurement.

10.6.4.c Blood collection

Additional blood samples will be collected for this sub-study while the participant is undergoing the Year 3 or Year 5 End-of-Treatment blood collection.

10.6.4.d Processing and Shipping

Specimens (aliquots of blood and urine) will be shipped from the Study Centers to the Clinical & Molecular Pharmacology Shared Resource Laboratory at the Dartmouth Coordinating Center. These specimens will then be batched and shipped to the Albert Einstein College of Medicine for analysis.

Urines

Urine samples will be promptly frozen at -20°C or below pending transfer to a -70°C freezer for storage until shipment to the Coordinating Center.

Bloods

Blood samples will be sent to local labs for analysis (creatinine) or promptly frozen at -20°C or below pending transfer to a -70°C freezer for storage until shipment to the Coordinating Center.

**10.6.4.e Analyses**

*Measurement of Serum Creatinine, Calcium, Cystatin C, Phosphate. Alkaline phosphatase, FGF-23 and intact PTH*

At the Albert Einstein College of Medicine Institute for Clinical and Translational Research laboratory, specimens will be received and appropriately stored until analysis. Analyses will be batched and analyses will be performed once all of the specimens are received.

*Measurement of urinary albumin, creatinine, KIM-1 and NGAL*

Analyses will be batched and performed by the Albert Einstein College of Medicine Institute for Clinical and Translational Research laboratory using standard methods.

*10.6.5 Sample Size and Power*

Of the 2259 participants in the parent study, we plan to recruit approximately 900 into this sub-study. In the Framingham Offspring cohort with a mean age of 59 years, 12.2% had microalbuminuria, defined as at least 30 mg/g creatinine. [^186^](#_ENREF_186) In the NHANES III survey, which includes younger participants, 8.5% of participants had a urinary albumin:creatinine ratio greater than 30 mg/g.[^187^](#_ENREF_187) Assuming the background prevalence of microalbuminuria in the trial is 12% and an alpha of 0.05, we will have 80% power to detect a relative risk of 0.64 for microalbuminuria comparing the vitamin D groups to the placebo group with 900 participants. We will therefore aim to recruit 900 participants for the urinary albumin measurement. In the Framingham Offspring Study, the standard deviation for systolic blood pressure is approximately 20 mm Hg. [^186^](#_ENREF_186) Assuming an alpha of 0.05 and 900 participants, we will have greater than 85% power to detect a difference of 3 mm Hg between the vitamin D and placebo groups. For the repeated measures analysis for creatinine we will only use data from participants who have had all of their creatinines measured at the same laboratory (n≈1500). Having a sample size of 1500 for repeated measures analysis to evaluate changes in creatinine with an alpha of 0.05 gives us 90% power to detect a difference of 0.1 mg/dL in creatinine measurements. Evaluating changes in bone serum biomarkers, assuming an alpha of 0.05, we have >90% power to detect a difference of 1 standard deviation between the groups.

*10.6.6 Statistical Analyses*

Specific Aim #1: All analyses will be performed and outliers will be verified by re-assay, if urine is available. Because this is a post-hoc analysis of a randomized clinical trial and data will not be available on the entire clinical trial population, we will create a table to evaluate whether baseline characteristics are equally distributed among the participants in the sub-study. The proportion of participants in each randomized group with microalbuminuria (defined as >30 mg/g creatinine) will be compared using a chi-square test. If there are imbalances in the baseline characteristics, we will perform a logistic regression with the outcome albuminuria adjusting for characteristics that were imbalanced at baseline. In addition, we will compare medication use at follow-up to evaluate whether there were imbalances in NSAID and ACE inhibitor and Angiotensin Receptor Blocker (ARBs) use between the randomized groups.

Specific aim #2: The mean blood pressure of the 3 measurements will be used for all analyses. Baseline characteristics of the randomized groups included in this sub-study will be compared. In addition, the use of anti-hypertensive medications at the time of the blood pressure measurement will be compared between the groups. We will first compare the systolic and diastolic blood pressures among the 4 randomized groups using ANOVA. We will also compare vitamin D to no vitamin D using Student’s t-test. If characteristics are not balanced between the groups, we will use multivariable adjusted linear regression to evaluate whether treatment had an effect on systolic and diastolic blood pressures separately.

Specific aim #3: All data will be checked for outliers and specimens will be re-assayed for extreme outliers. Creatinine measurements will be converted to estimated glomerular filtration rates using the CKD-Epi equation. [^188^](#_ENREF_188) We will compare the prevalence of CKD (defined as an estimated GFR <60 ml/min/1.73m^2^) between the 4 treatment arms using chi-square test. We will also used random effects models with repeated creatinine measures which can account for missing values to compare serial creatinines between the 4 treatment groups. To evaluate whether calcium and/or vitamin D supplementation over 5 years affected serum phosphate, alkaline phosphatase, intact PTH and FGF-23 levels, we will compare levels between the intervention groups and the placebo group using appropriate methods for skewed data.

*10.6.7 Human Subjects Research*

*10.6.7.a Human Subjects Involvement and Characteristics*

The participants in this ancillary study are already enrolled in a 3 to 5-year randomized clinical trial of calcium and/or vitamin D. They have already consented to participate in the parent study. For this ancillary study, they will undergo an additional informed consent procedure during their end-of-treatment visit for the parent study and, if consented, will have their blood pressure measured, give a urine sample and have additional blood samples taken at the end-of-treatment blood draw for the parent study.

We expect that the population for this sub-study will be similar to that of the overall parent study. The mean age at enrollment was 58.1 (standard deviation 6.8), the participants were 37% female and 83% white, 8.1% black, 8.5% other race and 6.5% of Hispanic ethnicity.

The study will take place at the sites of the parent trial: Dartmouth Hitchcock Medical Center, Cleveland Clinic Foundation, University of Colorado, Emory University, University of Iowa, University of Minnesota, University of North Carolina, University of South Carolina, University of Southern California, University of Texas and University of Puerto Rico and their related practice sites.

*10.6.7.b Sources of Materials*

The participants in this sub-study will give urine and blood samples and will have their blood pressures measured. The parent study is collecting information on demographics, co-morbid health conditions, medications and other health information. The new data will be linked to existing data via unique identifiers.

*10.6.7.c. Potential Risks*

The additional physical risks associated with the sub-study are those associated with the collection of the urine, blood and blood pressure, which are outside of the standard of care. Drawing blood, or venipuncture, may result in bruising to the arm or on rare occasions, vasovagal syncope. Blood pressure measurements involve some discomfort as the sphygmomanometer is inflated. There are no significant risks to a spot urine collection. All these risks are detailed in the informed consent document. There may be additional risks of breach of confidentiality with the additional measurements taken. All study personnel are already trained in the importance of maintaining confidentiality and all data for analysis at the Albert Einstein College of Medicine will be de-identified.

*10.6.7.d. Adequacy of the Protection against Risks*

*10.6.7.d.1 Recruitment and Informed Consent*

All patients included in this sub-study have already undergone 3 or 5 years of a randomized clinical trial with blood testing and colonoscopies. They will undergo a separate informed consent process for this sub-study detailing the rationale for the study and risks and benefits associated with participation.

*10.6.7.d.2. Protection against Risk*

All staff are trained to maintain complete confidentiality. All data transferred to the Albert Einstein College of Medicine will be completed de-identified. Venipuncture and blood pressure measurements will be conducted by experienced phlebotomist and technician with the patient seated. The Data and Safety Monitoring Committee for the parent trial will serve in this capacity for the ancillary study, by monitoring for potential complications and recommending whether the trial should continue.

10.6.7.e. Potential Benefits of the Proposed Research to the Subject and Others

The participants of this trial will likely not directly benefit from the kidney functions ancillary study. However, the results of this trial may impact the treatment for patients with kidney disease and high blood pressure with an inexpensive therapy, calcium and vitamin D.

*10.6.7.f. Importance of Knowledge to be Gained (Risk: Benefit Ratio)*

High blood pressure, kidney disease and microalbuminuria are all associated with high risk of cardiovascular disease and mortality. A new, inexpensive therapy to treat high blood pressure, prevent the progression of kidney disease and/or albuminuria would have far-reaching public health implications. The risks to the participant for this ancillary study are fairly minimal and involve the collection of blood, urine and blood pressure measurements at a single time point at the end of a randomized clinical trial evaluating colonic polyp recurrence. The risks detailed above are fairly minimal in comparison to the knowledge to be gained.

*10.6.7.g. Data Safety Monitoring Plan*

The Data and Safety Monitoring Committee for the parent trial will serve in this capacity for the ancillary study, by monitoring for potential complications and recommending whether the trial should continue.

*10.6.7.h. Inclusion of Women and Minorities*

We expect that the participants in the kidney function sub-study will have the same gender and race/ethnicity make-up as the parent trial. Therefore we expect 37% female participants and 83% white, 8.1% black, 8.5% other race and 6.5% of Hispanic ethnicity. We will not exclude any participants based on gender or race/ethnicity.

10.6.7.i. Inclusion of Children

Children are not enrolled in the parent study, the Vitamin D Polyp Prevention Trial because children generally do not get colonic polyps and if they do occur in children, they are part of a genetic syndrome and probably would not be affected by vitamin D and calcium supplementation. Therefore, no children will be recruited into the kidney functions sub-study.

*10.7* Participant Beliefs and Bias Study

This ancillary analysis is under the direction of Dr. Judith R. Rees at the Geisel School of Medicine at Dartmouth.

*10.7.1 Background and rationale*

The randomized, placebo-controlled trial uses allocation concealment and blinding of participants and investigators to minimize bias in patient selection, adherence, and ascertainment of outcomes. Beliefs about the assigned treatment (allocation belief) and its efficacy (efficacy belief), and treatment preference may affect adherence, health behaviors, attrition, and health endpoints. We will study the relations between these beliefs, blinding, adherence and health outcomes. Our findings will help develop strategies to prevent and/or correct for belief-related biases in future trials.

# *10.7.2 Objectives*

# Using data already collected during the trial (Section 5.15), we will:

# 1. Identify symptoms and other predictors of subsequent allocation belief at randomization, year 2, and end of treatment (EOT).

# 2. Assess how subjective and objective health outcomes are affected by allocation beliefs, efficacy beliefs, and preference.

# 3. Assess the effects of allocation beliefs, efficacy beliefs, and preference on adherence.

*10.7.3 Data collection*

Data collection (approved previously) is conducted as part of the routine interviews for the parent study, as well as separate mail-back study methods surveys.

*10.7.4 Analyses*

Allocation beliefs will be classified as “Believe Active” (A) for any active treatment option in the 4-arm study or calcium+vitamin D in the 2-arm study;. “Believe Placebo” (P) for placebo in the 4-arm study; calcium in the 2-arm study; or “Don’t Know (DK). We will analyze all subjects, and separately those in the 4-arm and 2-arm studies. We will assess the distribution of forced guesses of those who said DK and incorporate the appropriate analyses and sensitivity analyses to show the effect of pooling forced guesses with Active or Placebo guesses. A 2-sided p-value <0.05 will be considered statistically significant. We will not adjust for multiple testing. Our analyses will be based primarily on describing the data and regression modeling..

Aim 1. Identify predictors of allocation belief at randomization, year 2, and EOT.

At different times during the trial, we will classify the reasons subjects provided for their allocation beliefs and explore the factors that predict beliefs. We will explore the factors associated with misunderstanding of equipoise and randomization.

Hypothesis 1. Allocation beliefs change during the course of a double-blinded trial. We will describe the distributions of beliefs, changes in beliefs from randomization to year 2 [EOT], and use regression models to identify factors related to changes of belief.

Aim 2. Examine how subjective and objective health outcomes are affected by allocation beliefs, efficacy beliefs, and preference. We will assess how beliefs affect subsequent symptoms and on colorectal adenoma, using multivariable regression.

Hypothesis 2a. Allocation beliefs at randomization [year 2, EOT] predict the occurrence of each of five specified health effects, after adjustment for a history of that same health effect. We will use multivariable regression models of the relations between beliefs and subsequent symptoms identified during routine bi-annual telephone surveys.

Hypothesis 2b. Allocation beliefs at randomization [year 2, EOT] predict a significant change in each of five SF-36 scores during the study, independently, or by interaction with efficacy beliefs about that health effect, after adjusting for baseline score and randomized treatment. Hypothesis 3 is analogous to Hypothesis 2, but uses SF-36 data on general health, physical functioning, bodily pain, vitality, and mental health.

Aim 3. Examine how adherence is affected by allocation/efficacy beliefs, and preference.

We will model subjects’ adherence, in relation to their preferences, efficacy beliefs, and allocation beliefs at different times, and demographic and medical factors.

Hypothesis 3. The proportion of pills taken during run-in is associated with allocation beliefs at randomization, after adjustment for baseline efficacy beliefs and preferences. We will develop a model for adherence including allocation belief; efficacy belief, preference, and repeat these analyses for adherence averaged over the first two years. We will develop models to predict completion of the trial on study pills.

10.7.4 Sample size

We will use all 2,813 enrollees where possible, and for other analyses, the 2,259 randomized subjects. For continuous outcome measures, we will have 90% power to detect a difference of 0.09 SD at baseline, and 0.11 SD at year 2, based on t-tests comparing allocation beliefs. For multivariable analyses, assuming that each additional predictor variables requires a 10% increase in sample size, we will be able to detect adjusted differences of 0.15 SD at baseline and 0.20 SD at Year 2 or EOT in models containing 20 additional predictors. For binary outcomes, at baseline, we will have 90% power to detect an odds ratio <1.3 at baseline, year 2 and EOT. For multivariable analyses assuming the outcome has prevalence of just 10% of the total sample, model estimates will be reliable with 28 predictors for baseline data models and 16 predictors for year 2 models. Hence our study sample is sufficient for the analyses described.

10.7.5 Additional Risks

This sub-study presents no additional physical risk to the subjects. Confidentiality of data is well protected, as described earlier.

10.7.6 Benefits and importance of knowledge to be gained

There is no direct benefit to subjects. Our results may improve randomized controlled trial methodology; in that case, society will benefit from improved trials in the future.

10.7.7 Inclusion of women, minorities

The sub-study uses data from all subjects in the parent study.

10.8 Colds and Flu study

This ancillary study is under the direction of Dr. John A. Baron, PI for the parent study at the Geisel School of Medicine at Dartmouth.

10.8.1 Background and rationale

Observational studies indicate that vitamin D may play a role in the prevention of infectious diseases. We will evaluate the effects of vitamin D supplementation on the incidence and duration of upper respiratory tract infection (URTI), influenza-like illness (ILI), and acute gastrointestinal illness (AGI), by adding surveillance for these three illnesses to our on-going randomized controlled clinical trial of vitamin D and/or calcium supplementation for the prevention of colorectal adenomas.

10.8.2 Objectives

Our specific aims are:

To collect health dairies recording the incidence and duration of common infectious illnesses in approximately 800 subjects enrolled in our clinical trial of vitamin D and calcium.

To assess the effects of vitamin D supplementation on the risk of upper respiratory tract infections and explore effects on gastrointestinal illnesses, using formal standardized case definitions of URTI, ILI, and AGI

Our primary hypotheses are:

Supplementation with vitamin D_3_ (1000 IU/day) reduces the number of winter episodes of URTI.

Supplementation with vitamin D_3_ (1000 IU/day) reduces the number of winter days affected by URTI.

Our exploratory hypotheses are:

Supplementation with vitamin D_3_ (1000 IU/day) reduces the number of episodes of URTI over a year of observation.

Supplementation with vitamin D_3_ (1000 IU/day) reduces both the number of winter episodes of ILI and the number of winter days affected by that illness.

Supplementation with vitamin D_3_ (1000 IU/day) reduces both the number of episodes of AGI and the days affected by that illness.

In addition, we will (i) compare the recent reporting of colds and ILI by subjects telephoned every 6 months among those randomized to vitamin D_3_ (1000 IU/day) supplementation or placebo; and (ii) examine the impact of serum levels of 25-hydroxyvitamin D_3_ (25-OHD) on subject’s experience of symptoms during the study.

10.8.3 Data collection

Questionnaires (implied consent) were administered as described in Section 6.2.

25-OHD was measured as described in Sections 5.12 and 9.1 - 9.3.

10.8.4 Analyses

URTI: We define a common cold or upper respiratory tract infection (URTI) as the presence of 2 or more of the following: runny nose, nasal congestion, sneezing, sore throat, cough, swollen or tender neck glands.

ILI: We define Influenza-like illness (ILI) as fever, plus 2 or more of the following: sore throat, cough, muscle ache, headache.

AGI: We will base our definition of acute gastrointestinal illness on Payment’s “highly credible gastrointestinal illness”: either 1) vomiting or watery diarrhea; or 2) nausea or soft diarrhea with abdominal cramps.

Further, our definition of an episode of illness requires 6 symptom-free days before a subsequent episode will be said to have occurred. We will require this symptom-free interval because we assume that symptoms occurring toward the end of an illness may not necessarily fulfill the case definition but will still be part of the episode. For example, on days 1, 2 and 3, a participant’s symptoms fulfill the definition of a common cold (sore throat and nasal congestion). On days 4 and 5 only a mild sore throat persisted, but the nasal congestion recurred on days 6 and 7, which together with the sore throat fulfill the case definition for those two days. According to our definitions, this constitutes only one episode of URTI. For the purposes of estimating “days of illness”, we will use our case definitions strictly, so that day 4 and 5 are excluded, and there were only 5 days of URTI altogether.

Statistical methods

Primary end points will be the number of episodes of each type of illness and the total number of days of affected by the illness. Because of the seasonally low vitamin D levels in winter, our main hypothesis is that supplementation will have an effect during those months, rather than during the summer.

For the analysis of the number of illness episodes, we will use Poisson regression to estimate the effect of treatment assignment to vitamin D. The primary analyses will be by intention-to-treat – i.e. we will ignore subject compliance in the analysis, which will be based solely on the randomized assignment. We adjust for calcium treatment assignment in all analyses. We will also pay special attention to over-dispersion or under-dispersion, as assessed by the usual chi-squared testing. If this is statistically significant, we will use robust methods to estimate standard errors and recalculate the confidence interval for the treatment effect. Other analyses e.g. of the days affected by illness will proceed in the same spirit.

We will compare randomized treatment groups in relation to the probability of any self reported health outcome ascertained during 6 monthly interviews (colds, ILI).

10.8.5 Sample size

Based on national data and data from our own study, we estimate that placebo subjects experience between 0.6 and 0.9 URTI winter UTRI per year on average. With our expected recruitment, we will have excellent statistical power to detect vitamin D relative risks of 0.7 or lower, and adequate statistical power for relative risks of 0.8. Based on national data, we estimate that placebo subjects experience between 1 and 3 days affected by a winter URTI per year on average. With our expected recruitment, we will have excellent statistical power to detect vitamin D relative risks of 0.8 or lower.

10.8.6 Additional Risks

This sub-study presents no additional physical risk to the subjects. Serum 25-OHD was already collected for the parent study. Confidentiality of data is the primary concern, but the data are not sensitive and are well protected as described earlier.

10.8.7 Benefits and importance of knowledge to be gained

There is no direct benefit to subjects. We aim to describe whether vitamin D prevents colds and flu; the answer to this question will benefit society as a whole.

10.8.8 Inclusion of women, minorities

The sub-study was available to subjects of all demographic categories within the parent study.

***11.0 Safety Monitoring and Reporting Adverse Events***

*11.1 Definition, Monitoring and Follow-up*

An adverse event is any condition that appears or worsens after the subject is enrolled in an investigational study. Adverse events may be discovered as a result of study lab measurements or during regular study interviews (every six months). As a safety measure, blood levels of calcium, creatinine and 25-OH vitamin D will be monitored one year, and where applicable three years after randomization. At each regularly scheduled interval contact (every six months) study coordinators will monitor symptoms, medical diagnoses and hospitalization as part of the subject case report form. Adverse events may also be discovered by subject-initiated contact at any time. All adverse events that meet study specific criteria are documented on an Adverse Event Report Form. There are three types of events that have been defined: Perceived Toxicity, Diagnosis and Out-Of-Range Lab Result. All adverse events, including laboratory abnormalities that in the opinion of the Clinical Director are clinically significant, will be followed up according to good medical practice, and documented as such*.*

*11.2 Adverse Event Information and Severity*

The following information will be collected on the Adverse Event Report Form: start and stop dates, severity (grade), relationship to study agents, and whether or not the subject stopped taking the study agents due to the adverse event. Severity will be graded by a numerical score according to the NCI Common Terminology Criteria for Adverse Events (CTCAE - version 3.0 or newer). Events not included in the defined NCI CTCAE will be scored according to their impact on the subject’s abilities to perform daily activities as follows:

a. Mild (causing no limitation of usual activities)

b. Moderate (causing some limitation of usual activities)

c. Severe (causing inability to carry out usual activities)

*11.3 Serious Adverse Events*

Serious adverse events are defined as those events that meet any of the following criteria:

- Results in death
- Is immediately life threatening
- Requires inpatient hospitalization or prolongation of existing hospitalization
- Results in persistent or significant disability or incapacity
- Is a congenital anomaly or birth defect

*11.4 Reporting*

Adverse events will be reported to study center IRBs according to local IRB policy. Study Centers are responsible for reporting all adverse events to the Project Coordination Center according to the requirements and timelines set forth in the standard operating procedures for this study. Unexpected serious adverse events possibly related or related to the study agents will be reported on an expedited basis to the NIH, the FDA (as required by law), Pfizer Pharmaceuticals, previously Wyeth Consumer Healthcare, and the Safety and Data Monitoring Committee. In addition, every six months the Safety and Data Monitoring Committee will monitor study-wide adverse events and the overall conduct of the trial. Subsequently, a report summarizing study-wide adverse events will be sent to each Study Center for submission to the individual IRBs.

***12.0 Data Management***

*12.1 Central database*

The central database was developed using a SQL-based database management application and will be administered by the Informatics group within the Section of Biostatistics and Epidemiology at Geisel School of Medicine at Dartmouth. Any data errors or inconsistencies detected after data entry will be automatically tracked, communicated and resolved using the Informatics center's web-based application. An audit trail of all data changes over the life of the study will be maintained. The database is located on a server protected by firewalls using stateful inspection firewalling and protocol and application inspection. Access to the database server will no be allowed by users on computers outside of the firewall protected zone.

*12.2 Website*

This study will employ real-time web-based data entry at the Study Centers. A central study website will provide ready and secure access to entry interfaces and current study information including contact information for all study personnel, the study protocol, policies and procedures, updated forms and questionnaire versions, and real-time reports. The web server will run Apache on Linux OS and Microsoft IIS Server on Windows NT, providing a high level of security through authentication and 128 bit encryption using SSL. The website was developed using HTML, Java, Javascript, and PDF-formatted documents, and will be accessible from all current browsers. A separate study web page will be used to provide access to subjects for self-completion of interval questionnaires.

*12.3 Data Entry*

Most data entry will occur when the study coordinator is able to interview subjects in a location with a networked computer so that data can be entered in real time into forms or questionnaires served from the Dartmouth web server. The study coordinator will access the database interface through the study URL, be authenticated, link to the appropriate form, and enter data directly into the central database. Data will be checked in real time via scripted validation for range, data type, field length, and logical consistency with related data fields. These routines will prompt the coordinator to reconcile inappropriate or inconsistent data, and will not permit missing mandatory data. Critical data (such as study identification number) will be double-keyed. Additionally, to reduce entry error, appropriate data will be pulled from related tables within the central database and will be used to create pull-down menus and pick-lists for data entry whenever possible. Study coordinators will be encouraged to use this electronic (validated) data entry whenever possible. Subjects will also be able to enter data directly into interval questionnaires on the web.

If internet access is temporarily disabled or unavailable, a low-tech backup (paper forms) will be provided to ensure timely data collection under any circumstance. All data that is collected on paper forms will subsequently be entered into the electronic study forms on the web by the study coordinator. Electronic data entry must occur within 7 days of data collection. Double entry will be required.

Electronic study questionnaires, which are administered to subjects by their study coordinator, will include the intake questionnaire (to be used at the intake appointment), the first phone interview questionnaire (to be used during the initial phone interview at randomization assessment), the interval questionnaire (to be used during interviews mainly by phone), every six months throughout the study, the end of treatment questionnaire, and the observational follow up questionnaire. Paper versions of these questionnaires are provided for completion by subjects as necessary (post randomization only), and for IRB review.

*12.4 Computer Security and Safety*

All computer systems and programs will be password protected, and all electronic communications of study and other confidential information will be encrypted. Good computer security practice (restricting physical access to machines, prohibition of password sharing, logging off computers after work hours or when away from the machine) will be required of all study personnel. Virus protection software will be installed on each study machine. The virus detection tools will be used, maintained, audited and, if necessary, updated on all computers and pathways into the system. All computer systems will be updated regularly with any applicable operating system and application security patches. System access to computer systems will be audited. Redundant backups and off-site backup storage will allow for quick restoration of data in the unlikely event that a hardware failure, disaster, or security breach should occur. Servers and backups will be located in a secured location with access limited to authorized personnel.

*12.5 Archiving*

All study raw data, forms, documents, software programs, software applications and computer data files will be indexed and archived routinely. Strict version control of documents and software applications will be instituted. Retention of study documentation after study completion will conform to FDA and NIH requirements.

*12.6 Reports*

Standardized study management reports will be generated monthly during the recruitment phase of the study and thereafter at least every quarter for the Project Coordinator at the Dartmouth Project Coordination Center. These reports will be used to track study progress including subject enrollment, randomization, compliance, subject status changes, and study events. The data will be reported for each Study Center individually and summarized for the study as a whole.

Every six months, a standardized report will also be generated for the Safety and Data Monitoring Committee meeting. This report will include additional information on clinical events and adverse events that is coded by blinded treatment group. Other than the study statistician and statistical analyst, no study personnel will see this report.

***13.0 Statistical Methods***

### 13.1 Hypotheses

Our main hypotheses are:

1. Supplementation with vitamin D_3_ (1000 IU/day) reduces the risk of new adenomas in patients with a recent history of these tumors.

2. Supplementation with calcium carbonate (1200 mg elemental calcium/day) reduces the risk of new adenomas

3. Supplementation with both vitamin D_3_ (1000 IU/day) and calcium carbonate (1200 mg elemental calcium/day) reduces the risk of new adenomas more than supplementation with calcium carbonate alone.

Our secondary hypotheses are:

4. Supplementation with vitamin D_3_, or with vitamin D_3_ or with calcium carbonate, reduces the risk of new advanced colorectal lesions (colorectal cancer and adenomas with an estimated diameter of one centimeter or greater, those with tubulovillous or villous histology, or those with advanced dysplasia).

5. Supplementation with vitamin D_3_ will have a greater effect among individuals whose initial serum 25-OH vitamin D level is less than the overall study median, compared with subjects whose levels are greater than the median.

6. The effect of supplementation with vitamin D_3_ will be modified by polymorphisms at the 3' end of the vitamin D receptor gene.

### 13.2 Sample Size Justification

The sample size for this trial will be approximately 3000 enrolled subjects. Based on our previous trials, we expect that at least 80% of subjects will complete the run-in period, leaving 2400 patients randomized. Of these, we expect that 60% will be male and 40% female. Assuming that 35% of females agree to be randomized to calcium, we expect to have 1776 subjects randomized in the full factorial design and an additional 624 women (taking calcium) randomized to vitamin D or placebo. In our previous trials, we obtained follow-up examinations from at least 95% of subjects; however, we will assume conservatively that the follow-up rate will be 92%. This translates to endpoint data for 408 subjects randomized to each of the four supplement combinations in the factorial design (1632 in total) and for an additional 287 females randomized to each of the two vitamin D groups (574 in total). For the primary comparison of vitamin D versus no vitamin D, we expect 1103 subjects (408 + 408 + 287) per group with follow-up data. For the primary comparison of calcium versus no calcium, we expect 816 subjects (408 + 408) per group with follow-up data. For the primary comparison of vitamin D + calcium versus calcium alone, we expect 695 subjects (408 +287) per group with follow-up data.

Data from our latest trial -- the Aspirin/Folate Polyp Prevention Study -- indicate a three year adenoma occurrence rate of 45% among placebo subjects (who were asked to avoid NSAID use). NSAID use by the study subjects may lower the expected adenoma occurrence rate in the proposed study. Assuming that aspirin confers a 20% decrease in occurrence risk and 33% of subjects take aspirin daily, we would expect the overall occurrence rate in placebo subjects to be 42%. However, we expect that the subjects who enroll in this study will have lower risk profile overall because fewer high-risk subjects will be recruited. Thus, for the purpose of power computations, we assume that the occurrence rate among placebo subjects will be 37%. Power calculations are based on standard formulae for power of the χ^2^ test and assume no interaction between the supplements. Power for the vitamin D effect assumes a calcium relative risk of r_c_ = 0.80, and power for the calcium effect assumes a vitamin D relative risk of r_d_ = 0.75. Table 1 (below) shows the statistical power to detect various differences in occurrence risk for the primary comparisons of adenoma occurrence rates. The results indicate that we will have good statistical power to detect relative risks of 0.80 or less for the marginal vitamin D effect and the marginal calcium effect. We will have adequate power to detect a relative risk of 0.75 or less for the effect of vitamin D in the presence of calcium.

The detectable relative risk for the vitamin-D-by-calcium interaction effect is approximately r_cd_ = 0.60 (or 1.67). This was determined based on a z-test comparing log relative risks assuming adenoma occurrence rates as follows: placebo alone, p; calcium alone, r_c_p, vitamin D alone r_d_p, and vitamin D + calcium, r_cd_r_d_r_c_p, where r_c_ = 0.80, r_d_ = 0.75 and p = 0.37.

| Table 1. Statistical power* for primary study comparisons | | | | | | |
| --- | --- | --- | --- | --- | --- | --- |
|  | |  | Relative Risk | | | |
| Comparison (expected control occurrence) | N per group | | 0.70 | 0.75 | 0.80 | 0.85 |
| Vitamin D vs. no vitamin D (32%) | 1103 | | > 0.99 | 0.99 | 0.92 | 0.70 |
| Calcium vs. no calcium (32%) | 816 | | > 0.99 | 0.95 | 0.82 | 0.57 |
| Vitamin D + calcium vs. calcium (30%) | 695 | | 0.97 | 0.88 | 0.70 | 0.46 |
| *Statistical power is based on a chi-squared test with a 2-sided alpha level of 0.05. Calculations assume a baseline occurrence rate of 37% among placebo subjects and no interaction between the supplements. Power for vitamin D comparisons assumes a relative risk of 0.8 for calcium, and power for calcium comparisons assumes a relative risk of 0.75 for vitamin D. | | | | | | |

We will also have good statistical power to detect interaction effects between vitamin D supplementation and VDR genotype. The total expected sample size of 2206 patients with follow-up data will be used in these analyses, since all patients will be randomized in the vitamin D component of the study, and all patients will be assessed for VDR genotype. The statistical power calculations, given in the table below (Table 2) as the interaction relative risks detectable with 80% power, assume that VDR genotype is classified as two groups (positive and negative), the prevalence of the variants ranges from 20% to 50%, and the marginal relative risk for vitamin D supplementation is 0.75. The calculations also assume that the adenoma occurrence rate in the control group is 32% and VDR-genotype has no marginal effect on recurrence rates. For interaction effects between vitamin D levels (above versus below the median) and vitamin D supplementation, the same computations apply, using the 50% prevalence.

| **Table 2. Detectable relative risks for the interaction effect of vitamin D supplementation and VDR genotype.** | | | | |
| --- | --- | --- | --- | --- |
|  | Prevalence of VDR genotype | | | |
|  | 20% | 30% | 40% | 50% |
| Detectable RR | 0.59 | 0.64 | 0.66 | 0.67 |

### 13.3 Methods for Randomization and Stratification

Treatment assignments will be randomly generated using a computer program. Separate assignment lists will be generated for each stratum. Stratification factors are gender, Study Center, anticipated follow-up interval (3 or 5 years) and whether the patient is participating in both the vitamin D and calcium components or only the vitamin D component (the latter component is only for women who wish to take calcium supplements). Treatment assignments will be made in blocks to ensure equal numbers of patients across the treatment groups at various time intervals. The size of the blocks will vary in a random fashion. Randomization will occur only once a patient satisfies all eligibility criteria, including a successful run-in period.

### 13.4 Outcome Measures

For each subject, we will tabulate the number of mucosal lesions found, and record their size, histological characteristics, and location in the bowel. Each polyp will be classified (using standard criteria) as neoplastic (tubular, tubulovillous, villous, or serrated adenomas or cancer), hyperplastic, hamartomatous, retention, lymphoid, or other. In addition, for the neoplastic polyps, a statement with regard to the degree of atypia present will be made using standard criteria.

The main end point of the trial is the occurrence of neoplastic polyps of the large bowel in the interval between randomization and the anticipated follow-up colonoscopy (3 or 5 years after the qualifying exam). As in our previous trials, our primary endpoint will be a dichotomous measure of any occurrence of neoplastic polyps (adenomas). Secondary analyses will focus on the number and size of neoplastic polyps, the occurrence of advanced adenomas, and effects within subgroups defined by baseline 25-OH vitamin D levels and by VDR genotype.

### 13.5 Statistical Analysis

All analyses will be conducted according to the principle of intention to treat. The analysis population will consist of all randomized patients, with patients being grouped according to randomized treatment assignment regardless of whether this treatment was actually delivered.

To address our primary hypotheses, the primary statistical analysis will use contingency tables and standard χ^2^ tests to compare adenoma occurrence between treatment groups. There will be three primary comparisons: 1) vitamin D versus no vitamin D, and 2) calcium versus no calcium and 3) vitamin D + calcium versus calcium alone. Comparison 1 will collapse numbers of subjects across calcium supplementation groups to assess the marginal effect of vitamin D. Comparison 2 will collapse numbers of subjects across the vitamin D supplementation groups to assess the marginal calcium effect and will exclude women who do not consent to randomization in the calcium component of the study. Comparison 3 will focus only on subjects taking calcium; no collapsing is necessary. Effects will be summarized using risk ratios rather than odds ratios to improve interpretation. Two-sided significance levels of 0.05 will be taken to denote statistical significance.

As in our previous trials, subsequent, more detailed, multivariate analyses will consist of generalized linear models for binary data to estimate adjusted risk ratios (i.e., using a log link function) and confidence intervals for neoplastic polyp occurrence among the treatment groups. We anticipate that the distribution of relevant covariables will be approximately balanced across the treatment groups because of random assignment in a relatively large sample. Some imbalances may occur, making it necessary to critically examine possible covariates. These include number of previous adenomas, size of qualifying adenomas, selected dietary variables (e.g., calcium intake) or sunlight exposure. The distribution by treatment group of these covariates (for the whole sample as well as by center) will be assessed. For those variables with large and systematic imbalances, stratified analyses will be employed to confirm or modify the preliminary analyses. Factors used to stratify the randomization (center, planned follow-up interval, full factorial participation) will be included as covariates. We will also use these models to evaluate the interaction between vitamin D supplementation and calcium supplementation. The interaction effect will be tested by including the appropriate product term in the model. This interaction analysis will include only those subjects enrolled in the full factorial design (i.e., women wishing to take calcium supplements will be excluded). We do not expect differential treatment effects according to age, gender or race, but we will consider the corresponding interactions for the treatments.

Analyses pertaining to the secondary hypotheses will use methods similar to those described for the primary hypotheses. The comparison of calcium + vitamin D versus vitamin D alone (hypotheses 4) will be dealt with in the same manner as the primary hypotheses. Comparison of treatment effects within subgroups defined by VDR genotype or baseline vitamin D level will initially involve contingency tables stratified by genotype or vitamin D level. We will then apply generalized linear models with appropriate interaction terms and other covariates. If a possible (i.e., p < 0.10) interaction is observed, we will focus on effects within strata of the effect-modifying factor. In some analyses we expect to be able to consider the genotype data as a 3-level variable (homozygous wild type, heterozygous, homozygous variant) since the prevalence of the least common of these (homozygote variant) is approximately 15% for both the polymorphisms used. Some analyses, however, will require grouping the heterozygous subjects with those who are homozygous variant.

In addition to the planned treatment comparisons, we will conduct important subgroup analyses. For example, we will assess the treatment effects according to self-reported NSAID use during the trial. We will group subjects as having low, medium and high NSAID use during the study and add this exposure to our regression models and assess its main effect and its modification of the vitamin D and calcium risk ratios. In similar analyses, we will assess the impact of baseline vitamin D levels and VDR polymorphisms as main effects and as modifiers of the intervention effects.

We will also conduct analyses regarding the number and characteristics of polyps. The number of adenomas per patient will be evaluated using contingency tables (with groupings such as 0, 1, 2+ adenomas) as well as multivariate Poisson regression with the counts themselves as the dependent variable. The size of neoplastic polyps will be incorporated using various dichotomous endpoints; if sufficient adenomas at least a cm in diameter are found, we will use that cut point. Adenomas with various histological characteristics (serrated, tubulovillous/villous, advanced dysplasia/cancer) will also be considered as dichotomous endpoints as outlined above for all adenomas; in particular, we will assess effects on “advanced histology adenomas” (villous component, advanced dysplasia) or “advanced adenomas (advanced histology or 1+ cm. in diameter).

Analyses regarding the main hypotheses will not be possible until all subjects have completed follow-up, after the period covered by this application. We do not anticipate stopping this trial early for efficacy, since early termination biases estimates of effect,[^189^](#_ENREF_189) and in this population under colonoscopic surveillance, there is little likelihood of harm from continuing until the planned study completion. However, for toxicity, we anticipate discussing with the Safety and Data Monitoring Committee the adoption of stopping guidelines that account for multiple analyses over time (e.g., using O’Brien-Fleming boundaries).[^190^](#_ENREF_190) Such guidelines have been established in our Aspirin/Folate Polyp Prevention Study to consider possible treatment effects on mortality and coronary heart disease.

### 13.6 Assumptions

Only standard assumptions for statistical analysis will be made. These include: (1) that the study sample is representative of the eligible patient population; (2) the protocol procedures for treatment and follow-up of individual patients will result in an unbiased estimate of the treatment effect; (3) regression models used for the multivariate analyses appropriately fit the empiric data. Because this study uses a “gold-standard” approach in its design, namely with random treatment assignment and double blinding of treatment assignment, the possibility of systematic bias is minimal.

### 13.7 Compliance and missing data

Missing data may arise due to patients missing follow-up colonoscopies. In past studies by the Polyp Prevention Group, the percent of missing exams has been small. In addition, because the treatment assignment is random, and both subjects and investigators are blinded to treatment assignments, it is not likely that missing exams will generate a bias. Patients with missing follow-up data will be excluded from the statistical analysis of the main endpoints.

### 13.8 Interim analyses

No formal interim statistical analysis is planned for this study. The study will not stop early for efficacy. The study will be monitored by an independent Safety and Data Monitoring Committee. The Project Coordination Center will provide any data analysis reports the committee requires.

### 13.9 Ancillary analyses

Aside from posited effects on colon carcinogenesis, calcium and vitamin D may have a modest protective effect on cardiovascular disease risk and on fractures. In our routine follow-up, we will monitor hospital admissions for all cardiovascular events and will thus be able to compare the event rates in subjects treated with calcium with those given placebo. However, small numbers of events are expected and our power to detect meaningful differences is likely to be low. We will also regularly ask our participants to report fractures they have sustained after randomization. As in our previous calcium trial, we will carefully follow all subjects for cancer at other sites.

***14.0 Ethical and regulatory considerations***

*14.1 Form FDA 1572*

The principal investigator at each Study Center will provide a signed Form 1572 stating that the study will be conducted in compliance with regulations for clinical investigations as required by FDA regulations.

*14.2 IRB Approval*

Prior to initiating the study at any of the participating Study Centers the Principal Investigator at that center will obtain written approval to conduct the study from the appropriate IRB. In addition, IRB approval will be required from any recruitment sites where subjects are recruited that are not covered by the Study Center IRB. Should changes to the study protocol become necessary, protocol amendments will be promptly submitted in writing to the IRBs and approval received prior to implementation. Dates of IRB submission(s) and approval(s) and copies of the approved documents will be submitted to the Project Coordination Center for tracking and quality control purposes.

*14.2.1 Potential Risks to Subjects and Protections Against Risks*

The study’s physical risks are those associated with the study agents. Overconsumption of calcium and Vitamin D may cause conditions such as hypercalcemia, kidney stones, and renal insufficiency, which are potentially serious. Calcium supplements may interact with some commonly used drugs. Serious drug interactions between the study tablet and other medications are unlikely, but possible. In most cases this interaction can be minimized by staggering the administration of the drug. The likelihood of these complications is very small given the low doses of the study agents used and the many safety procedures used to protect against this risk. These protections will include:

- excluding from participation in the trial those individuals that are at increased risk for these conditions based on their medical history and baseline measurements of blood levels of calcium, creatinine and vitamin D.
- educating subjects about the symptoms of hypercalcemia.
- monitoring subjects at six month intervals for any adverse symptoms, medical diagnoses and hospitalizations, or excess dietary or supplemental intake of calcium and vitamin D; and discontinuation of study medications when indicated due to symptoms, adverse clinical events, or excess consumption of these agents outside of the study
- asking participants to report all medications being taken during study participation and advising them to consult with their physician about staggering the administration of current drugs or new medications that are begun during study participation.
- measuring calcium, creatinine and/or vitamin D levels approximately one year and, where applicable, three years after randomization; patients who have developed hypercalcemia, renal insufficiency or very high levels of 25-OH vitamin D (> 100 ng/ml) will be withdrawn from treatment.
- reviewing adverse events by blinded (and if necessary unblinded) treatment groups by the Safety and Data Monitoring Committee; this Committee will have overall responsibility for interpreting data on adverse events and recommending actions to ensure that subjects are not exposed to undue risks.

A physical risk that may be associated with insufficient intake of calcium or vitamin D over the long term is an increased risk for osteoporosis, a condition in which increased bone fragility can lead to fractures. This risk may be greater among post-menopausal women who undergo accelerated bone loss around the time of the menopause. The risk may be increased if subjects randomized to placebo reduce their dietary intake of calcium or vitamin D or stop taking calcium- or vitamin D-containing dietary supplements as a result of their participation in this trial. To protect against this risk:

- - Female subjects will be offered the option of being randomized to calcium alone or to calcium plus vitamin D. These women will receive 1200 mg of elemental calcium daily in their study pills.
  - Subjects will be cautioned NOT to reduce their dietary intake of calcium or vitamin D containing foods during their participation in this trial UNLESS we alert them that their intake is too high. Dietary intake will be assessed with a brief dietary screener at enrollment and randomization, and at six month intervals after randomization. Subjects will be counseled that dietary intake of calcium and vitamin D equivalent to about one quart of milk (about 1200 mg elemental calcium and 400 IU vitamin D) daily is acceptable for participation in this trial.
  - The levels of 25-(OH)-vitamin D in the blood will measured at approximately one year and, where applicable, three years after randomization and at the end of treatment. Subjects who have developed low levels of 25-(OH)-vitamin D (<10ng/ml) will be notified and withdrawn from treatment so that they can take dietary supplements.

Another physical risk is that associated with the study blood draws. The blood drawing procedure requires that a needle be inserted into an arm vein and blood withdrawn. Almost all donors experience slight pain at the site of the needle insertion and some may develop a small bruise. The risk of this discomfort from the blood draw is likely but not serious.

Social risks could occur if confidentiality is violated. Dissemination of information obtained from research, including genetic analyses, has the potential of adversely affecting employability or insurability.

To protect subject confidentiality, the following steps will be taken:

- Each subject will be assigned a study identification number and a two letter study identification code (consisting of the first letter of the subject’s first name and the last letter of the subject’s last name). All data transfers regarding a subject will be labeled only with these identification codes.
- The main clinical trial database, which is maintained by the Dartmouth Project Coordination Center, will contain only the data elements allowed in a ‘limited data set’ as defined by HIPAA, with the exception of the pathology slide number, which will be used solely to identify and track receipt and review of these slides by the study pathologist and their return to the pathology lab of origin.
- The pharmacy database, which is used for the purpose of pill shipments by the Dartmouth Project Coordination Center and which contains only the participants names, addresses and telephone numbers, will be kept separate from the main clinical trial database and will not be directly linked to any other information collected about the participant during this study. This data will also be used for contacting some participants for the two extra interviews to evaluate our study methods (e.g. the assessment of blinding effectiveness) and for mailing Cold and Flu Questionnaires. Finally, study-wide communications (e.g., the Study Newsletters) may also be sent by pharmacy personnel from the Project Coordination Center using this database.
- Medical record forms (endoscopy and pathology reports) will be submitted to the Project Coordination Center labeled only with coded study IDs.
- Blood samples sent to the Dartmouth Hitchcock Medical Center laboratory will be labeled and tracked only with unique barcoded numbers.
- Pathology slides generated during routine clinical care are generally labeled only with a local pathology ID number, however, in some cases other identifying information such as subject names may also be included as per the local pathology laboratory standard of practice. Slides sent to the Dartmouth Project Coordination Center and to the Study Pathologist will be tracked only with the pathology number. These slides will be returned to the laboratory of origin once they are read by the Study Pathologist, unless the subject consents to their storage for future research and permission is obtained from pathology laboratory of origin.
- All computer systems and programs will be equipped with password protection.
- All network-based study communications will be encrypted.
- All printed study materials at the Study Centers and at the Dartmouth Project Coordination Center that contain identifying information, such as name, address, telephone number and social security numbers, will be stored in locked filing cabinets.
- All study personnel will be trained to respect and protect subject confidentiality; information regarding subjects will be discussed only with other study personnel
- We have obtained a Certificate of Confidentiality from the NIH to protect the genetic information generated by this study

As well, the seriousness of this risk is allayed by the fact that the type of genetic analyses to be performed in this study are very unlikely to yield any clinically significant information that could be used to predict future health status.

A potential psychological risk could occur if patients feel a sense of coercion to participate in the trial. The likelihood is small, since patients will be assured in the informed consent discussion during the intake process that participation is purely voluntary, and that they may decline to participate or withdraw from the study at any time with no impact on their regular medical care.

*14.2.2 Potential Benefits*

Should calcium or vitamin D prove effective in preventing cancer, the public will clearly benefit. The relative lack of toxicity and simplicity of administration of these agents almost certainly will make them acceptable to large numbers of asymptomatic people. Such a preventive strategy is likely to be more acceptable and effective than recommendations to avoid life style habits that may only modestly increase risk. The potential benefit of a simple, safe, and inexpensive prophylactic is clear. The actual risks to individuals appear to be minimal while the potential benefit to the public (and possibly to the study subjects themselves) is great, thus the risk:benefit ratio favors conducting this study.

The proposed research will have both public health and scientific importance. It has the potential to confirm other experimental and observational studies indicating that calcium and vitamin D supplementation protect against colorectal neoplasms. It will show, for the first time, whether both agents together are more effective than calcium alone. From a public health perspective, a positive finding will suggest a low-cost strategy for reducing the burden of colorectal cancer in the general population. From a scientific perspective, the study will permit investigation of the mechanisms underlying the beneficial effects of the study agents, and thus elucidate the pathogenesis of cancer in the large bowel.

*14.3 Informed Consent*

The informed consent process will begin during the first patient contact and continue throughout the study. The Principal Investigator at each Study Center may delegate the responsibility of obtaining informed consent to the center’s study coordinator. During the initial in-person intake appointment, the study coordinator will conduct an informed consent discussion, focusing on the research nature of the study, its purposes, expected duration of participation, study procedures including randomization to active agent or placebo, possible risks or discomforts, possible benefits, and confidentiality. It will stress the voluntary nature of participation and the right to withdraw. Willing patients will document their consent to participate by signing and dating an informed consent document presenting this information in written form and providing authorization to access personal health information for HIPAA compliance. The discussion will take place at the Study Center in a private, comfortable environment free of interruptions and with sufficient time allocated to address all questions and concerns. Subjects are given a copy of the signed and dated informed consent documents; the original signed and dated consents are placed in the subject’s study file, and a copy is also placed in the medical record.

*14.4 Data and Safety Monitoring Plan*

A Data and Safety Monitoring Committee will regularly review interim data to assess compliance, monitor toxicity, and recommend whether the trial should continue. Committee members are independent experts chosen on the basis of their expertise and scientific rigor. They are not associated with the trial or with the pharmaceutical companies that supply the study agents. The Principal Investigator, together with the study’s Steering Committee, carefully assess the background of each member to rule out the possibility of conflicts of interest with or financial interests in the research outcome. Committee members’ areas of expertise span the disciplines relevant to the conduct of GI clinical trials, including epidemiology, clinical trials, pharmacology, biostatistics, and clinical care of adenoma patients.

The Committee will confer at six-month intervals, generally by telephone conference call, with in-person meetings, as needed. The study’s Principal Investigator, John Baron, MD, and the study’s Biostatistician as well as other Project Coordination Center investigators as needed, will participate in the meetings’ open sessions. If needed, the Committee’s voting members may then choose to discuss in closed session unblinded data or other material that should be kept confidential from the investigators. Under the supervision of the study Biostatistician, the lead programmer at the Project Coordination Center will prepare a bi-annual “Report to the Data and Safety Monitoring Committee” which includes interim outcome data. The study Biostatistician and programmer ensure that the full report is kept strictly confidential.

The Committee will have the responsibility to review the research protocol and to evaluate the progress of the trial overall and at each participating Study Center. This will include accrual, compliance with pill-taking and study exams, use of calcium and Vitamin D supplements, prescription drug use, mortality, episodes of hospitalization, toxicities, adverse events, disease diagnoses, reported changes in health, protocol violations and data quality. Serious unexpected events will be disclosed to the committee in between meetings. The Committee will also review interim endpoint data. The Committee will evaluate participant risk vs benefit as the trial progresses, considering evolving scientific discoveries or treatment options that may affect the desirability of continued treatment. At the conclusion of each meeting, the Committee will recommend whether the trial be continued. Following each meeting of the Data and Safety Monitoring Committee, its secretary will prepare a report on the questions raised by Committee members, monitoring recommendations, and recommendations for the continuation of the trial. This report will be distributed confidentially to meeting participants. The Committee’s secretary will also prepare a redacted summary of this report, focusing on safety issues, for distribution to Study Center co-investigators and their IRBs.

*14.5 Project Coordination Center, Sponsor or FDA monitoring*

The Dartmouth Project Coordination Center, the FDA and the NCI or their designees may monitor/audit various aspects of the study. These monitors will be given access to facilities, supplies and records to review and verify data pertinent to this study.

*14.6 Record Retention*

Clinical records for all subjects studied will be maintained by the Investigator in a secure storage facility and stored until the Dartmouth Project Coordination Center directs the material to be destroyed. Once study enrollment is complete and summary statistics have been analyzed, the Project Coordination Center will notify all Study Centers that they may destroy all data collected from screened subjects who were not subsequently enrolled into the study.

*14.7 Certificate of Confidentiality*

To help protect subject privacy, we have obtained a "Certificate of Confidentiality" from the National Institutes of Health. With this Certificate we cannot be forced to disclose information that may identify a subject, even by a court subpoena, in any federal, state, or local civil, criminal, administrative, legislative, or other proceedings. Although the Certificate adds another layer of protection, it does not apply in the following situations: 1) When we receive requests for information that come from the United States Government for the purpose of evaluating or auditing research projects funded by the Federal Government, or for information needed to meet the requirements of the Food and Drug Administration (FDA); and 2) If a subject chooses to give written consent to release research information to an insurer, employer, or other person the Certificate does not allow us to withhold that information.

***15.0 References***

1. Landis SH, Murray T, Bolden S, Wingo PA. Cancer Statistics. CA Cancer J Clin 1998;48:6-29.

2. Reichel H, Koeffler P, Norman A. The role of the vitamin D endocrine system in health and disease. N Engl J Med 1989;1989:980-91.

3. Fraser DR. Vitamin D Lancet 1995;345:104-7.

4. Lau KH, Baylink DJ. Vitamin D therapy of osteoporosis: plain vitamin D therapy versus active vitamin D analog (D-hormone) therapy. Calcified Tissue International 1999;65:295-306.

5. Holick M. The use and interpretation of assays for vitamin D and its metabolites. J Nutr 1990;120:1464-9.

6. Brown AJ, Dusso A, Slatopolsky E. Vitamin D. American Journal of Physiology 1999;277:F157-75.

7. Bell NH. Renal and nonrenal 25-hydroxyvitamin D-1alpha-hydroxylases and their clinical significance. Journal of Bone & Mineral Research 1998;13:350-3.

8. Institute of Medicine. Standing Committee on the Scientific Evaluation of Dietary Reference Intakes. Food and Nutrition Board. In: Dietary Reference Intakes for Calcium, Phosphorus, Magnesium, Vitamin D and Fluoride. Washington, D.C.: National Academy Press; 1997:432.

9. Barger-Lux MJ, Heaney RP, Lanspa SJ, Healy JC, DeLuca HF. An investigation of sources of variation in calcium absorption efficiency. Journal of Clinical Endocrinology & Metabolism 1995;80:406-11.

10. Heaney RP, Barger-Lux MJ, Dowell MS, Chen TC, Holick MF. Calcium absorptive effects of vitamin D and its major metabolites. Journal of Clinical Endocrinology & Metabolism 1997;82:4111-6.

11. Holick MF. Vitamin D requirements for humans of all ages: new increased requirements for women and men 50 years and older. Osteoporosis International 1998;8:S24-9.

12. Holick MF. Sunlight "D"ilemma: risk of skin cancer or bone disease and muscle weakness. Lancet 2001;357:4-6.

13. Norman AW, Song X, Zanello L, Bula C, Okamura WH. Rapid and genomic biological responses are mediated by different shapes of the agonist steroid hormone, 1alpha,25(OH)2vitamin D3. Steroids 1999;64:120-8.

14. Curran JE, Vaughan T, Lea RA, Weinstein SR, Morrison NA, Griffiths LR. Association of A vitamin D receptor polymorphism with sporadic breast cancer development. International Journal of Cancer 1999;83:723-6.

15. Dunning AM, McBride S, Gregory J, et al. No association between androgen or vitamin D receptor gene polymorphisms and risk of breast cancer. Carcinogenesis 1999;20:2131-5.

16. Ingles SA, Ross RK, Yu MC, et al. Association of prostate cancer risk with genetic polymorphisms in vitamin D receptor and androgen receptor Journal of the National Cancer Institute 1997;89:166-70.

17. Carling T, Ridefelt P, Hellman P, Rastad J, Akerstrom G. Vitamin D receptor polymorphisms correlate to parathyroid cell function in primary hyperparathyroidism. Journal of Clinical Endocrinology & Metabolism 1997;82:1772-5.

18. Ingles SA, Haile RW, Henderson BE, et al. Strength of linkage disequilibrium between two vitamin D receptor markers in five ethnic groups: implications for association studies. Cancer Epidemiology, Biomarkers & Prevention 1997;6:93-8.

19. Ingles SA, Garcia DG, Wang W, et al. Vitamin D receptor genotype and breast cancer in Latinas (United States). Cancer Causes & Control 2000;11:25-30.

20. Ma J, Stampfer MJ, Gann PH, et al. Vitamin D receptor polymorphisms, circulating vitamin D metabolites, and risk of prostate cancer in United States physicians. Cancer Epidemiology, Biomarkers & Prevention 1998;7:385-90.

21. Wishart JM, Horowitz M, Need AG, et al. Relations between calcium intake, calcitriol, polymorphisms of the vitamin D receptor gene, and calcium absorption in premenopausal women. American Journal of Clinical Nutrition 1997;65:798-802.

22. Dawson-Hughes B, Harris SS, Finneran S. Calcium absorption on high and low calcium intakes in relation to vitamin D receptor genotype. Journal of Clinical Endocrinology & Metabolism 1995;80:3657-61.

23. Campbell MJ, Koeffler HP. Toward therapeutic intervention of cancer by vitamin D compounds Journal of the National Cancer Institute 1997;89:182-5.

24. Cross HS, Pavelka M, Slavik J, Peterlik M. Growth control of human colon cancer cells by vitamin D and calcium in vitro. Journal of the National Cancer Institute 1992;84:1355-7.

25. Lointier P, Wargovich MJ, Saez S, Levin B, Wildrick DM, Boman BM. The role of vitamin D3 in the proliferation of a human colon cancer cell line in vitro. Anticancer Research 1987;7:817-21.

26. James SY, Mackay AG, Colston KW. Effects of 1,25 dihydroxyvitamin D3 and its analogues on induction of apoptosis in breast cancer cells. Journal of Steroid Biochemistry & Molecular Biology 1996;58:395-401.

27. Diaz GD, Paraskeva C, Thomas MG, Binderup L, Hague A. Apoptosis is induced by the active metabolite of vitamin D3 and its analogue EB1089 in colorectal adenoma and carcinoma cells: possible implications for prevention and therapy. Cancer Research 2000;60:2304-12.

28. Holick MF. 1,25-Dihydroxyvitamin D3 and the skin: a unique application for the treatment of psoriasis. Proceedings of the Society for Experimental Biology & Medicine 1989;191:246-57.

29. Holick MF. Noncalcemic actions of 1,25-dihydroxyvitamin D3 and clinical applications. Bone 1995;17:107S-11S.

30. Menne T, Larsen K. Psoriasis treatment with vitamin D derivatives. Seminars in Dermatology 1992;11:278-83.

31. Park BS, Park JS, Lee DY, Youn JI, Kim IG. Vitamin D receptor polymorphism is associated with psoriasis. Journal of Investigative Dermatology 1999;112:113-6.

32. Thomas MG, Tebbutt S, Williamson RC. Vitamin D and its metabolites inhibit cell proliferation in human rectal mucosa and a colon cancer cell line. Gut 1992;33:1660-3.

33. Thomas MG, Nugent KP, Forbes A, Williamson RC. Calcipotriol inhibits rectal epithelial cell proliferation in ulcerative proctocolitis. Gut 1994;35:1718-20.

34. Sadava D, Remer T, Petersen K. Hyperplasia, hyperproliferation and decreased migration rate of colonic epithelial cells in mice fed a diet deficient in vitamin D. Biology of the Cell 1996;87:113-5.

35. Hashiba H, Fukushima M, Chida K, Kuroki T. Systemic inhibition of tumor promoter-induced ornithine decarboxylase in 1 alpha-hydroxyvitamin D3-treated animals. Cancer Research 1987;47:5031-5.

36. Belleli A, Shany S, Levy J, Guberman R, Lamprecht SA. A protective role of 1,25-dihydroxyvitamin D3 in chemically induced rat colon carcinogenesis. Carcinogenesis 1992;13:2293-8.

37. Meggouh F, Lointier P, Saez S. Sex steroid and 1,25-dihydroxyvitamin D3 receptors in human colorectal adenocarcinoma and normal mucosa. Cancer Research 1991;51:1227-33.

38. Vandewalle B, Adenis A, Hornez L, Revillion F, Lefebvre J. 1,25-dihydroxyvitamin D3 receptors in normal and malignant human colorectal tissues. Cancer Letters 1994;86:67-73.

39. Shabahang M, Buras RR, Davoodi F, Schumaker LM, Nauta RJ, Evans SR. 1,25-Dihydroxyvitamin D3 receptor as a marker of human colon carcinoma cell line differentiation and growth inhibition. Cancer Research 1993;53:3712-8.

40. Evans SR, Nolla J, Hanfelt J, Shabahang M, Nauta RJ, Shchepotin IB. Vitamin D receptor expression as a predictive marker of biological behavior in human colorectal cancer. Clinical Cancer Research 1998;4:1591-5.

41. Holt PR, Arber N, Halmos B, et al. Colonic epithelial cell proliferation decreases with increasing levels of serum 25-hydroxy vitamin D. Cancer Epidemiology, Biomarkers & Prevention 2002;11:113-9.

42. Eisman JA, Barkla DH, Tutton PJ. Suppression of in vivo growth of human cancer solid tumor xenografts by 1,25-dihydroxyvitamin D3. Cancer Research 1987;47:21-5.

43. Sitrin MD, Halline AG, Abrahams C, Brasitus TA. Dietary calcium and vitamin D modulate 1,2-dimethylhydrazine-induced colonic carcinogenesis in the rat. Cancer Research 1991;51:5608-13.

44. Huerta S, Irwin RW, Heber D, et al. 1alpha,25-(OH)(2)-D(3) and its synthetic analogue decrease tumor load in the Apc(min) Mouse. Cancer Research 2002;62:741-6.

45. Kawaura A, Tanida N, Sawada K, Oda M, Shimoyama T. Supplemental administration of 1 alpha-hydroxyvitamin D3 inhibits promotion by intrarectal instillation of lithocholic acid in N-methyl-N-nitrosourea-induced colonic tumorigenesis in rats. Carcinogenesis 1989;10:647-9.

46. Beaty MM, Lee EY, Glauert HP. Influence of dietary calcium and vitamin D on colon epithelial cell proliferation and 1,2-dimethylhydrazine-induced colon carcinogenesis in rats fed high fat diets. Journal of Nutrition 1993;123:144-52.

47. Evans SR, Shchepotin EI, Young H, Rochon J, Uskokovic M, Shchepotin IB. 1,25-dihydroxyvitamin D3 synthetic analogs inhibit spontaneous metastases in a 1,2-dimethylhydrazine-induced colon carcinogenesis model. International Journal of Oncology 2000;16:1249-54.

48. Comer PF, Clark TD, Glauert HP. Effect of dietary vitamin D3 (cholecalciferol) on colon carcinogenesis induced by 1,2-dimethylhydrazine in male Fischer 344 rats. Nutrition & Cancer 1993;19:113-24.

49. van den Bemd GJ, Pols HA, van Leeuwen JP. Anti-tumor effects of 1,25-dihydroxyvitamin D3 and vitamin D analogs. Current Pharmaceutical Design 2000;6:717-32.

50. Haussler MR, Whitfield GK, Haussler CA, et al. The nuclear vitamin D receptor: biological and molecular regulatory properties revealed. Journal of Bone & Mineral Research 1998;13:325-49.

51. Moffatt KA, Johannes WU, Hedlund TE, Miller GJ. Growth inhibitory effects of 1alpha, 25-dihydroxyvitamin D(3) are mediated by increased levels of p21 in the prostatic carcinoma cell line ALVA-31. Cancer Research 2001;61:7122-9.

52. Tong WM, Kallay E, Hofer H, et al. Growth regulation of human colon cancer cells by epidermal growth factor and 1,25-dihydroxyvitamin D3 is mediated by mutual modulation of receptor expression. European Journal of Cancer 1998;34:2119-25.

53. Tong WM, Hofer H, Ellinger A, Peterlik M, Cross HS. Mechanism of antimitogenic action of vitamin D in human colon carcinoma cells: relevance for suppression of epidermal growth factor-stimulated cell growth. Oncology Research 1999;11:77-84.

54. Xie SP, Pirianov G, Colston KW. Vitamin D analogues suppress IGF-I signalling and promote apoptosis in breast cancer cells. European Journal of Cancer 1999;35:1717-23.

55. Salim EI, Wanibuchi H, Taniyama T, et al. Inhibition of development of N,N'-dimethylhydrazine-induced rat colonic aberrant crypt foci by pre, post and simultaneous treatments with 24R,25-dihydroxyvitamin D3. Japanese Journal of Cancer Research 1997;88:1052-62.

56. Makishima M, Lu TT, Xie W, et al. Vitamin D receptor as an intestinal bile acid sensor. Science 2002;296:1313-6.

57. DeLuca HF, Zierold C. Mechanisms and functions of vitamin D. Nutrition Reviews 1998;56:S4-10; discussion S 54-75.

58. Lemire J. 1,25-Dihydroxyvitamin D3--a hormone with immunomodulatory properties. Zeitschrift fur Rheumatologie 2000;59:24-7.

59. Garland CF, Garland FC. Do sunlight and vitamin D reduce the likelihood of colon cancer? International Journal of Epidemiology 1980;9:227-31.

60. Emerson JC, Weiss NS. Colorectal cancer and solar radiation. Cancer Causes & Control 1992;3:95-9.

61. Benito E, Stiggelbout A, Bosch FX, et al. Nutritional factors in colorectal cancer risk: a case-control study in Majorca. International Journal of Cancer 1991;49:161-7.

62. Ferraroni M, La Vecchia C, D'Avanzo B, Negri E, Franceschi S, Decarli A. Selected micronutrient intake and the risk of colorectal cancer. British Journal of Cancer 1994;70:1150-5.

63. Pritchard RS, Baron JA, Gerhardsson de Verdier M. Dietary calcium, vitamin D, and the risk of colorectal cancer in Stockholm, Sweden. Cancer Epidemiology, Biomarkers & Prevention 1996;5:897-900.

64. Kampman E, Slattery ML, Caan B, Potter JD. Calcium, vitamin D, sunshine exposure, dairy products and colon cancer risk (United States). Cancer Causes & Control 2000;11:459-66.

65. Olsen J, Kronborg O, Lynggaard J, Ewertz M. Dietary risk factors for cancer and adenomas of the large intestine. A case-control study within a screening trial in Denmark. European Journal of Cancer 1994;30A:53-60.

66. Marcus PM, Newcomb PA. The association of calcium and vitamin D, and colon and rectal cancer in Wisconsin women. International Journal of Epidemiology 1998;27:788-93.

67. Breuer-Katschinski B, Nemes K, Marr A, et al. Colorectal adenomas and diet: a case-control study. Colorectal Adenoma Study Group. Digestive Diseases & Sciences 2001;46:86-95.

68. Peters RK, Pike MC, Garabrant D, Mack TM. Diet and colon cancer in Los Angeles County, California. Cancer Causes & Control 1992;3:457-73.

69. Boutron MC, Faivre J, Marteau P, Couillault C, Senesse P, Quipourt V. Calcium, phosphorus, vitamin D, dairy products and colorectal carcinogenesis: a French case--control study British Journal of Cancer 1996;74:145-51.

70. Levine AJ, Harper JM, Ervin CM, et al. Serum 25-hydroxyvitamin D, dietary calcium intake, and distal colorectal adenoma risk. Nutrition & Cancer 2001;39:35-41.

71. Benito E, Cabeza E, Moreno V, Obrador A, Bosch FX. Diet and colorectal adenomas: a case-control study in Majorca. International Journal of Cancer 1993;55:213-9.

72. Little J, Logan RF, Hawtin PG, Hardcastle JD, Turner ID. Colorectal adenomas and diet: a case-control study of subjects participating in the Nottingham faecal occult blood screening programme. British Journal of Cancer 1993;67:177-84.

73. Levi F, Pasche C, Lucchini F, La Vecchia C. Selected micronutrients and colorectal cancer. a case-control study from the canton of Vaud, Switzerland. European Journal of Cancer 2000;36:2115-9.

74. Zheng W, Anderson KE, Kushi LH, et al. A prospective cohort study of intake of calcium, vitamin D, and other micronutrients in relation to incidence of rectal cancer among postmenopausal women. Cancer Epidemiology, Biomarkers & Prevention 1998;7:221-5.

75. Martinez M, Giovannucci E, Colditz G, et al. Calcium, vitamin D, and the occurrence of colorectal cancer among women. Journal of the National Cancer Institute 1996;88:1375-82.

76. Kearney J, Giovannucci E, Rimm EB, et al. Calcium, vitamin D, and dairy foods and the occurrence of colon cancer in men. American Journal of Epidemiology 1996;143:907-17.

77. Bostick RM, Potter JD, Sellers TA, McKenzie DR, Kushi LH, Folsom AR. Relation of calcium, vitamin D, and dairy food intake to incidence of colon cancer among older women. The Iowa Women's Health Study. American Journal of Epidemiology 1993;137:1302-17.

78. Garland C, Shekelle RB, Barrett-Connor E, Criqui MH, Rossof AH, Paul O. Dietary vitamin D and calcium and risk of colorectal cancer: a 19-year prospective study in men. Lancet 1985;1:307-9.

79. Jarvinen R, Knekt P, T. H, Aromaa A. Prospective study on milk products, calcium and cancers of the colon and rectum. European Journal of Clinical Nutrition 2000;55:1000-7.

80. Garland C, Comstock G, Garland F, Helsing K, Shaw E, Gorham E. Serum 35-hydroxyvitamin D and colon cancer: eight year prospective study. Lancet 1989;ii:1176-9.

81. Braun MM, Helzlsouer KJ, Hollis BW, Comstock GW. Colon cancer and serum vitamin D metabolite levels 10-17 years prior to diagnosis. American Journal of Epidemiology 1995;142:608-11.

82. Tangrea J, Helzlsouer K, Pietinen P, et al. Serum levels of vitamin D metabolites and the subsequent risk of colon and rectal cancer in Finnish men. Cancer Causes & Control 1997;8:615-25.

83. Peters U, McGlynn KA, Chatterjee N, et al. Vitamin d, calcium, and vitamin d receptor polymorphism in colorectal adenomas. Cancer Epidemiology, Biomarkers & Prevention 2001;10:1267-74.

84. Slattery ML, Yakumo K, Hoffman M, Neuhausen S. Variants of the VDR gene and risk of colon cancer (United States). Cancer Causes & Control 2001;12:359-64.

85. Ingles SA, Wang J, Coetzee GA, Lee ER, Frankl HD, Haile RW. Vitamin D receptor polymorphisms and risk of colorectal adenomas (United States). Cancer Causes & Control 2001;12:607-14.

86. Kim HS, Newcomb PA, Ulrich CM, et al. Vitamin D receptor polymorphism and the risk of colorectal adenomas: evidence of interaction with dietary vitamin D and calcium. Cancer Epidemiology, Biomarkers & Prevention 2001;10:869-74.

87. Tangpricha V, Flanagan JN, Whitlatch LW, et al. 25-hydroxyvitamin D-1alpha-hydroxylase in normal and malignant colon tissue. Lancet 2001;357:1673-4.

88. Cross HS, Bareis P, Hofer H, et al. 25-Hydroxyvitamin D(3)-1alpha-hydroxylase and vitamin D receptor gene expression in human colonic mucosa is elevated during early cancerogenesis. Steroids 2001;66:287-92.

89. Cross HS, Hulla W, Tong WM, Peterlik M. Growth regulation of human colon adenocarcinoma-derived cells by calcium, vitamin D and epidermal growth factor. Journal of Nutrition 1995;125:2004S-8S.

90. Cross HS, Peterlik M, Reddy GS, Schuster I. Vitamin D metabolism in human colon adenocarcinoma-derived Caco-2 cells: expression of 25-hydroxyvitamin D3-1alpha-hydroxylase activity and regulation of side-chain metabolism. Journal of Steroid Biochemistry & Molecular Biology 1997;62:21-8.

91. Schwartz GG, Whitlatch LW, Chen TC, Lokeshwar BL, Holick MF. Human prostate cells synthesize 1,25-dihydroxyvitamin D3 from 25-hydroxyvitamin D3. Cancer Epidemiology, Biomarkers & Prevention 1998;7:391-5.

92. Barreto AM, Schwartz GG, Woodruff R, Cramer SD. 25-Hydroxyvitamin D3, the prohormone of 1,25-dihydroxyvitamin D3, inhibits the proliferation of primary prostatic epithelial cells. Cancer Epidemiology, Biomarkers & Prevention 2000;9:265-70.

93. Chen TC, Schwartz GG, Burnstein KL, Lokeshwar BL, Holick MF. The in vitro evaluation of 25-hydroxyvitamin D3 and 19-nor-1alpha,25-dihydroxyvitamin D2 as therapeutic agents for prostate cancer. Clinical Cancer Research 2000;6:901-8.

94. Institute of Medicine. Dietary reference intakes for calcium and vitamin D. Washington, DC: National Academies Press, 2011.

95. van der Wielen RP, Lowik MR, van den Berg H, et al. Serum vitamin D concentrations among elderly people in Europe. Lancet 1995;346:207-10.

96. Vieth R. Vitamin D supplementation, 25-hydroxyvitamin D concentrations, and safety American Journal of Clinical Nutrition 1999;69:842-56.

97. Chapuy MC, Preziosi P, Maamer M, et al. Prevalence of vitamin D insufficiency in an adult normal population. Osteoporosis International 1997;7:439-43.

98. Malabanan A, Veronikis IE, Holick MF. Redefining vitamin D insufficiency Lancet 1998;351:805-6.

99. Gloth FM, 3rd, Tobin JD. Vitamin D deficiency in older people. Journal of the American Geriatrics Society 1995;43:822-8.

100. Byrne PM, Freaney R, McKenna MJ. Vitamin D supplementation in the elderly: review of safety and effectiveness of different regimes. Calcified Tissue International 1995;56:518-20.

101. Harris SS, Dawson-Hughes B. Plasma Vitamin D and 25OHD responses of young and old men to supplementation with Vitamin D3. Journal of the American College of Nutrition 2002;21:357-62.

102. Chapuy MC, Chapuy P, Thomas JL, Hazard MC, Meunier PJ. Biochemical effects of calcium and vitamin D supplementation in elderly, institutionalized, vitamin D-deficient patients. Revue du Rhumatisme (English Edition) 1996;63:135-40.

103. Sebert JL, Garabedian M, Chauvenet M, Maamer M, Agbomson F, Brazier M. Evaluation of a new solid formulation of calcium and vitamin D in institutionalized elderly subjects. A randomized comparative trial versus separate administration of both constituents. Revue Du Rhumatisme, English Edition 1995;62:288-94.

104. Himmelstein S, Clemens TL, Rubin A, Lindsay R. Vitamin D supplementation in elderly nursing home residents increases 25(OH)D but not 1,25(OH)2D. American Journal of Clinical Nutrition 1990;52:701-6.

105. Patel R, Collins D, Bullock S, Swaminathan R, Blake GM, Fogelman I. The effect of season and vitamin D supplementation on bone mineral density in healthy women: a double-masked crossover study. Osteoporosis International 2001;12:319-25.

106. Lips P, Wiersinga A, van Ginkel FC, et al. The effect of vitamin D supplementation on vitamin D status and parathyroid function in elderly subjects. Journal of Clinical Endocrinology & Metabolism 1988;67:644-50.

107. Barger-Lux MJ, Heaney RP, Dowell S, Chen TC, Holick MF. Vitamin D and its major metabolites: serum levels after graded oral dosing in healthy men. Osteoporosis International 1998;8:222-30.

108. Honkanen R, Alhava E, Parviainen M, Talasniemi S, Monkkonen R. The necessity and safety of calcium and vitamin D in the elderly. Journal of the American Geriatrics Society 1990;38:862-6.

109. Chapuy MC, Meunier PJ. Prevention of secondary hyperparathyroidism and hip fracture in elderly women with calcium and vitamin D3 supplements. Osteoporosis International 1996;6:60-3.

110. MacLennan WJ, Hamilton JC. Vitamin D supplements and 25-hydroxy vitamin D concentrations in the elderly. Bmj 1977;2:859-61.

111. Chapuy MC, Chapuy P, Meunier PJ. Calcium and vitamin D supplements: effects on calcium metabolism in elderly people. American Journal of Clinical Nutrition 1987;46:324-8.

112. Chapuy MC, Arlot ME, Duboeuf F, et al. Vitamin D3 and calcium to prevent hip fractures in the elderly women. New England Journal of Medicine 1992;327:1637-42.

113. Sorva A, Risteli J, Risteli L, Valimaki M, Tilvis R. Effects of vitamin D and calcium on markers of bone metabolism in geriatric patients with low serum 25-hydroxyvitamin D levels. Calcified Tissue International 1991;49:S88-9.

114. Vieth R, Chan PC, MacFarlane GD. Efficacy and safety of vitamin D(3) intake exceeding the lowest observed adverse effect level. American Journal of Clinical Nutrition 2001;73:288-94.

115. Gannage-Yared MH, Chemali R, Yaacoub N, Halaby G. Hypovitaminosis D in a sunny country: relation to lifestyle and bone markers. Journal of Bone & Mineral Research 2000;15:1856-62.

116. Goswami R, Gupta N, Goswami D, Marwaha RK, Tandon N, Kochupillai N. Prevalence and significance of low 25-hydroxyvitamin D concentrations in healthy subjects in Delhi. American Journal of Clinical Nutrition 2000;72:472-5.

117. Thomas MK, Lloyd-Jones DM, Thadhani RI, et al. Hypovitaminosis D in medical inpatients New England Journal of Medicine 1998;338:777-83.

118. Semba RD, Garrett E, Johnson BA, Guralnik JM, Fried LP. Vitamin D deficiency among older women with and without disability. American Journal of Clinical Nutrition 2000;72:1529-34.

119. Nagengast FM, Grubben MJ, van Munster IP. Role of bile acids in colorectal carcinogenesis. European Journal of Cancer 1995;31A:1067-70.

120. McSherry C, Cohen B, Bokkenheuser V, et al. Effects of calcium and bile acid feeding on colon tumors in the rat. Cancer Res 1989;49:6039-43.

121. Galloway DJ, Owen RW, Jarrett F, Boyle P, Hill MJ, George WD. Experimental colorectal cancer: the relationship of diet and faecal bile acid concentration to tumour induction. British Journal of Surgery 1986;73:233-7.

122. Hill MJ. Bile flow and colon cancer. Mutation Research 1990;238:313-20.

123. Hill MJ. Bile acids and colorectal cancer: hypothesis. European Journal of Cancer Prevention 1991;1:69-74.

124. Newmark HL, Wargovich MJ, Bruce WR. Colon cancer and dietary fat, phosphate, and calcium: a hypothesis. Journal of the National Cancer Institute 1984;72:1323-5.

125. Van der Meer R, Lapre JA, Govers MJ, Kleibeuker JH. Mechanisms of the intestinal effects of dietary fats and milk products on colon carcinogenesis. Cancer Letters 1997;114:75-83.

126. Kallay E, Bajna E, Wrba F, Kriwanek S, Peterlik M, Cross HS. Dietary calcium and growth modulation of human colon cancer cells: role of the extracellular calcium-sensing receptor. Cancer Detection & Prevention 2000;24:127-36.

127. Kallay E, Kifor O, Chattopadhyay N, et al. Calcium-dependent c-myc proto-oncogene expression and proliferation of Caco-2 cells: a role for a luminal extracellular calcium-sensing receptor. Biochemical & Biophysical Research Communications 1997;232:80-3.

128. Reshef R, Rozen P, Fireman Z, et al. Effect of a calcium-enriched diet on the colonic epithelial hyperproliferation induced by N-methyl-N'-nitro-N-nitrosoguanidine in rats on a low calcium and fat diet. Cancer Res 1990;50:1764-7.

129. Nobre-Leitao C, Chaves P, Fidalgo P, et al. Calcium regulation of coloic crypt cell kinetics: evidence for a direct effect in mice. Gastroenterology 1995;109:498-504.

130. Wargovich MJ, Eng VW, Newmark HL, Bruce WR. Calcium ameliorates the toxic effect of deoxycholic acid on colonic epithelium. Carcinogenesis 1983;4:1205-7.

131. Bird RP, Schneider R, Stamp D, Bruce WR. Effect of dietary calcium and cholic acid on the proliferative indices of murine colonic epithelium. Carcinogenesis 1986;7:1657-61.

132. Appleton GV, Bristol JB, Williamson RC. Increased dietary calcium and small bowel resection have opposite effects on colonic cell turnover. British Journal of Surgery 1986;73:1018-21.

133. Appleton GV, Davies PW, Bristol JB, Williamson RC. Inhibition of intestinal carcinogenesis by dietary supplementation with calcium. British Journal of Surgery 1987;74:523-5.

134. Pence BC, Buddingh F. Inhibition of dietary fat-promoted colon carcinogenesis in rats by supplemental calcium or vitamin D3. Carcinogenesis 1988;9:187-90.

135. Wargovich M, Allnutt D, Palmer C, Anaya P, Stephens L. Inhibition of the promotional phase of azoxymethane-induced colon carcinogenesis in the rat by calcium lactate: effect of simulating two human nutrient density levels. Cancer Lett 1990;53:17-25.

136. Pence BC, Dunn DM, Zhao C, Landers M, Wargovich MJ. Chemopreventive effects of calcium but not aspirin supplementation in cholic acid-promoted colon carcinogenesis: correlation with intermediate endpoints. Carcinogenesis 1995;16:757-65.

137. Karkare M, Clark T, Glauert H. Effect of dietary calcium on colon carcinogenesis induced by a single injection of 1,2-dimethylhydrazine in rats. J Nutr 1991;121:568-77.

138. Wu K, Willett WC, Fuchs CS, Colditz GA, Giovannucci EL. Calcium intake and risk of colon cancer in women and men. Journal of the National Cancer Institute 2002;94:437-46.

139. Heilbrun L, Hankin J, Nomura A, Stemmermann G. Colon cancer and dietary fat; phosphorus; and calcium in Hawaiian-Japanese men. Am J Clin Nutr 1986;1986:306-9.

140. Kampman E, Goldbohm R, Van Den Brandt P, Van't Veer P. Fermented dairy products, calcium, and colorectal cancer in the Netherlands cohort study. Can Res 1994;54:3186-90.

141. Bergsma-Kadijk JA, van 't Veer P, Kampman E, Burema J. Calcium does not protect against colorectal neoplasia. Epidemiology 1996;7:590-7.

142. Martinez ME, Willett WC. Calcium, Vitamin D, and Colorectal Cancer: A Review of the Epidemiologic Evidence. Cancer Epidemiology, Biomarkers & Prevention 1998;7:163-8.

143. Slattery ML, Sorenson AW, Ford MH. Dietary calcium intake as a mitigating factor in colon cancer. American Journal of Epidemiology 1988;128:504-14.

144. Kampman E, Giovannucci E, van 't Veer P, et al. Calcium, vitamin D, dairy foods, and the occurrence of colorectal adenomas among men and women in two prospective studies. American Journal of Epidemiology 1994;139:16-29.

145. Macquart-Moulin G, Riboli E, Cornee J, Kaaks R, Berthezene P. Colorectal polyps and diet: a case-control study in Marseilles. Int J Cancer 1987;40:179-88.

146. Tseng M, Murray SC, Kupper LL, Sandler RS. Micronutrients and the risk of colorectal adenomas American Journal of Epidemiology 1996;144:1005-14.

147. Hyman J, Baron JA, Dain BJ, et al. Dietary and supplemental calcium and the recurrence of colorectal adenomas. Cancer Epidemiology, Biomarkers & Prevention 1998;7:291-5.

148. Almendingen K, Trygg K, Larsen S, Hofstad B, Vatn MH. Dietary factors and colorectal polyps: a case-control study. European Journal of Cancer Prevention 1995;4:239-46.

149. Neugut A, Horvath K, Whelan R, et al. The effect of calcium and vitamin supplements on the incidence and recurrence of colorectal adenomatous polyps. Cancer 1996;78:723-8.

150. Peleg, II, Lubin MF, Cotsonis GA, Clark WS, Wilcox CM. Long-term use of nonsteroidal antiinflammatory drugs and other chemopreventors and risk of subsequent colorectal neoplasia. Digestive Diseases & Sciences 1996;41:1319-26.

151. White E, Shannon JS, Patterson RE. Relationship between vitamin and calcium supplement use and colon cancer. Cancer Epidemiology, Biomarkers & Prevention 1997;6:769-74.

152. Whelan RL, Horvath KD, Gleason NR, et al. Vitamin and calcium supplement use is associated with decreased adenoma recurrence in patients with a previous history of neoplasia. Diseases of the Colon & Rectum 1999;42:212-7.

153. Bonithon-Kopp C, Kronborg O, Giacosa A, Rath U, Faivre J. Calcium and fibre supplementation in prevention of colorectal adenoma recurrence: a randomised intervention trial. European Cancer Prevention Organisation Study Group. Lancet 2000;356:1300-6.

154. Thomas MG, Thomson JP, Williamson RC. Oral calcium inhibits rectal epithelial proliferation in familial adenomatous polyposis. British Journal of Surgery 1993;80:499-501.

155. Hofstad B, Almendingen K, Vatn M, et al. Growth and recurrence of colorectal polyps: a double-blind 3-year intervention with calcium and antioxidants. Digestion 1998;59:148-56.

156. Gregoire RC, Stern HS, Yeung KS, et al. Effect of calcium supplementation on mucosal cell proliferation in high risk patients for colon cancer. Gut 1989;30:376-82.

157. Stern HS, Gregoire RC, Kashtan H, Stadler J, Bruce RW. Long-term effects of dietary calcium on risk markers for colon cancer in patients with familial polyposis. Surgery 1990;108:528-33.

158. Bostick RM, Potter JD, Fosdick L, et al. Calcium and colorectal epithelial cell proliferation: a preliminary randomized, double-blinded, placebo-controlled clinical trial. Journal of the National Cancer Institute 1993;85:132-41.

159. Kleibeuker JH, Welberg JW, Mulder NH, et al. Epithelial cell proliferation in the sigmoid colon of patients with adenomatous polyps increases during oral calcium supplementation. British Journal of Cancer 1993;67:500-3.

160. Cats A, Kleibeuker JH, van der Meer R, et al. Randomized, double-blinded, placebo-controlled intervention study with supplemental calcium in families with hereditary nonpolyposis colorectal cancer. Journal of the National Cancer Institute 1995;87:598-603.

161. Baron JA, Tosteson TD, Wargovich MJ, et al. Calcium supplementation and rectal mucosal proliferation: a randomized controlled trial Journal of the National Cancer Institute 1995;87:1303-7.

162. Armitage NC, Rooney PS, Gifford KA, Clarke PA, Hardcastle JD. The effect of calcium supplements on rectal mucosal proliferation. British Journal of Cancer 1995;71:186-90.

163. Lipkin M, Newmark H. Effect of added dietary calcium on colonic epithelial-cell proliferation in subjects at high risk for familial colonic cancer. New England Journal of Medicine 1985;313:1381-4.

164. Lipkin M, Friedman E, Winawer SJ, Newmark H. Colonic epithelial cell proliferation in responders and nonresponders to supplemental dietary calcium. Cancer Research 1989;49:248-54.

165. Rozen P, Fireman Z, Fine N, Wax Y, Ron E. Oral calcium suppresses increased rectal epithelial proliferation of persons at risk of colorectal cancer. Gut 1989;30:650-5.

166. Barsoum GH, Hendrickse C, Winslet MC, et al. Reduction of mucosal crypt cell proliferation in patients with colorectal adenomatous polyps by dietary calcium supplementation. British Journal of Surgery 1992;79:581-3.

167. Wargovich MJ, Isbell G, Shabot M, et al. Calcium supplementation decreases rectal epithelial cell proliferation in subjects with sporadic adenoma Gastroenterology 1992;103:92-7.

168. Bostick RM, Fosdick L, Wood JR, et al. Calcium and colorectal epithelial cell proliferation in sporadic adenoma patients: a randomized, double-blinded, placebo-controlled clinical trial. Journal of the National Cancer Institute 1995;87:1307-15.

169. Lapre JA, De Vries HT, Termont DS, Kleibeuker JH, De Vries EG, Van der Meer R. Mechanism of the protective effect of supplemental dietary calcium on cytolytic activity of fecal water. Cancer Research 1993;53:248-53.

170. Welberg JW, Kleibeuker JH, Van der Meer R, et al. Effects of oral calcium supplementation on intestinal bile acids and cytolytic activity of fecal water in patients with adenomatous polyps of the colon. European Journal of Clinical Investigation 1993;23:63-8.

171. Lupton JR, Steinbach G, Chang WC, et al. Calcium supplementation modifies the relative amounts of bile acids in bile and affects key aspects of human colon physiology. Journal of Nutrition 1996;126:1421-8.

172. Alder RJ, McKeown-Eyssen G, Bright-See E. Randomized trial of the effect of calcium supplementation on fecal risk factors for colorectal cancer. American Journal of Epidemiology 1993;138:804-14.

173. Curhan GC, Willett WC, Rimm EB, Stampfer MJ. A prospective study of dietary calcium and other nutrients and the risk of symptomatic kidney stones. New England Journal of Medicine 1993;328:833-8.

174. Curhan GC, Willett WC, Speizer FE, Spiegelman D, Stampfer MJ. Comparison of dietary calcium with supplemental calcium and other nutrients as factors affecting the risk for kidney stones in women. Annals of Internal Medicine 1997;126:497-504.

175. Schwarz S, Trivedi BK, Kalantar-Zadeh K, Kovesdy CP. Association of disorders in mineral metabolism with progression of chronic kidney disease. Clin J Am Soc Nephrol 2006;1:825-31.

176. Melamed ML, Astor B, Michos ED, Hostetter TH, Powe NR, Muntner P. 25-hydroxyvitamin D levels, race, and the progression of kidney disease. J Am Soc Nephrol 2009;20:2631-9.

177. Ix JH, Shlipak MG, Wassel CL, Whooley MA. Fibroblast growth factor-23 and early decrements in kidney function: the Heart and Soul Study. Nephrol Dial Transplant 2010;25:993-7.

178. Li YC, Qiao G, Uskokovic M, Xiang W, Zheng W, Kong J. Vitamin D: a negative endocrine regulator of the renin-angiotensin system and blood pressure. J Steroid Biochem Mol Biol 2004;89-90:387-92.

179. Migliori M, Giovannini L, Panichi V, et al. Treatment with 1,25-dihydroxyvitamin D3 preserves glomerular slit diaphragm-associated protein expression in experimental glomerulonephritis. Int J Immunopathol Pharmacol 2005;18:779-90.

180. Makibayashi K, Tatematsu M, Hirata M, et al. A vitamin D analog ameliorates glomerular injury on rat glomerulonephritis. Am J Pathol 2001;158:1733-41.

181. Neves KR, Graciolli FG, dos Reis LM, Pasqualucci CA, Moyses RM, Jorgetti V. Adverse effects of hyperphosphatemia on myocardial hypertrophy, renal function, and bone in rats with renal failure. Kidney Int 2004;66:2237-44.

182. Forman JP, Giovannucci E, Holmes MD, et al. Plasma 25-hydroxyvitamin D levels and risk of incident hypertension. Hypertension 2007;49:1063-9.

183. Krause R, Buhring M, Hopfenmuller W, Holick MF, Sharma AM. Ultraviolet B and blood pressure. Lancet 1998;352:709-10.

184. Pfeifer M, Begerow B, Minne HW, Nachtigall D, Hansen C. Effects of a short-term vitamin D(3) and calcium supplementation on blood pressure and parathyroid hormone levels in elderly women. J Clin Endocrinol Metab 2001;86:1633-7.

185. Sugden JA, Davies JI, Witham MD, Morris AD, Struthers AD. Vitamin D improves endothelial function in patients with Type 2 diabetes mellitus and low vitamin D levels. Diabet Med 2008;25:320-5.

186. Foster MC, Hwang SJ, Larson MG, et al. Cross-classification of microalbuminuria and reduced glomerular filtration rate: associations between cardiovascular disease risk factors and clinical outcomes. Arch Intern Med 2007;167:1386-92.

187. Astor BC, Hallan SI, Miller ER, 3rd, Yeung E, Coresh J. Glomerular filtration rate, albuminuria, and risk of cardiovascular and all-cause mortality in the US population. American journal of epidemiology 2008;167:1226-34.

188. Levey AS, Stevens LA, Schmid CH, et al. A new equation to estimate glomerular filtration rate. Annals of internal medicine 2009;150:604-12.

189. Pocock SJ, Hughes MD. Practical problems in interim analyses, with particular regard to estimation. Controlled Clinical Trials 1989;10:209S-21S.

190. O'Brien PC, Fleming TR. A multiple testing procedure for clinical trials. Biometrics 1979;35:549-56.
